# Development of Continuous Flow Systems to Access Secondary Amines Through Previously Incompatible Biocatalytic Cascades**

**DOI:** 10.1002/ange.202103805

**Published:** 2021-05-19

**Authors:** Ashley P. Mattey, Grayson J. Ford, Joan Citoler, Christopher Baldwin, James R. Marshall, Ryan B. Palmer, Matthew Thompson, Nicholas J. Turner, Sebastian C. Cosgrove, Sabine L. Flitsch

**Affiliations:** ^1^ Manchester Institute of Biotechnology (MIB) &, School of Chemistry The University of Manchester 131 Princess Street Manchester M1 7DN UK; ^2^ Enginzyme AB Tomtebodevägen 6 17165 Stockholm Sweden; ^3^ Lennard-Jones Laboratory School of Chemical and Physical Sciences Keele University Keele Staffordshire ST5 5BG UK

**Keywords:** biocatalytic cascades, continuous flow, oxidation, reductive amination, transamination

## Abstract

A key aim of biocatalysis is to mimic the ability of eukaryotic cells to carry out multistep cascades in a controlled and selective way. As biocatalytic cascades get more complex, reactions become unattainable under typical batch conditions. Here a number of continuous flow systems were used to overcome batch incompatibility, thus allowing for successful biocatalytic cascades. As proof‐of‐principle, reactive carbonyl intermediates were generated in situ using alcohol oxidases, then passed directly to a series of packed‐bed modules containing different aminating biocatalysts which accordingly produced a range of structurally distinct amines. The method was expanded to employ a batch incompatible sequential amination cascade via an oxidase/transaminase/imine reductase sequence, introducing different amine reagents at each step without cross‐reactivity. The combined approaches allowed for the biocatalytic synthesis of the natural product 4O‐methylnorbelladine.

## Graphical abstract

The use of continuous flow reactors enabled previously inaccessible enzyme combinations in multi‐enzyme cascade reactions. This included the combination of oxidases with aminating biocatalysts (transaminase/reductive aminase) and was extended to the continuous in situ biocatalytic generation of amino donors for the reductive aminase.

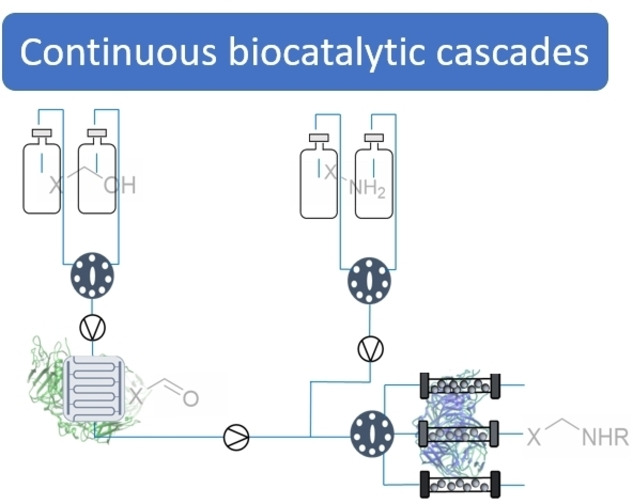

## Introduction

Multi‐step synthesis in biocatalysis has been made more efficient through the utilization of enzymatic cascades. No longer are reactions limited by intermediate isolation, thus significantly reducing time and waste.[^1^, ^2^] The design of multienzyme systems has provided convenient syntheses of a number of high value compounds and are now seen as the method of choice in implementing biocatalytic reactions.[^3^, ^4^] Computer‐aided synthesis planning (CASP) is also poised to revolutionize biocatalytic synthesis planning, allowing in silico prediction of novel biocatalytic cascades.^[5]^ Nevertheless, while biocatalytic cascades are increasingly considered as the method of choice for more complex syntheses, there are still limitations to one‐pot systems due to incompatible enzyme/substrate combinations.

**Figure 1:**
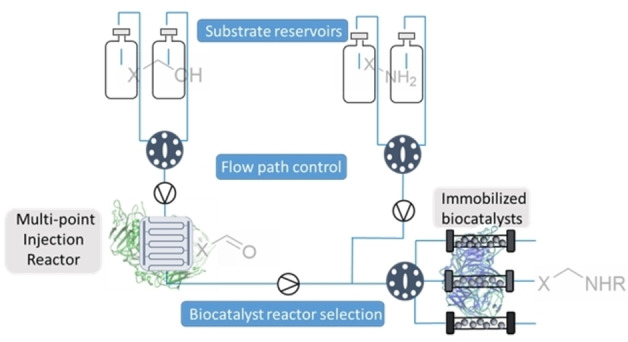
Advanced compartmentalized continuous flow systems can be used to overcome incompatible enzyme combinations/chemistries can deliver the lab‐based technological advancement necessary to fully realize this transformation.

Flow chemistry has seen rapid development in recent years, with the potential to improve about 50 % of chemical processes.^[6]^ Small scale flow systems can be translated into larger scale production with minimal optimization as the systems can be run for longer to increase productivity, a luxury not afforded in batch processes which need to be scaled up.[^7^, ^8^] Another advantage is that continuous flow systems can be composed of different modules (different reactor types), which can enable the combination of a broad range of chemistries that are incompatible under batch conditions (Figure 1).^[9]^ Further to this, the utilization of continuous flow systems facilitates the integration of several reaction steps resulting in telescoped synthetic sequences.^[10]^

Issues that arise with analytical biotransformations not being translatable to scale or different biocatalytic reaction types being incompatible, has led to the emergence of continuous flow biocatalysis as a favorable option to alleviate these issues.^[11]^ Operating under a continuous regime allows for greater control of reaction conditions as well as providing opportunities for inline analysis and purification, as well as automation.[^12^, ^13^] Most flow biocatalysis systems utilize immobilized enzymes, which vastly improves reusability and often increases thermal stability and solvent tolerance.[^14^, ^15^, ^16^] The combination of immobilization and continuous flow reactors means that enzymes can be compartmentalized and segregated, allowing access to previously incompatible reactions in sequential reactor modules. Herein, efforts to exploit the power of biosynthetic cascades with a continuous, compartmentalization approach containing multiple reactor types and a combination of free and immobilized enzyme are described. Issues of cross reactivity and reaction incompatibility in linear enzymatic cascades are addressed whilst also showing compartmentalized flow systems can be used to overcome metabolic flux issues associated with in vitro cascades (Figure 1).^[17]^ The aim was to adopt a modular approach using standard laboratory equipment to generate versatile systems which enable access to a broad range of chemistries.

## Results and Discussion

The reaction cascades described here started with the generation of aldehydes from stable and commercially available alcohols. As aldehydes are versatile yet unstable intermediates, it was thought a continuous flow system that generated this group in situ at high concentrations could allow for a range of subsequent enzymatic modifications. Biocatalytic oxidations are fundamental in transitioning to a bio‐based economy, in particular the use of oxidases which carry out selective oxidation using molecular oxygen as the sole oxidant.[^18^, ^19^] To overcome potential limitations of oxygen concentration, a previously described multipoint injection reactor (MPIR) was employed; it was shown to greatly improve the productivity of oxidase biocatalysts by negating low aqueous oxygen availability through in situ biocatalytic generation from hydrogen peroxide (Table 1).[^20^, ^21^, ^22^] An engineered choline oxidase (AcCO_6_) was initially chosen as an ideal biocatalyst to test in the MPIR due to its broad substrate scope and as it has been applied in biocatalytic cascades.[^23^, ^24^] Using the MPIR, AcCO6 productivity was vastly improved for the oxidation of a number of aromatic and aliphatic primary alcohols with a 58 fold improvement in space time yield and 4 fold improvement in productivity in the oxidation of phenylethanol (see the Supporting Information). The bio‐oxidations were further optimized to ensure no over‐oxidation to the corresponding acid was observed, an issue associated with batch reactions and a previous flow report for this enzyme.^[14]^

**Table 1:** Representation of the MPIR‐packed bed continuous flow system for the biocatalytic synthesis of amines from alcohols.^[a]^

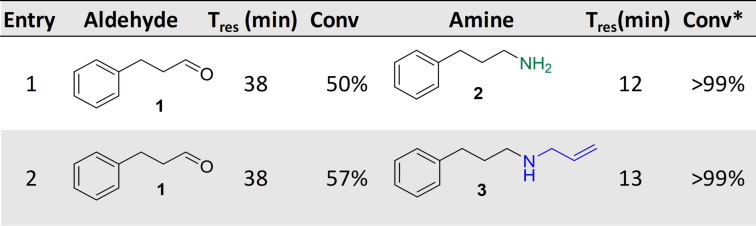

[a] Key: *Steady state conversions determined by GC‐FID and compared to chemical standards.

Following the successful implementation of the MPIR for the generation of aldehydes, the system was expanded by carrying out subsequent continuous bio‐reductive aminations. Amines are important molecules in synthetic chemistry, with a significant proportion of the reactions performed by medicinal chemists reported to be associated with amino‐group chemistry.^[25]^ Biocatalytic amine synthesis has been advanced by transaminases and imine reductases/reductive aminases (IREDs/RedAms), with the use of aminating biocatalysts providing selective and sustainable options for the synthesis of chiral amines.[^26^, ^27^] One of the most frequently applied aminating enzymes in flow is transaminase.[^28^, ^29^] Therefore, the transaminase from *Bacillus megaterium* (*Bm*TA) was immobilized and loaded it into a packed‐bed reactor.[^23^, ^30^] This was connected to the output of the MPIR (with inline mixing via a micro static mixer), generating an MPIR‐packed bed system (MPBS). Pleasingly, full conversion to the corresponding primary amine **2** was achieved at steady state and maintained for four hours (STY: 1.58 g L^−1^ h^−1^).

For the generation of secondary amines such as **3** from aldehyde **1**, RedAm and glucose dehydrogenase (GDH) combinations can be used and have also been demonstrated in flow systems previously.[^14^, ^31^, ^32^] Here, the reductive aminase from *Ajellomyces dermatitidis* (*Ad*RedAm, 200 mg, 10 wt %) and the GDH from *Bacillus megaterium* (*Bs*GDH, 10 mg, 10 wt %) were immobilized and both loaded into a packed bed reactor. Pleasingly, this also gave full conversion of the in situ generated hydrocinnamaldehyde **1** to the corresponding *N*‐allyl amine **3** at steady state which was maintained for four hours (STY: 2.1 g L^−1^ h^−1^) (Table 1).

**Scheme 1:**
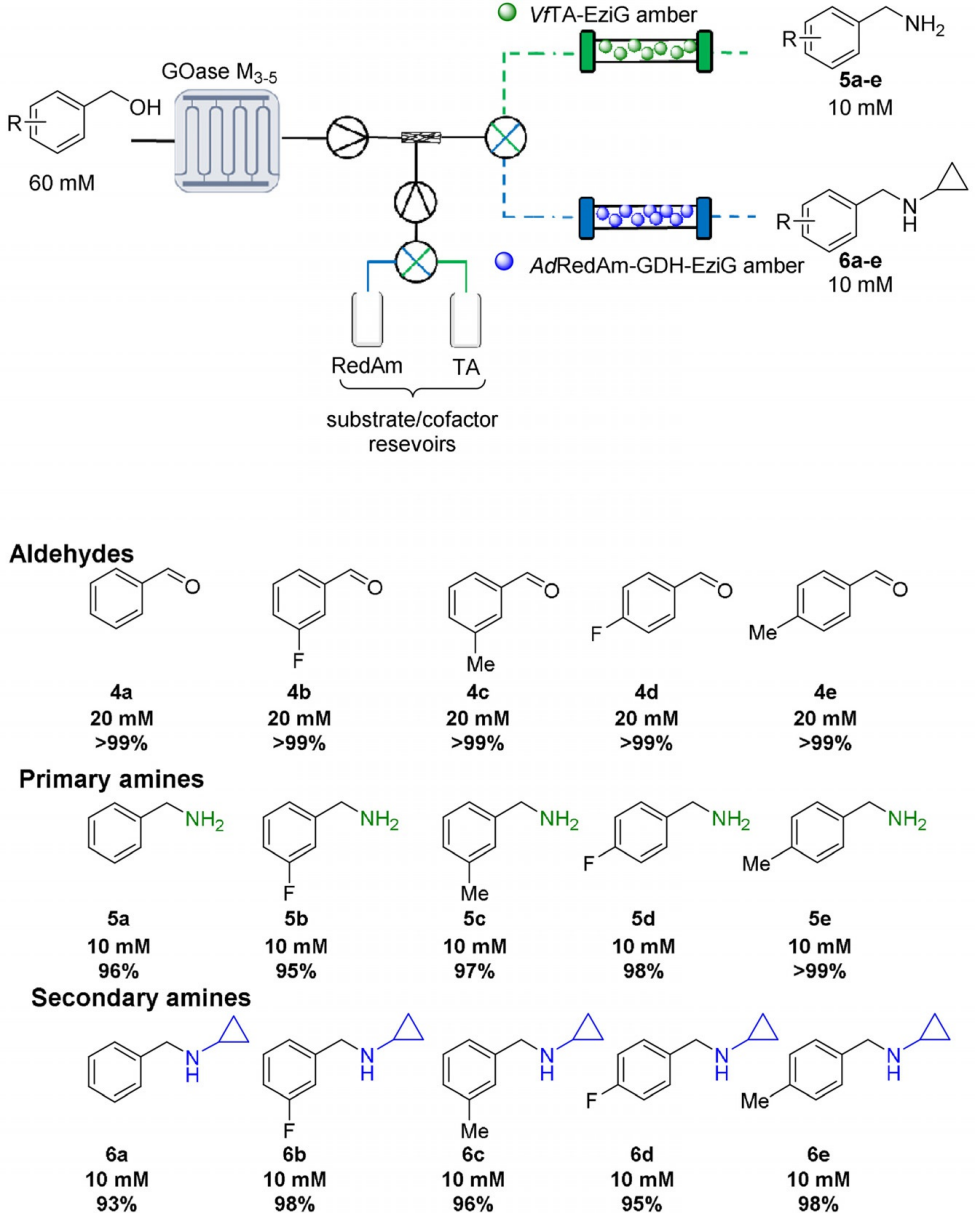
GOase M_3‐5_ and *Ad*RedAm/*Vf*TA were applied in the MPBS continuous flow system and used for cascade screening. Steady state conversions determined by GC‐FID analysis. Triangle in circle: pump; cross in circle: mixing valve.

Following the optimization of AcCO_6_‐amination cascades in continuous flow, the combination of previously batch‐incompatible enzyme systems was investigated. Galactose oxidase (GOase), a copper‐dependent alcohol oxidase, has been shown to be a useful tool in biocatalysis with a number of variants displaying a broad substrate scope.[^33^, ^34^, ^35^] Using GOase instead of choline oxidase would lead to a panel of benzylic amines as shown in Scheme 1. However, due to the reactive copper center in GOase, amines can inhibit the biocatalyst meaning one‐pot batch reactions are not feasible with aminating enzymes. Indeed, when the cascades were carried out in batch, performance as expected was poor, with no observable conversion to the corresponding amines (see Supporting Information).

To overcome these issues the use of compartmentalized MPBS was investigated. Pleasingly, extremely efficient cascades were observed with >95 % conversion achieved in the reductive amination of five benzaldehydes **4** (generated in situ by GOase in the MPIR, Scheme 1), using the transaminase from *Vibrio fluvialis* (*Vf*TA) and *Ad*RedAm to generate the primary **5** and secondary amines **6**, respectively. Fresh samples of immobilized enzyme preparations were used for each of the described systems to avoid the need to study long term stability between experiments. Importantly, due to immobilization of the enzymes, several substrates could be screened using the same columns. This approach efficiently surveyed the activity of *Vf*TA and *Ad*RedAm in the reductive amination of the benzaldehyes **4 a**–**e** (20 mM: five or 10 equiv. amine). A total system residence time of <38 mins was enough to obtain >90 % conversion in all instances. A panel of 10 primary **5 a**–**e**/secondary amines **6 a**–**e** (10 mM) were generated merely by changing the flow path and the initial benzyl alcohol, an approach far less time consuming than the preparation of individual batch reactions and enzyme preparations. To further increase efficiency and automation, three‐way switching valves (including a retrofitted HPLC waste valve) were added to the system (Scheme 1); this enabled exquisite control of the flow path without the laborious need of changing lines or columns. Using this approach, the system could be quickly switched between “RedAm” and “TA” flow paths without intermittent washing steps, thus vastly improving efficiency. The same approach was also used for substrate feeding to the MPIR again greatly improving the efficiency of the system. These cascades represent the first demonstration of continuous biocatalyst substrate screening. This streamlined flow system has the potential to complement current high throughput biocatalytic screening methods in future,^[36]^ and ultimately facilitate automation for biocatalytic cascade discovery in a highly efficient manner.

Following the successful implementation of the oxidase‐bioamination cascades, the wide scope of the system was demonstrated with in situ amine donor generation in a TA‐RedAm cascade (Table 2). Combinations of TA and RedAm/IRED in batch are not compatible because both enzymes use amine substrates with potential for cross reactivity. Hence, the in situ generation of primary amines by TAs for use in subsequent reductive aminations by RedAm/IRED has yet to be explored. For the initial flow bioamination cascade, *Bm*TA and *Ad*RedAm (with *Bs*GDH) were immobilized on EziG amber support (200 mg 10 wt %) and placed in separate packed bed reactors, to prevent the previously mentioned potential issues for cross amination. Initially, a solution containing butanal (40 mM), racemic alanine (400 mM) and PLP (1 mM) was passed through the TA module, achieving full conversion to butylamine **7** at steady state with a residence time of 12 minutes. The effluent of this reaction was mixed via a microstatic mixer (see Supporting Information) with another solution containing cyclohexanone (10 mM), NADP^+^ (1 mM) and glucose (50 mM). Initial results with *Ad*RedAm showed about 20 % conversion at steady state, with four equivalents of amine **7** necessary (20 mM butylamine **7**, and 5 mM ketone in RedAm module). To further improve the productivity of this reaction a more suitable RedAm was selected from a metagenomics panel.^[37]^ The RedAms were identified using a previously described screen containing 384 different enzymes, which gives a colourimetric indication of activity using a coupled diaphorase system (see supporting information and references).[^37^, ^38^] For the generation of *N*‐butylcylohexlyamine **10**, IR‐79 was selected and immobilized on the EziG amber support (100 mg, 10 wt %), with 85 % conversion to the secondary amine **10** observed and maintained for three hours at steady state (Table 2). This represented a total system residence time of 21 mins (STY: 1.87 g L^−1^ h^−1^), and only required two equivalents of butylamine **7** (20 mM butylamine and 10 mM ketone in RedAm module). This approach was expanded to generate *N*‐(2‐phenyl)ethyl cyclohexylamine **11** using another metagenomic RedAm (IR‐23), again only requiring two equivalents of amine with a system residence time of 24 mins (STY: 2.53 g L^−1^ h^−1^). A third approach was then used to generate *N*‐benzylcyclohexylamine **12** with the transaminase from *Pseudomonas putida* (*Pp*TA)^[39]^ mediated transamination of benzaldehyde followed by reductive amination with cyclohexanone using IR‐79. Pleasingly 95 % conversion to **12** was achieved and maintained for four hours with a system residence time of 24 mins (STY: 2.24 g L^−1^ h^−1^). Again, the implementation of several switching valves allowed rapid change of flow path and enabled the generation of three RedAm products (**10**, **11**, **12**) without isolation or purification of any intermediates.

**Table 2:** Transaminase‐RedAm cascades.^[a]^

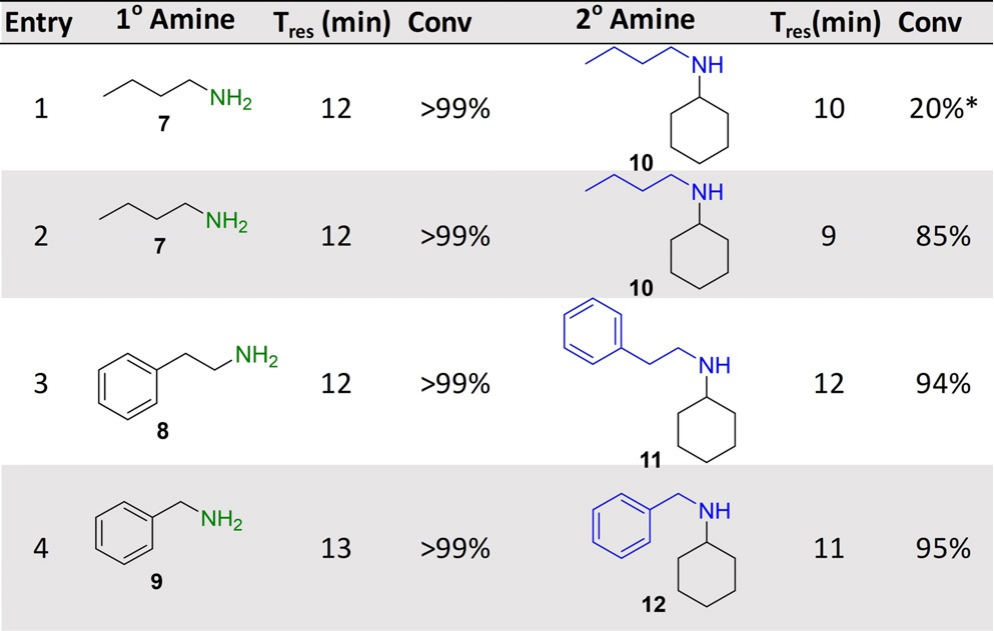

[a] Cascades were applied in a multi packed‐bed reactor system with switching valves enabling efficient control of flow path. Conversions to primary and secondary amines were calculate by comparison of GC‐FID spectra with product standards. Key: *Reductive amination carried out by *Ad*RedAm.

De novo designed biocatalytic cascades are often limited to 2–4 steps due to incompatibility issues as discussed above, which become more limiting as the number of steps increases. After demonstrating that MPBS could over‐come these issues, a compartmentalized six‐enzyme cascade was targeted, highlighting that multiple reactor technologies can be combined to enable previously inaccessible reaction sequences. A synthetic sequence that was able to convert primary alcohols into amines via an oxidase‐TA sequence, which could then be used as the amine substrate for a RedAm reaction was envisaged (Table 3). Using the compartmentalized MPBS, AcCO6 efficiently oxidized phenylethanol (120 mM). The resulting aldehyde **14** was mixed with racemic alanine (400 mM) and PLP (1 mM) via a microstatic mixer, which then passed through immobilized *Bm*TA forming 2‐phenylethylamine **8**. The effluent from this reaction was mixed with a RedAm line (glucose (50 mM), NADP^+^ (1 mM), cyclohexanone (10 mM)) and passed through immobilized IR‐79 to quantitatively generate **11** under continuous conditions. Pleasingly, amine donors were generated in two biocatalytic steps from the alcohols, which then fed directly into the RedAm reactors that successfully utilized the in situ generated amine substrates. All three examples reached steady state conversions of >90 %.

**Table 3:** A six enzyme continuous cascade used for the synthesis of asymmetric amines.^[a]^

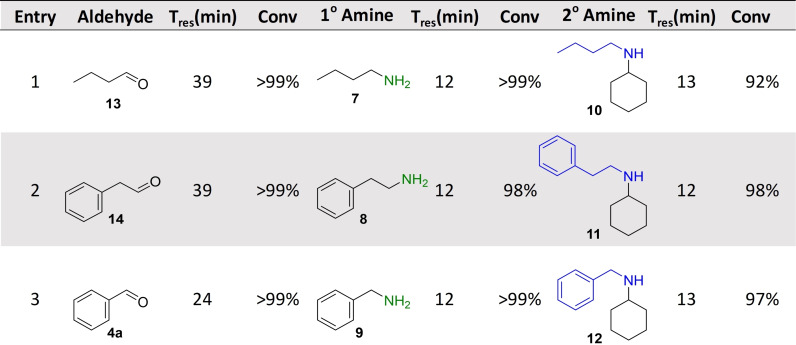

[a] Conversion was determined by GC‐FID.

**Scheme 2:**
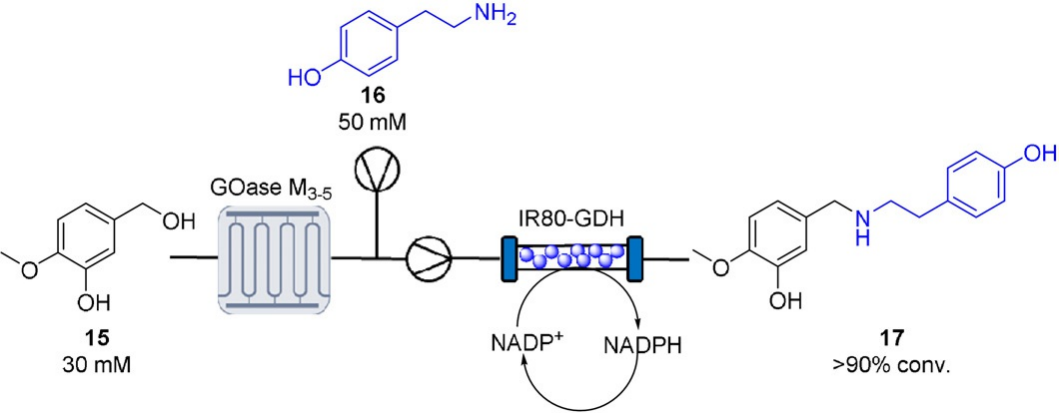
Continuous oxidase‐RedAm cascade for the synthesis of 4O‐methylnorbelladaine 17.

To highlight the benefits of this system in target synthesis, a continuous biocatalytic synthesis of 4O‐methylnorbelladine **17** via an Oxidase‐RedAm cascade was designed (Scheme 2).^[5]^ 4O‐Methylnorbelladine is an important precursor in the synthesis of a number of pharmacologically active alkaloid scaffolds, such as the dementia drug galantamine.[^40^, ^41^] For this cascade, it was thought tyramine **16** could be generated via a continuous oxidase‐TA cascade. Choline oxidase activity was tested against tyrinol in the MPIR. The resulting biogenic aldehyde is a key intermediate in the biosynthesis of benzylisoquinoline alkaloids and often requires multistep syntheses.[^42^, ^43^] Despite the biooxidation proving highly efficient (120 mM substrate loading; >90 % conversion; STY: 8.37 g L^−1^ h^−1^; productivity: 5.1 g_product_ g_enzyme_^−1^) tyramine was commercially available at a lower cost than the alcohol so this step was not necessary.

For the reductive amination of isovanillin and tyramine **16**, the previously described high throughput colorimetric assay was used to identify potential RedAms.^[38]^ From this screen IR‐80 was selected and the reaction validated with an analytical scale biotransformation. The product **17** was found to be insoluble in aqueous buffer and proved difficult to analyze by HPLC. As an alternative, the crude biotransformation was filtered, and the residue suspended in DMSO/MeOH for MALDI analysis with an observed *m*/*z* of 274.2 corresponding to the protonated adduct of the product **17** (see Supporting Information). Encouraged by this result, IR‐80 was immobilized on EziG amber (150 mg, 10 wt %) for translation into continuous flow (Scheme 2). To implement a cascade for this synthesis, a GOase M_3‐5_ (5 mg mL^−1^) mediated biooxidation of isovanilyl alcohol **15** (30 mM) was carried out in the MPIR. The effluent was mixed with the RedAm feed (tyramine **16** (50 mM), NADP^+^ (0.1 mM) and glucose (50 mM)) which was flowed through an IR‐80 module to quantitatively generate **17** for four hours with a system residence time of 36 min (STY: 2.26 g L^−1^ h^−1^). Filtration of the effluent allowed recovery of the excess tyramine and provided a simple purification method for the isolation of **17**.

## Conclusion

For the first time it has been shown that previously incompatible enzyme cascades can be run continuously, greatly improving the metrics (up to 58‐fold for STY and 4‐fold for enzyme productivity) when compared to the equivalent batch reactions. This system is extremely versatile with several enzyme combinations tested that enabled the generation of a range of amine intermediates. The reactors were also extended to demonstrate proof‐of‐concept, high throughput reaction screening in continuous flow. A continuous synthesis of the biologically relevant molecule 4O‐methylnorbelladine was also successfully demonstrated. Using a continuous approach allows many additional benefits to those discussed here to further improve biocatalytic reaction efficiency. In particular, recycling of co‐factors is made operationally more simple with flow systems which significantly reduces the economic impact.[^29^, ^44^] Furthermore, combination with automated high throughput biocatalyst screening,^[36]^ and computer aided synthesis planning,^[5]^ paves the way for fully autonomous biocatalytic synthesis.

## Conflict of interest

Matthew Thompson is an employee for EnginZyme, and provided the immobilisation support used in this study.

## Supporting information

As a service to our authors and readers, this journal provides supporting information supplied by the authors. Such materials are peer reviewed and may be re‐organized for online delivery, but are not copy‐edited or typeset. Technical support issues arising from supporting information (other than missing files) should be addressed to the authors.

Supporting Information
