# Supplementary material for: Development of Continuous Flow Systems to Access Secondary Amines Through Previously Incompatible Biocatalytic Cascades
Source: Angew Chem Weinheim Bergstr Ger. 2021 May 19;133(34):18808–13. doi: 10.1002/ange.202103805 (PMC10947180; doi:10.1002/ange.202103805)
Supplement: Supplementary file 1 — Supporting Information [file ANGE-133-18808-s001.pdf]

Angewandte Chemie
Eine Zeitschrift der Gesellschaft Deutscher Chemiker

Supporting Information

Development of Continuous Flow Systems to Access Secondary Amines Through Previously Incompatible Biocatalytic Cascades**

Ashley P. Mattey, Grayson J. Ford, Joan Citoler, Christopher Baldwin, James R. Marshall, Ryan B. Palmer, Matthew Thompson, Nicholas J. Turner, Sebastian C. Cosgrove, and Sabine L. Flitsch**

ange_202103805_sm_miscellaneous_information.pdf

SUPPORTING INFORMATION

Materials
All chemicals were purchased from Sigma Aldrich unless stated otherwise. EziG^{TM} amber was provided by Enginzyme. NADP$^+$ was provided by Prozomix ltd.

Metagenomic IRED sequences[1]

>p-IR23, CfIRED (*Cystobacter ferrugineus*, A0A1L9AVJ5_9DELT) (24)
MGSSHHHHHHSSGLVPRGSHMKPGISVLGTGRMGSALVGAFLKQGYNVAVWNRTKSKCA
P LAALGARVATTVRDAVADAEVVVVNVNDYVTSEALLRQDDVTKGLRGKLIVQLTSGSPRQ
AREMAAWARQHELQYLDGAIMGTPNFIGEPGGTILYSGPGALFEKYKPVLLVLGGNSLHV
GSDVGHASALDSALLSFLWGSMFGVLQAVSVCEAEGLPLGAYMEYVQATKPMVDGAVTD
F VKRIQTGRFAGDEKTLATVEAHHGALRHLIELCEEHGIHHAVPAAFGQLFQAALQAGHAQ
DDFAVLNKFMK*

>p-IR79 (*Streptomyces chattanoogensis*, A0A0N0XYF8_9ACTN)
MGSSHHHHHHSSGLVPRGSHMTDNKVGSSHDTAGTRPLTLLGLGAMGAALGHAWLAAGP
LTVWNRTPGRAEPLAAQGAQVAGTAAEAVAANRLVVVCLLNDDTLGETLDGVDLAGKDLV
NLTTSTPGQARVRAEWARERGARFLDGGIMAVPPMIGAPGSGASVFYSGSRALFDAHAEA
LAVPAGTAYVGEDPGFAALHDVALLSAMYGMFAGVAHAFALIRKEEIPPKDFAPLLSGWL
TAMTSSVHQAAAQLESGDYTKNVVSNLAMQVAGSETLLGTAEEQGVSAELLTPYLALMER
RLAEGHGDEDTTGLIDLLVRRTGR*

>p-IR80 (*Synechococcus elongatus*, A0A0H3K567_SYNP6)
MGSSHHHHHHSSGLVPRGSHMVGEQETRMRCGLIGTGLLGTAIAERLLTVGQLLTVWNRT
AERSQPLVALGATIAPTPAALLADCEVCLLLLSDAEAIAATLLTEESRSQLVGKTIIQMG
TISPAESRAIADQIAAAGGQYLEAPVLGSLPEARNGTLIVMVGAEPAVFEQWRSLLCHLS
PEPEWIGPIGTAATLKLALNQLIGSLTSAFGGSLALLQRSGLAVEPFMAILRQSALYAPT
FDKKLSRLLSHQYDNPNFPTTHLAKDLRLFRETAADLGITTDAVEGVESIVQKAIAQGWG
DQDYSALYEAINPDSN*

Biocatalyst production
AcCO$_6$, GOase M$_{3-5}$ and *Ad*RedAm were provided by Prozomix ltd. in the form of freeze dried lysate.

Enzyme expression

All IREDs and TAs were expressed by transforming *E. coli* BL21 (DE3) chemically competent cells with plasmids containing the gene sequences of interest. The transformed cells were grown overnight at 37 °C and 200 rpm in 50 mL falcon tubes containing 20 mL of LB medium (1% tryptone, 0.5% yeast extract, 1% NaCl) with kanamycin or ampicillin, depending on the plasmid. The following day, 2 L baffled flasks supplemented with 600 mL LB medium and antibiotic and were inoculated with the overnight cultures (1:100 v/v). At OD 0.6-0.8, the cells were inoculated with IPTG to a final concertation of 0.5 mM and incubated overnight at 20 °C and 200 rpm. Cells were harvested by centrifugation at 4000 rpm for 20 min and stored at -20 °C for further use.

Lysate preparation

Cell pellets were resuspended in Tris-HCl buffer (25 mM, pH 7) at a concentration of 200 mgcww/mL. Subsequently, cells were lysed by ultra-sonication (20 s ON, 20s OFF, 20 cycles) using a Soniprep 150 (MSE UK Ltd.) and centrifuged at 18,000 rpm for 30 min to remove the cell debris. Supernatant was snap-frozen in liquid N_2 and stored at -80 °C for further use.

Biocatalyst	%Expression
$AcCO_6$	10
GOase M_{3-5}	7
BmTA	9
*Pp*TA	10
*Ad*RedAm	12
IR-80	9
IR-79	10
IR-23	9

Table S1: %Expression of each biocatalyst was calculated by purifying a specific amount of freeze dried lysate and measuring amount of purified protein by nanodrop/BCA assay.

Enzyme immobilization

Specific amounts of freeze dried lysate was suspended in immobilization buffer (Tris-HCl (20 mM), NaCl (300 mM), imidazole (25 mM), pH 7) to give a final concentration of 10 mg mL^{-1}. Amounts of EziG amber beads were initially washed with immobilization buffer on an orbital shaker for 10 mins. After this, the beads were left to settle and the supernatant discarded. Biocatalyst lysate was then added to the beads and mixed *via* an orbital shaker for 1 h. The beads were left to settle, then an aliquot of the supernatant was taken prior to removal for SDS-PAGE and absorbance measurements to calculate biocatalyst loading on the support. The remaining beads were washed with Tris-HCl buffer (25 mM, pH 7, NaCl (300 mM)) to remove any weakly bound protein.

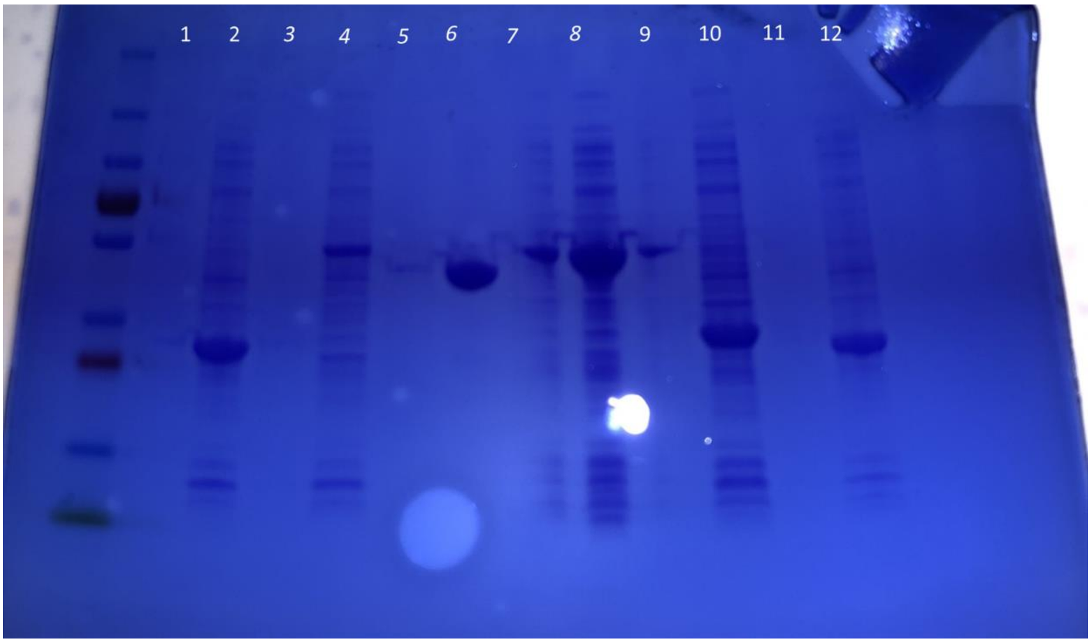

Figure S1: SDS Gel of aliquots from pre and post immobilisation samples (1: IR23 post, 2: IR23 pre, 3: *Bm*TA post, 4:*Bm*TA pre, 5: *Vf*TA post, 6: *Vf*TA pre, 7:*Pp*TA post, 8: *Pp*TA pre, 9: IR79 post, 10: IR79 pre, 11: IR80 post, 12: IR80 pre).

Continuous flow reactions

Continuous flow reactions were performed using the following equipment: Eldex Optos series pumps model 2SM (flow rate 0.003-5.000 mL min^{-1}) and PTFE tubing (I.D 1/16") were purchased from Cole Parmer (Cambridge, UK). Asia flow chemistry 2 module syringe pump (flow rate 0.001-10.000 mL min^{-1}) was purchased from Syrris, each module was equipped with a 500 uL and 1 mL syringe. Stainless steel and PEEK fittings were purchased from Swagelok (Manchester, UK). A Gilson FC204 110/220V fraction collector was purchased from Gilson (Dunstable, UK). An SSI flow-through back-pressure regulator (5-75 psi) was purchased from Sigma-Aldrich (Gillingham, UK). The Perspex multi-point injection reactor, as described previously,[2] was produced by Prof Nik Kapur at the iPRD (University of Leeds, UK). Omnifit manual three-way switching valves were purchased from Fisher scientific UK. Kinesis microstatic mixers (for 1/16" tubing) were purchased from Fisher scientific UK.

Analytical methods

GC-FID
HP-1 (agilent) column (30 m × 0.32 mm × 0.25 µm Method: 40 °C to 325 °C at 30 °C min^{-1}, injector 250 °C, detector 250 °C)

NMR
^{1}H-NMR and ^{13}C-NMR spectra were recorded on a Bruker-400 instrument at 293.8K.

MALDI-ToF
Crude reaction mixtures (0.5 µL) were spotted on target plates, then mixed with THAP matrix (0.5 µL, 10 mg mL^{-1} solution in acetone) and dried in ambient temperature. The product was analysed in positive mode on a Bruker Ultraflex MALDI-ToF instrument.

Metrics

Space-time yield (STY): For a continuous flow reaction, STY is calculated based on the reactor volume, as the amount of product per unit of time per unit of reactor volume (e.g. g L^{-1} h^{-1}). STY was calculated by summing the amounts of product obtained in each of the time points at steady state and dividing them by the total amount of time at steady state and the reactor volume.

Biocatalyst productivity is defined as the amount of product per unit of biocatalyst (e.g. $g_{product}$ g_{enzyme}^{-1}). Biocatalyst productivity was calculated by summing the amounts of product obtained in each of the time points at steady stage and dividing them by the amount of enzyme used during steady state.

Multipoint injection reactor: Choline oxidase optimization

Optimized conditions: the reaction mixture was made into three separate solutions, solution one (Choline oxidase $AcCO_6$ lysate (20 mg mL^{-1}), catalase (0.1 mg mL^{-1})), solution two (phenylethanol (60 mM), H_2O_2 (60 mM)) and solution three (H_2O_2 (240 mM), antifoam (0.01%)). Solutions one two and three were pumped at equal flow rates of 22 µL min^{-1} giving a flow rate in the reactor of 66 µL min^{-1}, affording a t_{res} of 39 minutes. The effluent was collected in 2.6 mL volumes every 39 minutes *via* the fraction collector, corresponding to individual reactor volumes. Each fraction was extracted into ethyl acetate to stop the reaction, and analyzed *via* GC-FID.

Multipoint injection reactor: GOase $M_{3\text{-}5}$

Optimized conditions: The reaction mixture was made into three separate solutions, solution one (GOase $M_{3\text{-}5}$ lysate (10 mg mL^{-1}), HRP (0.1 mg mL^{-1}), catalase (0.1 mg mL^{-1})), solution two (3-F-BenOl (60 mM), H_2O_2 (60 mM)) and solution three (H_2O_2 (240 mM), antifoam (0.01%)). The three solutions were pumped at equal flow rates of 36 µL min^{-1}, giving a flow rate in the reactor of 108 µL min^{-1}, affording a t_{res} of 24 minutes.

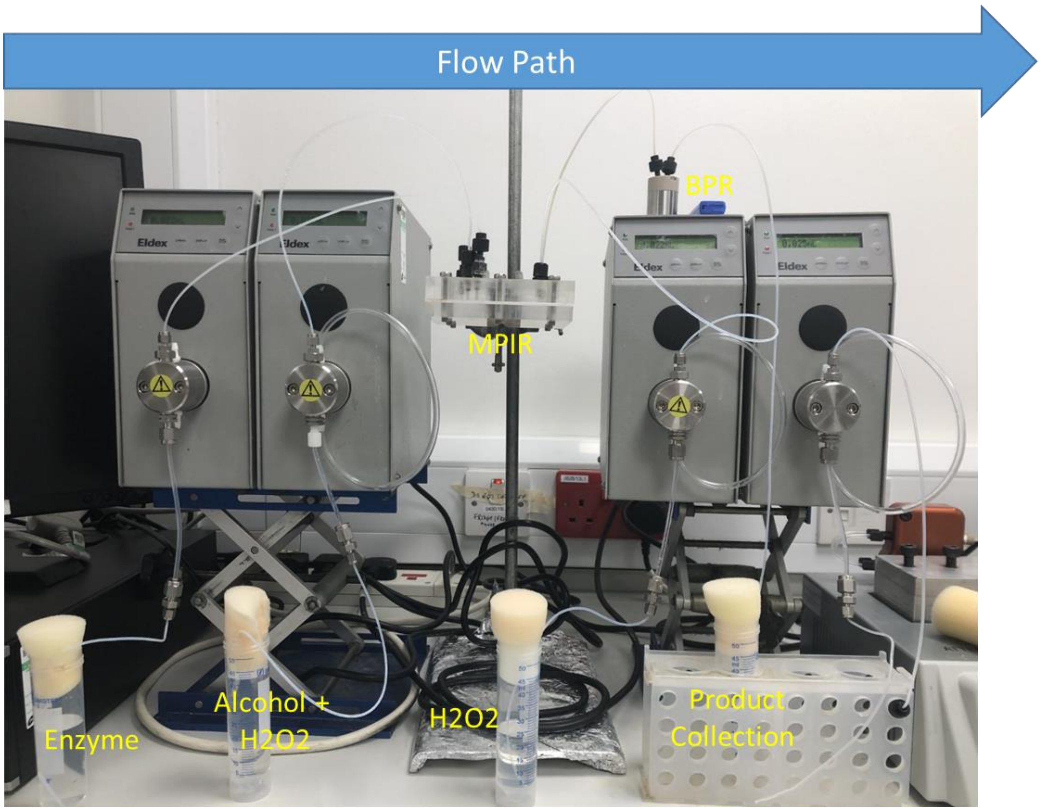

Figure S2: Lab set up of multipoint injection reactor to carry out in flow bio-oxidations.

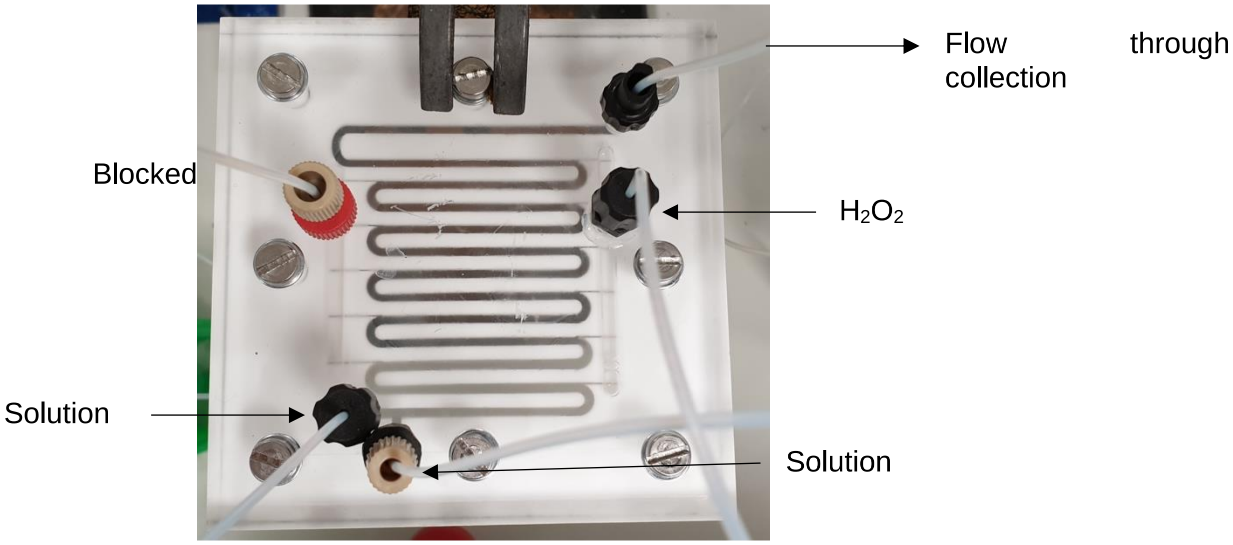

Figure S3: Picture of multipoint injection reactor (total vol. 2.6 mL), with different entry points illustrated.

Table S2: Results of choline oxidase flow optimization reactions.

H2O2 Eq.	Residence time (min)	pH	Conversion	
			Aldehyde	Acid
2	24	8	20	5
3	24	8	26	9
4	24	8	33	13
	39	8	65	4
	39	7	94	0

[a]conditions: AcCO$_6$ (20 mg mL^{-1}), Phenylethanol (60 mM), H_2O_2, KPi buffer (100 mM, pH 7.4), antifoam (0.01%) [b] steady state conversion determined by GC-FID analysis.

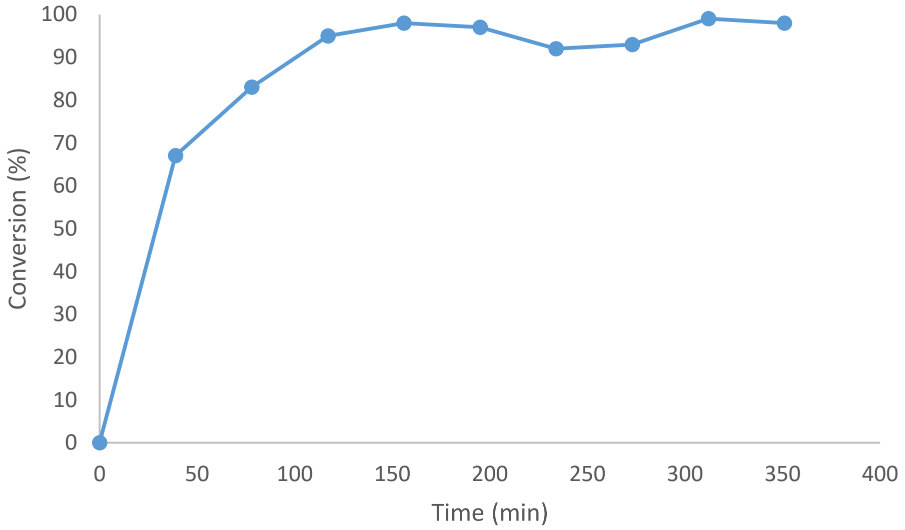

Figure S4: Continuous bio-oxidation of phenylethanol by AcCO$_6$ in the MPIR under optimized conditions.

Batch scale up of AcCO$_6$ bio-oxidation of phenylethanol

To compare the metrics of batch and flow for bio-oxidations with AcCO$_6$ a 35 mL scale biotransformation was prepared. The following reactants and reagents were added to a 50 mL flask to a total volume of 35 mL: AcCO$_6$ (25 mg mL^{-1}), catalase (0.1 mg mL^{-1}), phenylethanol (100 mM), KPi buffer (100 mM, pH 7.4, 10% DMSO). The flask was incubated at 30 °C for 16 h. After this a 500 μL aliquot was taken and extracted into ethyl acetate and conversion to the corresponding aldehyde was determined by GC-FID analysis.

One pot batch GOase-TA/IRED (Free enzyme) reactions

A reaction containing GOase (2mg mL^{-1}), HRP and catalase (both 0.1 mg mL^{-1}), 3F-BenOl (20 mM), *Ad*RedAm (2 mg mL^{-1}), *Bs*GDH (0.1 mg mL^{-1}) NADP$^+$ (0.5 mM), glucose (50 mM), cyclopropylamine (100 mM) and $CuSO_4$ (0.05 mM) in NaPi buffer (100 mM, pH 7.4) was analysed *via* GC-FID, with no conversion observed.

A reaction containing GOase (2mg mL^{-1}), HRP and catalase (both 0.1 mg mL^{-1}), 3F-BenOl (20 mM), *Vf*TA (1 mg mL^{-1}), alanine (200 mM) and $CuSO_4$ (0.05 mM) in NaPi buffer (100 mM, pH 7.4) was analysed *via* GC-FID, with no conversion observed.

Single step continuous biotransformations in packed bed reactors

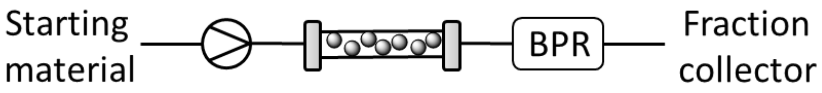

Figure S5: Schematic representation of single enzymatic transformation in continuous flow.

Packed bed continuous reactions: Metagenomic IREDs

Initially, three RedAms were selected from a metagenomic panel[1] and co-immobilized with *Bs*GDH then packed into glass columns for continuous flow reactions. Fractions were collected every 24 mins (equating to approximately two reactor volumes) and ran for 4 h. All RedAm flow reactions were carried out in KPi buffer (100 mM, pH 7.5) to ensure compatibility with other reaction buffers.

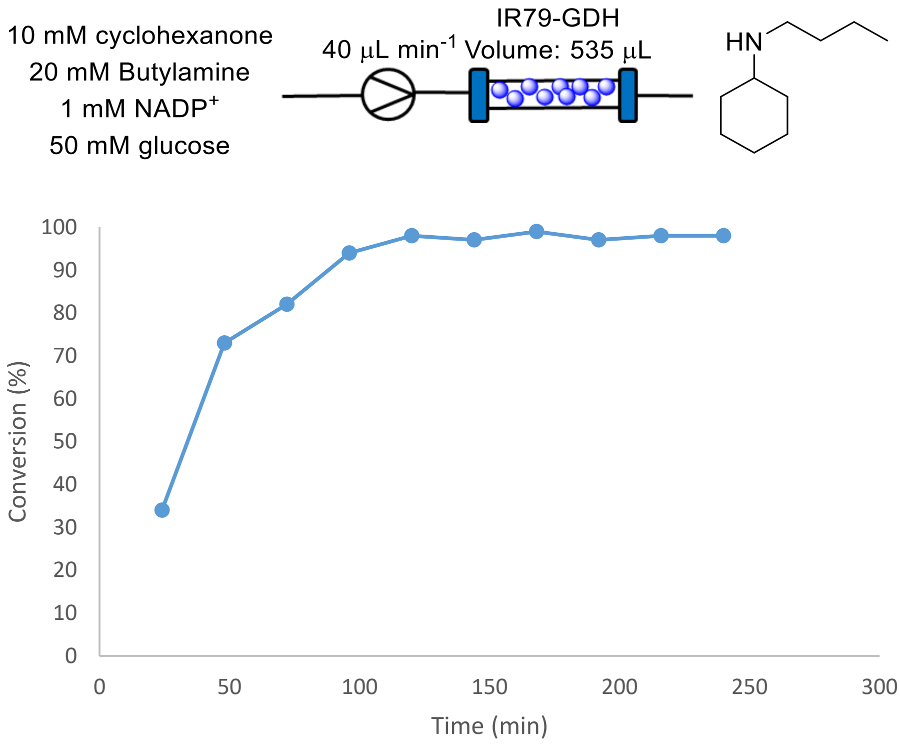

Figure S6: Continuous production of *N*-butylcyclohexanamine with co-immobilized IR-79 and *Bs*GDH. Each data point represents a single analysis.

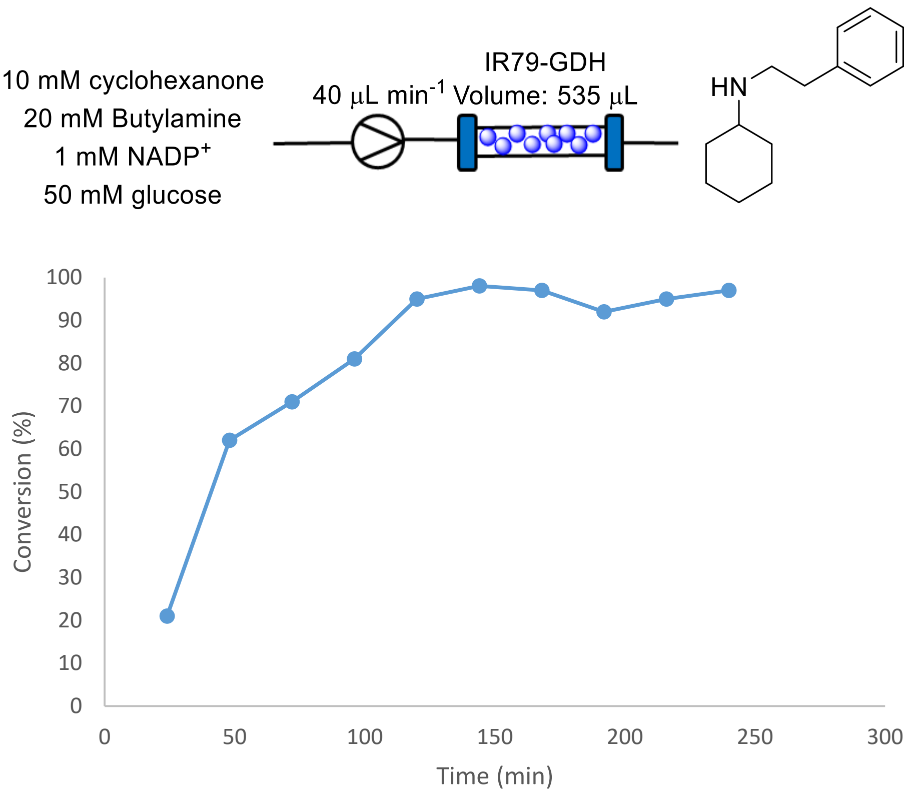

Figure S7: Continuous production of *N*-phenyethylcyclohexanamine with co-immobilized IR-23 and *Bs*GDH. Each data point represents a single analysis.

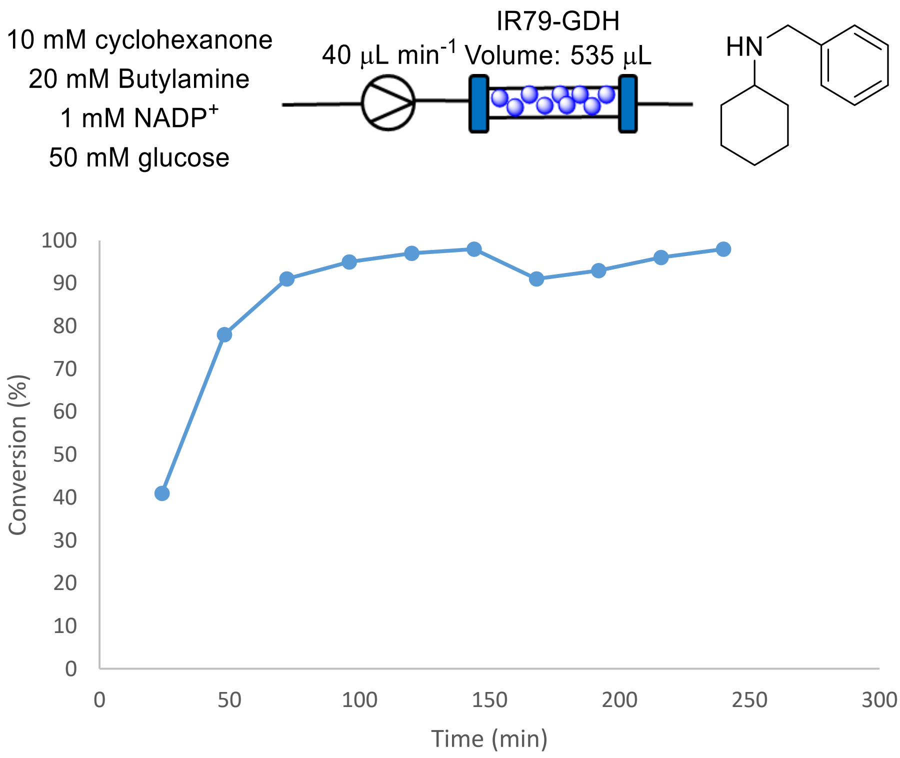

Figure S8: Continuous production of *N*-benzylcyclohexanamine with co-immobilized IR-79 and *Bs*GDH. Each data point represents a single analysis.

MPIR-Packed bed continuous reactions

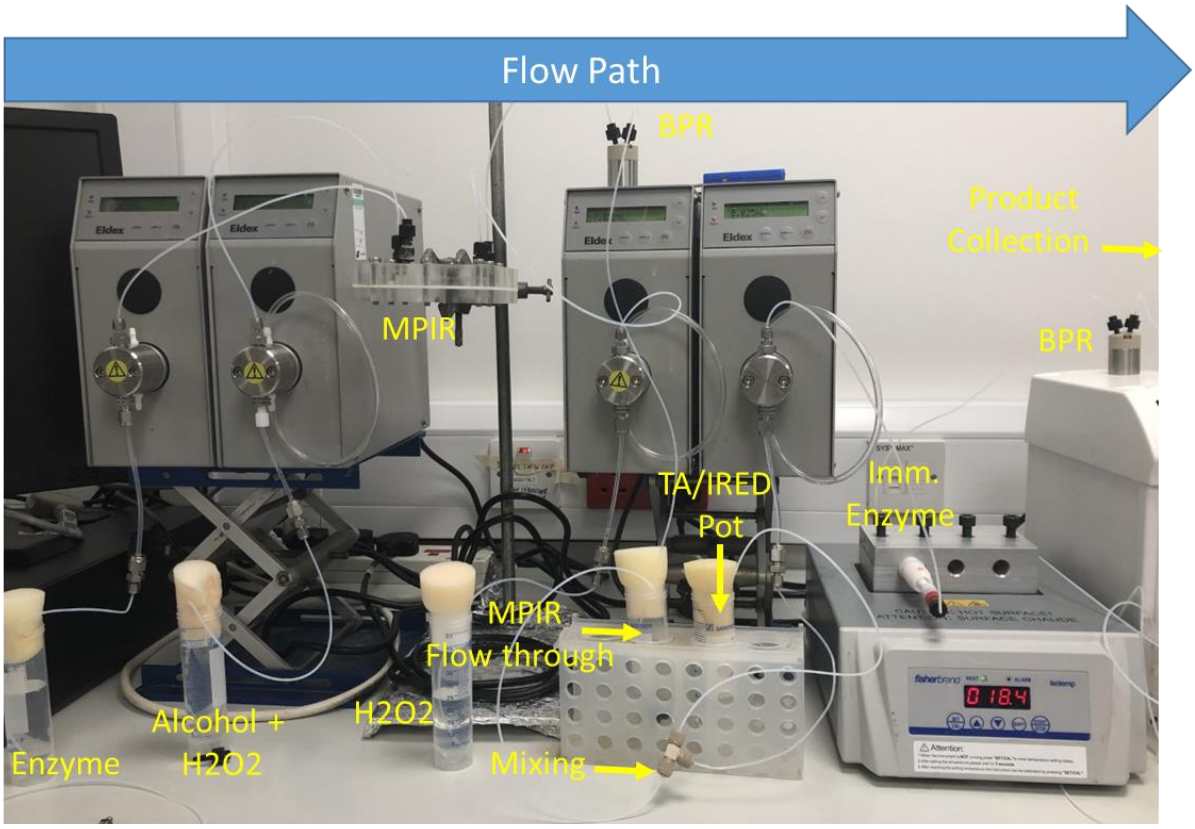

Figure S9: Flow set up for MPIR-packed bed continuous flow reactions.

MPIR-packed bed systems were set up as shown in **Figure 9**. The initial bio-oxidation in the MPIR was run for 90 mins to ensure the system had reached steady state. The flow through was then collected until a reservoir of aldehyde was formed. A Syrris Asia flow chemistry syringe pump with two modules was then used to pump the flow through from the MPIR and an RedAm substrate reservoir (amine (100 mM, 5 eq.), $NADP^+$ (1 mM), glucose (50 mM)). These lines were mixed *via* a Kinesis microstatic mixer and then passed through the immobilized RedAm (Alternatively an Eldex pump with a static mixer pre-fitted can be used). The same approach was adopted when using immobilized transaminases where the TA substrate reservoir (*rac*-Alanine (200 mM), PLP (1 mM)). AcCO6 MPIR reactions were run in KPi buffer (100 mM, pH 7), whilst the RedAm and TA substrate reservoirs were made up in KPi (100 mM, pH 7.5). The GOase-IRED/TA system was run in NaPi buffer (100 mM, pH 7.5). Fractions were basified, extracted into ethyl acetate and analysed by GC-FID.

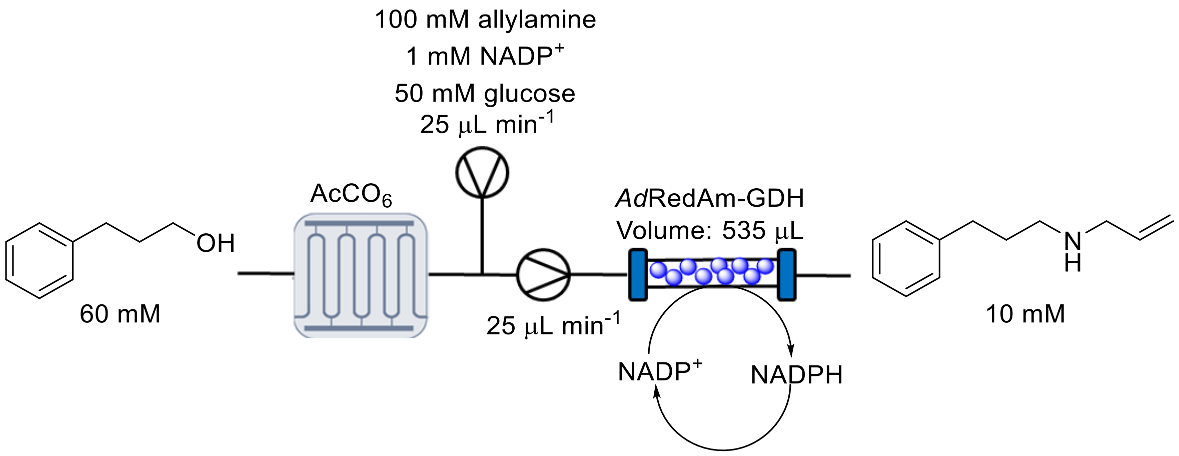

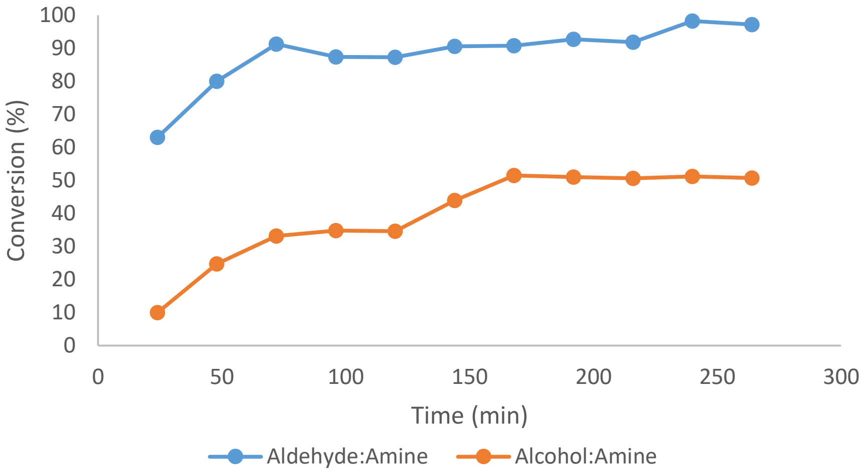

Figure S10: $AcCO_6$-*Ad*RedAm MPIR-packed bed system for the continuous production of hydrocinammylallylamine. Fractions were basified, extracted into ethyl acetate and dried over anhydrous $MgSO_4$ prior to GC-FID analysis. Each data point represents a single analysis.

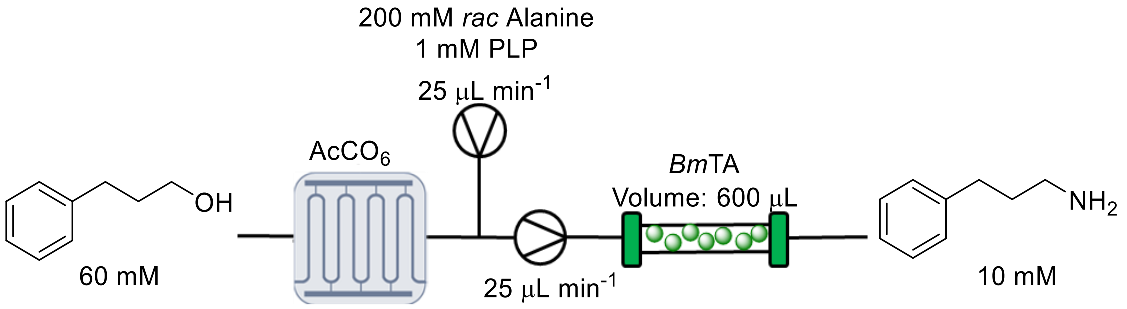

Figure S11: $AcCO_6$-*Bm*TA MPIR-packed bed system for the continuous production of hydrocinammylamine.

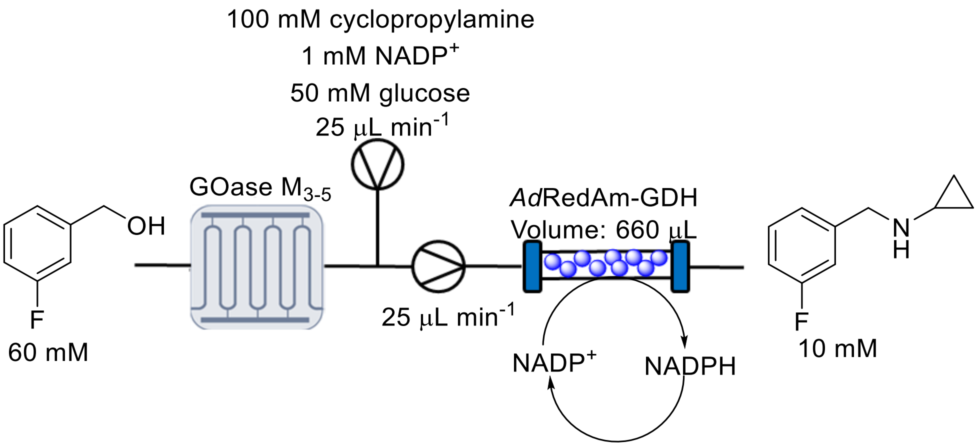

Figure S12: GOase $M_{3\text{-}5}$-*Ad*RedAm MPIR-packed bed system for the continuous production of *N*-(3-fluorobenzyl)cyclopropanamine.

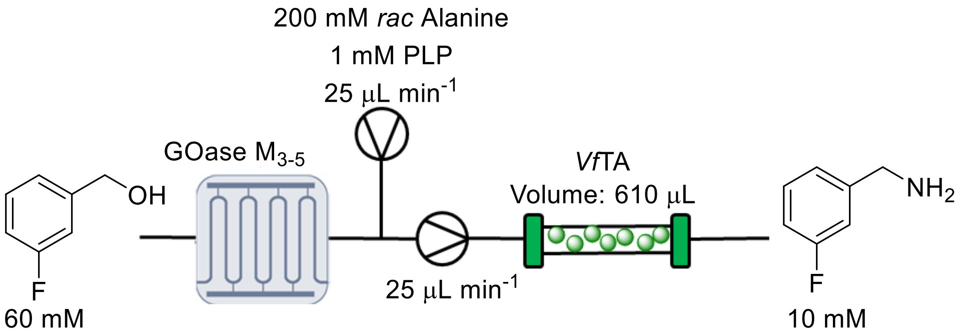

Figure S13: GOase $M_{3\text{-}5}$-*Ad*RedAm MPIR-packed bed system for the continuous production of (3-fluorophenyl)methanamine.

MPIR-Packed bed (with added switching valves) continuous reactions

A.

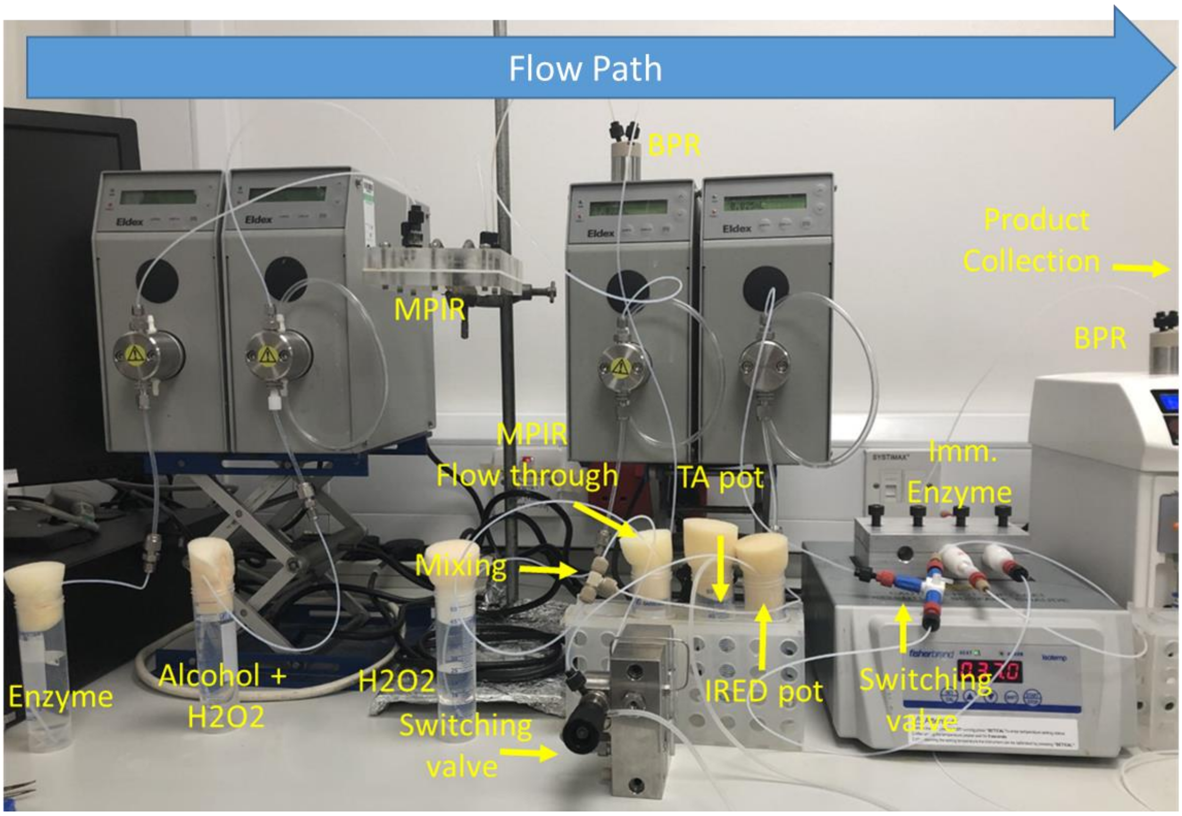

B.

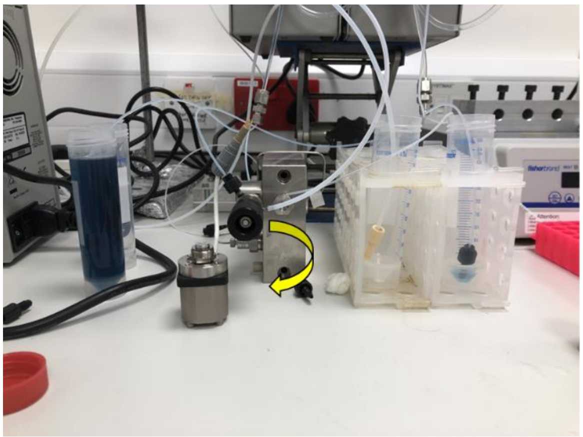

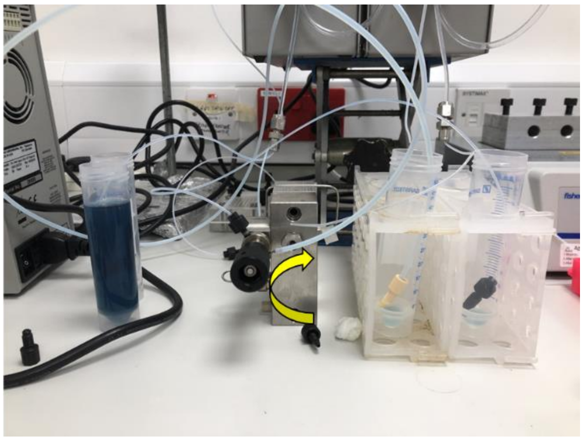

Figure S14: A. Lab set up for MPIR-Packed bed continuous flow reactions with added switching valves to improve efficiency. B. Retrofitted manual HPLC waste valve showing control of flow path.

Screening MPIR-Packed bed systems were set up as pictured above: The initial bio-oxidation in the MPIR was run for 90 minutes to ensure the system had reached steady state, and the effluent discarded. The reaction mixture was collected until a reservoir of aldehyde solution was formed. An Eldex pump (with a pre-fitted static mixer) was then pumped the effluent from the MPIR and a RedAm substrate reservoir (amine substrate (100 mM-5eq.), $NADP^+$ (1 mM), glucose (50 mM)) or a TA substrate reservoir (*rac*-alanine (200 mM), PLP(1 mM)) that were placed before a retrofitted HPLC waste valve. The flow path was controlled by turning the waste valve to pump from either substrate reservoir. The solution was passed through an Omnifit three way switching valve to control the flow path to the immobilized RedAm or TA. GOase-RedAmTA systems were all run in NaPi buffer (100 mM, pH 7.5). Fractions were basified, extracted into ethyl acetate and analysed by GC-FID. Alternatively the HPLC waste valve can be fitted before the MPIR with benzyl alcohol substrate reservoirs that enable choice of alcohol substrate.

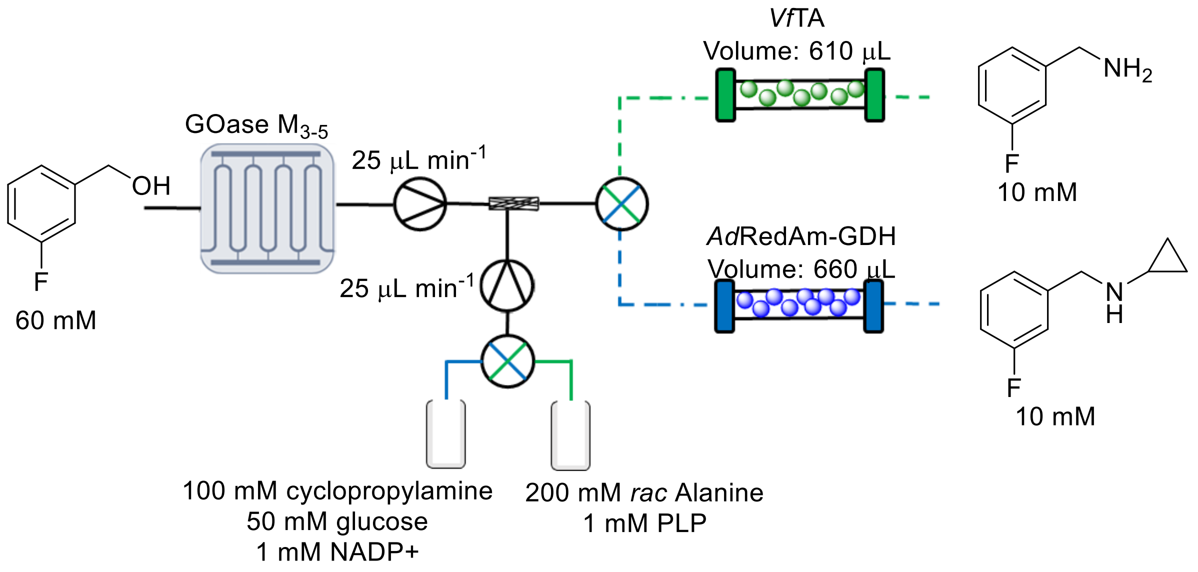

Figure S15: GOase-*Ad*RedAm/*Bm*TA MPIR-packed bed system with the addition of two switching valves allows for change of flow path.

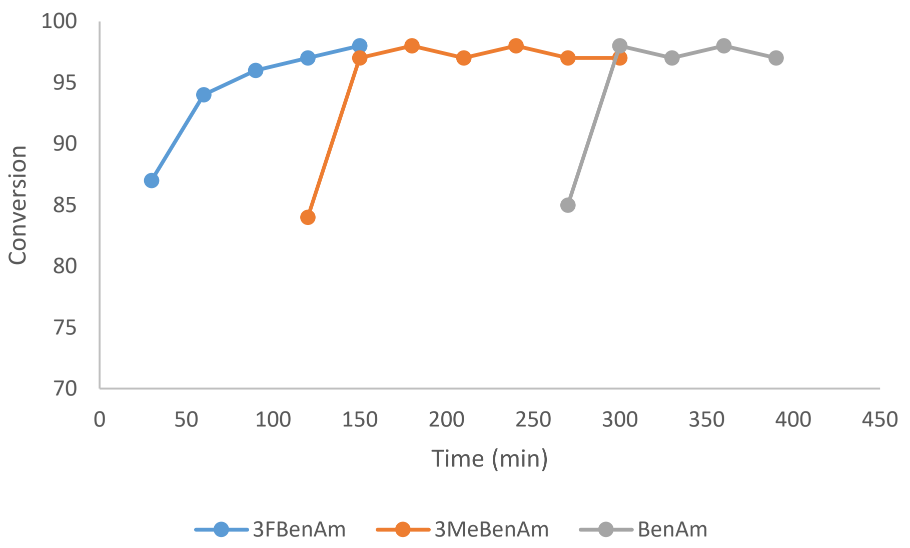

Figure S16: Adding switching valves to the MPIR-packed bed system enabled in flow biocatalyst screening with generation of three substituted benzylamines without intermittent washing steps. Fractions were collected, basified and extracted into ethyl acetate and analysed by GC-FID. Each data point represents a single analysis.

Packed bed- Packed bed continuous reactions

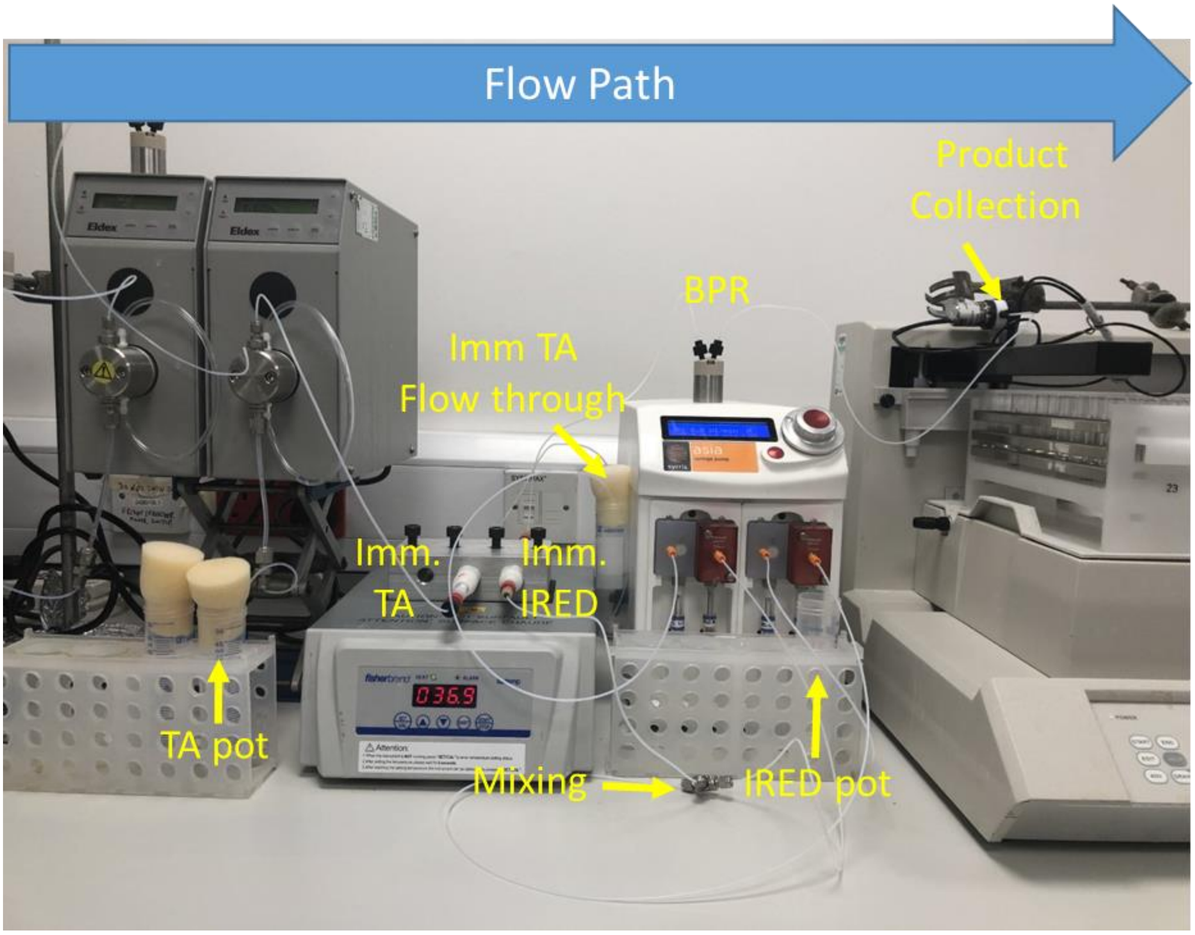

Figure S17: Lab set up for packed bed packed bed continuous flow reactions.

Packed bed-packed bed systems were set up as pictured above: A TA substrate reservoir (aldehyde (20 mM), PLP (1 mM) and *rac*-alanine (200 mM)) was flowed through an immobilized TA. The system was left for 1 hour to reach steady state then the effluent was collected until a reservoir was formed. A Syrris Asia flow chemistry syringe pump with two modules was then used to pump the flow through from the TA module and a RedAm substrate reservoir (carbonyl (10mM), $NADP^+$ (1 mM), glucose (50 mM)). These lines were mixed *via* a Kinesis microstatic mixer or T junction and then passed through the immobilized RedAm to a fraction collector. Fractions were basified, extracted into ethyl acetate and analysed *via* GC-FID.

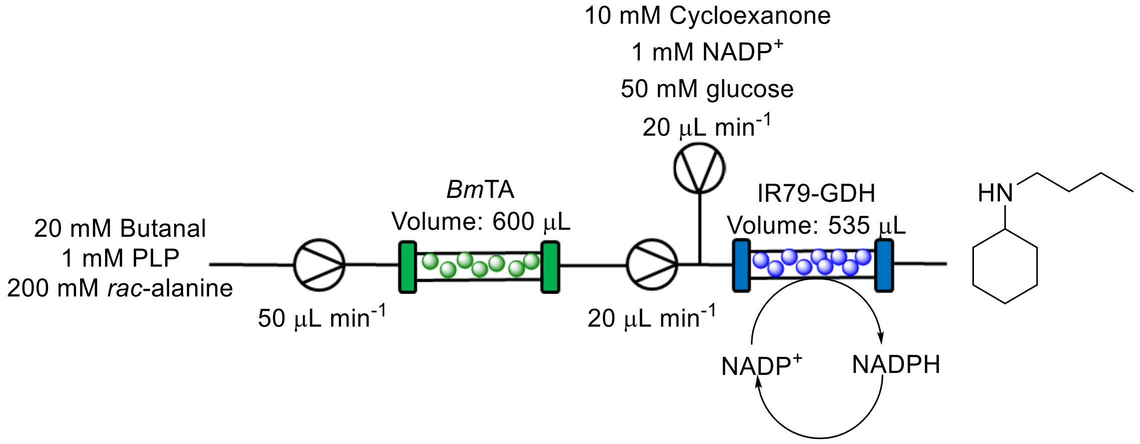

Figure S18: *Bm*TA-IR79 packed bed-packed bed system for the continuous production of *N*-butylcyclohexanamine.

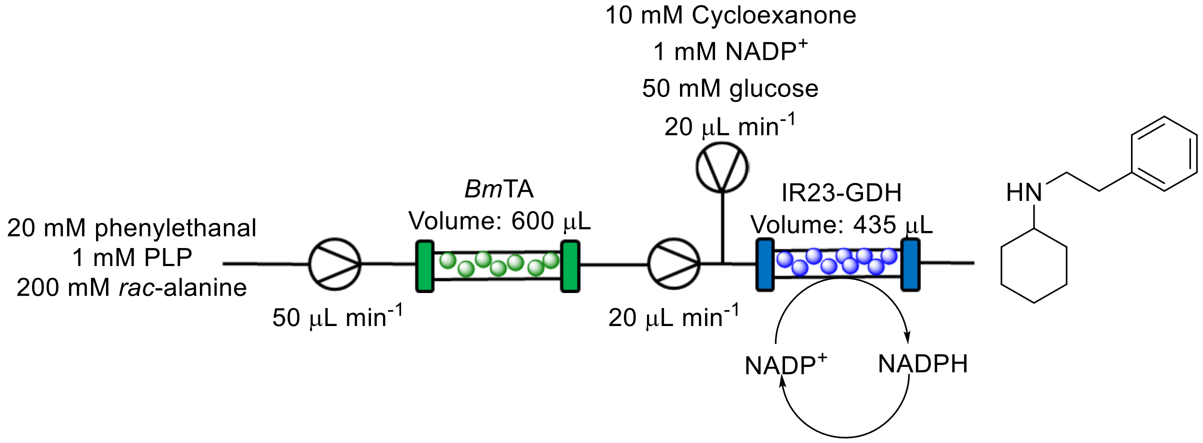

Figure S19: *Bm*TA-IR23 packed bed-packed bed system for the continuous production of *N*-phenylethylcyclohexanamine.

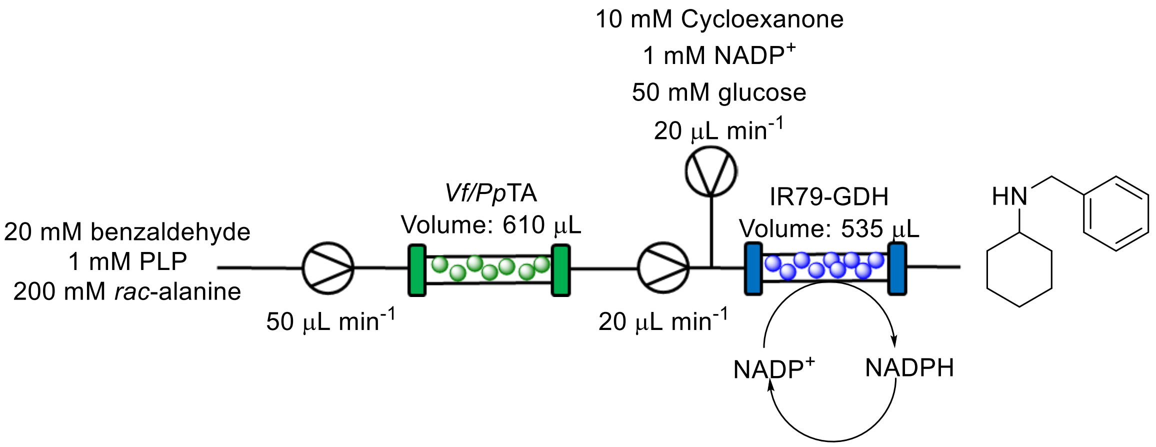

Figure S20: *Vf* or *Pp*TA-IR79 packed bed-packed bed system for the continuous production of *N*-benzylycyclohexanamine.

Packed bed- packed bed (added switching valves) continuous reactions

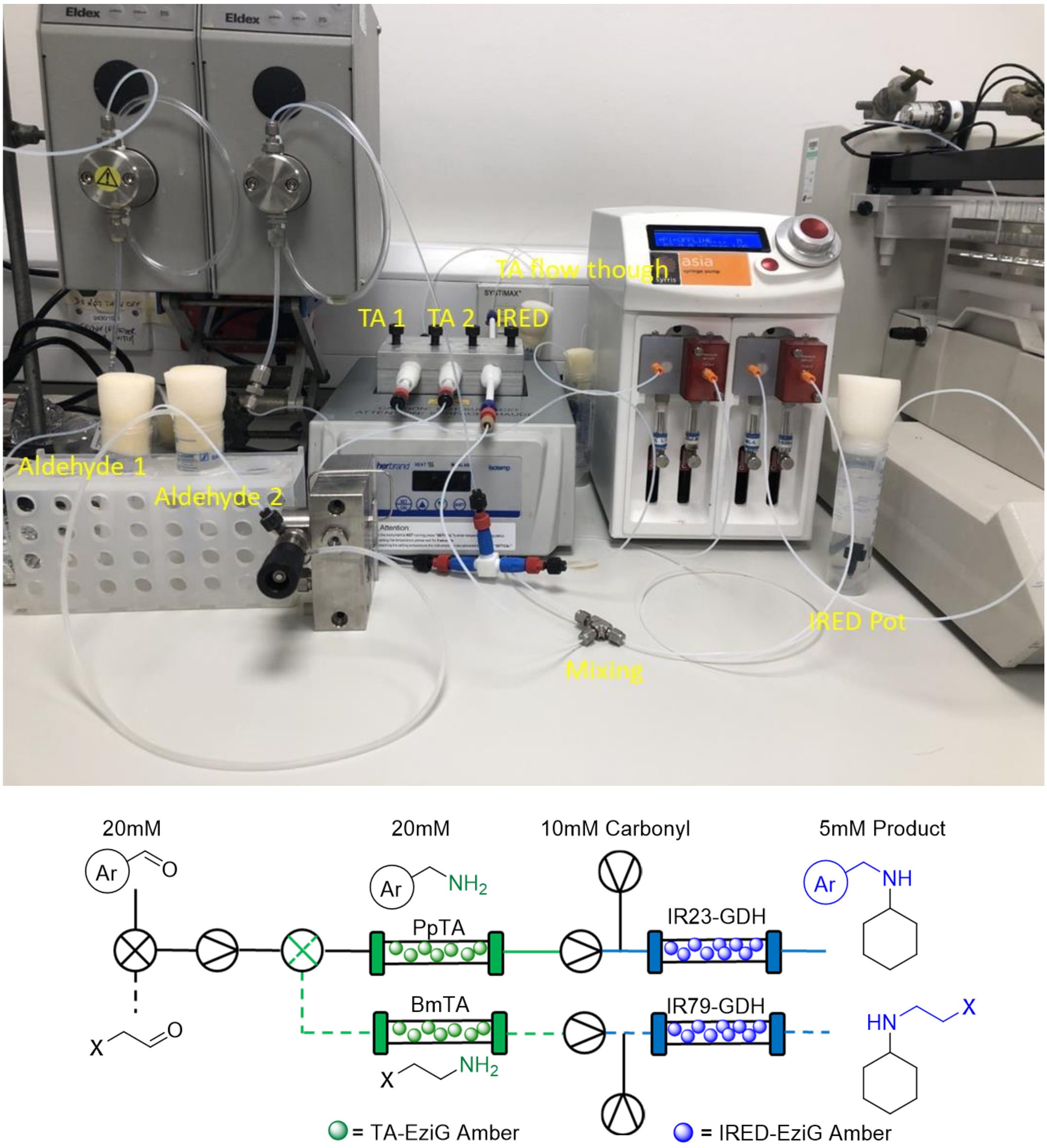

Figure S21: TA-RedAm packed-packed bed system with added switching valves enables rapid change of flow path and efficient synthesis of multiple products in one system.

MPIR-Packed bed- Packed bed continuous reactions

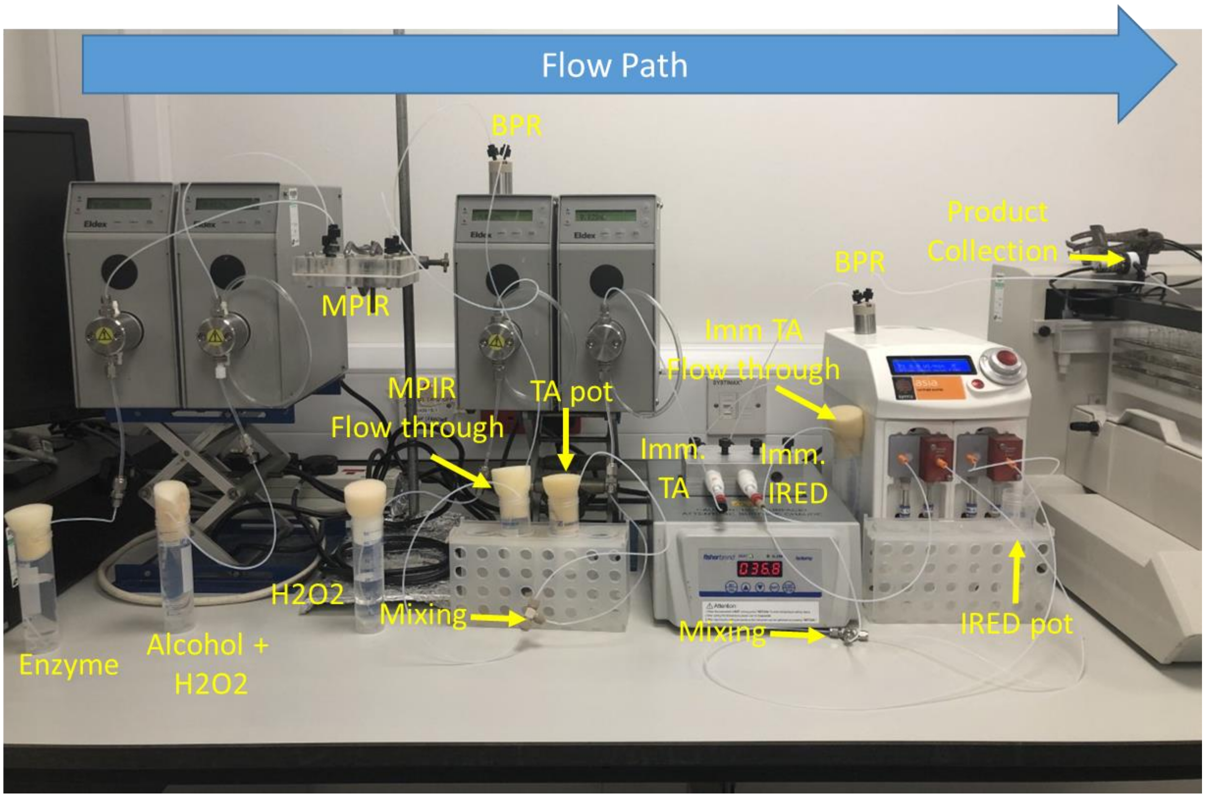

Figure S22: Lab set up for MPIR-packed bed-packed bed continuous flow reactions.

The MPIR and a MPIR-packed bed-packed bed systems were set up as pictured above: The initial bio-oxidation in the MPIR was run for 90 minutes to ensure the system had reached steady state, and the effluent discarded. The reaction mixture was collected until a reservoir of aldehyde solution was formed. A T-junction was fitted before an Eldex pump with a line from the MPIR flow and a line from a TA substrate reservoir (alanine (400 mM), PLP (1 mM)). This was passed through an immobilised transaminase and was run for 60 minutes to ensure steady state was reached. A Syrris Asia flow chemistry syringe pump with two modules was then used to pump the flow through from the TA reaction and a RedAm substrate reservoir (carbonyl (10 mM), $NADP^+$ (1 mM), glucose (50 mM)). These lines were mixed *via* a Kinesis microstatic mixer and passed through the immobilized RedAm. AcCO6 MPIR reactions were run in KPi buffer (100 mM, pH 7), whilst the RedAm and TA substrate reservoirs were made up in KPi buffer (100 mM, pH 7.5). The GOase-RedAm/TA system was run in NaPi buffer (100 mM, pH 7.5). Fractions were basified, extracted into ethyl acetate and analysed by GC-FID.

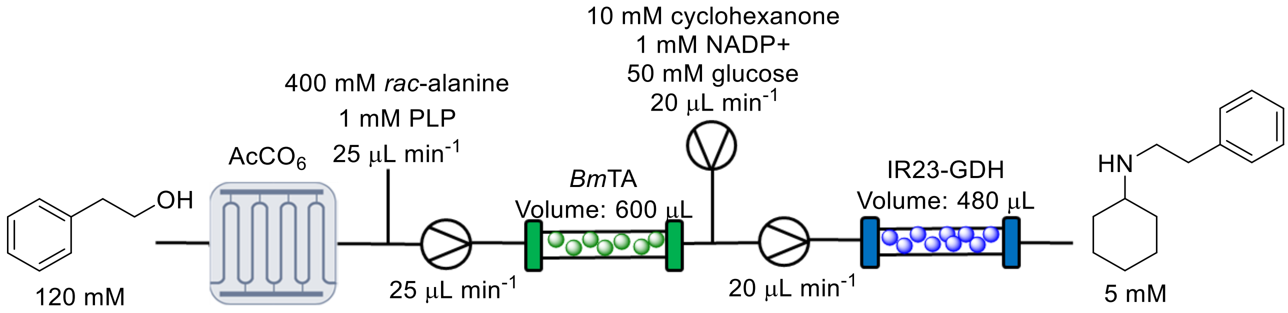

Figure S23: $AcCO_6$-*Bm*TA-IR23 MPIR-packed bed-packed bed system for the continuous production of *N*-phenylethylcyclohexanamine.

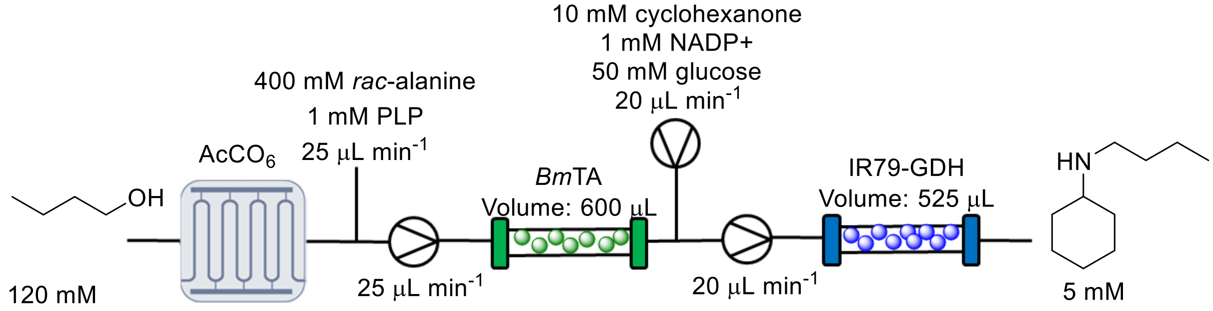

Figure S24: $AcCO_6$-*Bm*TA-IR79 MPIR-packed bed-packed bed system for the continuous production of *N*-butylcyclohexanamine.

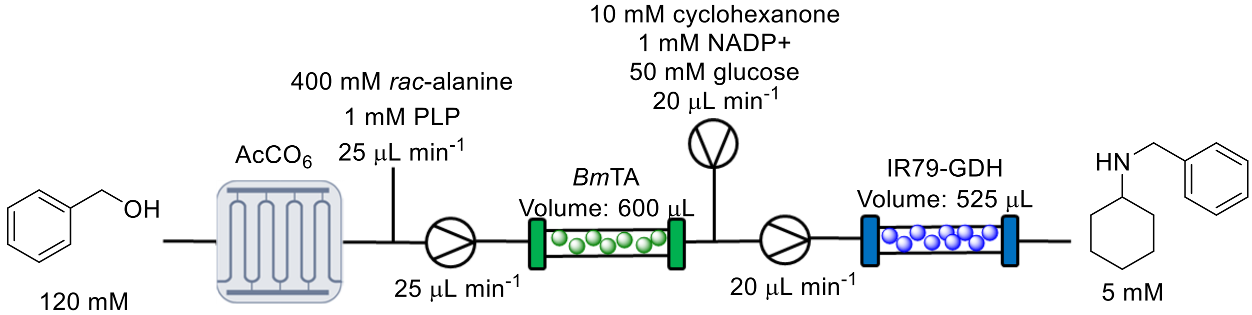

Figure S25: GOase-*Bm*TA-IR79 MPIR-packed bed-packed bed system for the continuous production of *N*-benzylcyclohexanamine.

In flow enzymatic synthesis of 4O-Methylnorbelladine

A previously described high throughput colorimetric screen was used to identify potential RedAms capable of carrying out reductive amination with isovanilin and tyramine to generate 4O-Me norbelladine.[3] Details of the colourimetric screen and what the values represent are given in much greater detail in the following references,[1,3] but a general procedure is as follows:

The assay is based on the principle of running the reaction in the reverse direction as outlined in Scheme 1.0 using an amine of interest as the substrate which is oxidised to the corresponding imine (Figure S26). This is coupled to the production of a red compound (formazan) through a coupled NADPH dependent oxidation with diaphorase. Production of formazan can be monitored at 490 nm to give an indication of activity to coupled RedAm process.

The following protocol was used:

Assay plate

• Each well contains lyophilised IRED lysate, and lyophilised IREDy-to-go components including the co-enzyme $NADP^+$.

• Components which require addition to the plate are INT, amine substrate, and 0.1 M TrisHCl buffer pH 9.0.

Method

1. To generate assay master mix reagent: 25 mL of 0.125 mg.ml-1 INT, and 10 mM amine substrate in 100 mM Tris HCl adjusted to pH 9.0.

2. Using the microplate sealing film cross-hairs as a guide, carefully add 50 µL of the master mix reagent to each well and mix carefully to reconstitute the freeze-dried enzymes. Change tips between additions, to avoid enzyme cross contamination.

3. Upon addition of the master mix reservoir, spin the plate down at 1,500 rpm for 1 minute.

4. Incubate the plate in the dark at ambient temperature or 30 °C for up to 24 hours. Although a red colorimetric change can be observed within 10 minutes upon substrate addition. Wells B02, D08, F12, H21, I02, I07, K16, O01, O08 and P17 can be regarded as false positives.

5. The colourimetric intensity was quantified by an absorbance spectrometer and the values in the table represent the absorbance at 490 nm for the production of formazan.

6. A blank plate was also run under the same conditions and the background absorbance values deducted from the final absorbance values obtained from the screen with the desired substrate to eliminate false positives.

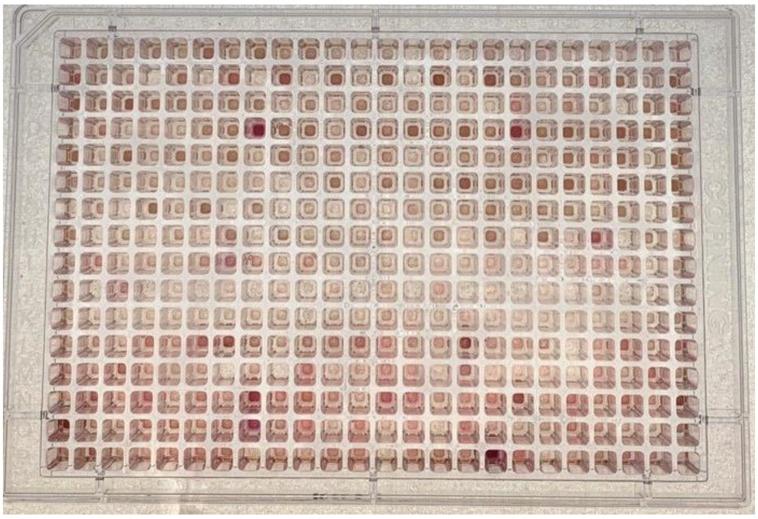

	1	2	3	4	5	6	7	8	9	10	11	12	13	14	15	16	17	18	19	20	21	22	23	24
A	1.1	1.6	1.1	1.5	1.1	1.8	1.1	1.8	1.1	1.6	0.9	0.6	1.0	0.6	1.0	0.7	1.1	0.5	0.8	0.9	0.7	0.7	0.7	0.8
B	1.7	1.2	1.4	0.5	1.6	0.7	1.9	0.5	2.0	0.6	2.1	1.0	2.0	0.5	2.3	1.0	2.3	1.7	1.1	1.4	1.3	1.9	0.7	2.3
C	2.2	1.8	2.3	1.8	1.8	1.5	1.6	1.6	0.9	1.3	0.8	1.4	0.9	1.4	1.2	1.1	0.8	1.1	0.9	1.2	1.2	0.7	1.9	1.4
D	0.9	1.5	0.8	0.9	0.9	1.5	1.7	2.9	2.0	1.7	0.9	1.9	1.6	2.1	1.6	2.2	1.6	2.6	1.8	2.2	1.7	2.3	2.0	1.8
E	2.0	0.9	2.1	1.0	1.8	1.1	2.0	0.8	1.8	1.0	1.2	0.9	1.3	0.8	1.0	1.4	1.3	1.6	0.9	1.8	1.7	2.0	1.1	2.3
F	1.2	1.0	1.1	1.2	0.9	0.7	0.8	0.8	0.5	0.7	1.7	0.8	1.6	0.9	1.7	2.1	1.7	1.7	1.7	2.2	1.3	2.6	1.8	2.4
G	0.5	1.2	0.4	2.0	0.9	1.9	0.5	1.4	0.5	1.0	0.6	0.9	0.6	1.0	1.0	1.3	1.5	1.2	1.5	1.6	1.1	1.7	0.6	1.6
H	1.2	0.6	1.3	0.6	1.2	0.6	1.4	0.6	1.3	0.8	1.1	0.8	1.3	0.7	1.3	0.6	1.5	0.4	1.6	0.7	1.9	0.5	1.3	0.8
I	0.8	1.2	0.7	0.9	0.9	1.1	1.2	1.1	0.8	1.0	0.8	1.0	0.8	0.9	0.6	0.8	0.6	0.6	0.6	0.7	0.8	0.8	0.7	1.0
J	0.7	0.6	0.9	0.7	0.5	0.6	0.6	0.6	0.6	0.7	0.6	0.6	0.7	0.6	0.6	0.5	0.5	0.4	0.5	0.4	0.8	0.5	0.7	0.8
K	0.8	0.8	0.8	0.8	0.8	0.8	0.9	0.8	0.8	0.6	0.8	0.7	0.8	0.6	0.6	0.6	0.6	0.5	0.6	0.6	0.6	0.8	0.9	1.2
L	1.0	1.1	0.8	1.3	1.0	1.4	1.9	1.3	0.9	1.1	1.0	1.1	0.9	0.9	0.8	1.7	0.9	0.8	1.0	0.5	0.9	1.3	1.1	1.3
M	0.9	1.0	1.3	0.9	0.8	0.8	0.4	0.7	0.4	0.9	0.5	0.6	0.7	0.7	0.5	1.2	0.5	0.5	0.7	0.5	0.8	1.0	1.2	1.2
N	0.9	1.3	1.0	1.1	0.8	1.0	0.8	1.8	1.0	1.1	0.9	0.7	1.2	1.0	0.8	0.7	0.8	1.9	0.8	1.0	1.0	1.2	1.0	2.0
O	2.9	0.9	0.9	1.1	1.2	1.0	1.0	1.5	1.0	0.8	0.7	0.9	0.7	0.7	0.6	0.9	0.8	0.7	0.9	1.0	1.0	1.0	0.9	1.5
P	1.2	1.6	0.9	0.8	0.9	0.9	1.0	0.6	0.8	0.6	0.9	0.9	0.5	1.0	0.9	0.9	2.8	1.0	0.5	0.8	1.0	1.1	1.0	0.6

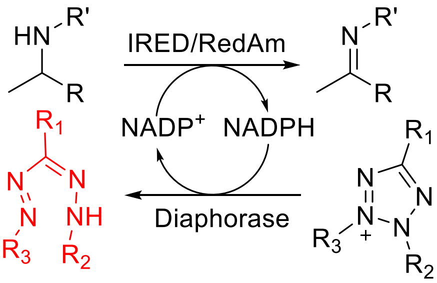

Figure S26: Top: picture of IREDy-2-go screen after 24 hours incubation. Bottom: absorbance recorded from plates with IR-80 highlighted in blue. Diaphorase reaction coupled to RedAm oxidation shown for the production of the red formazan dye.

4-OMe norbelladine batch reaction

Analyitical scale (500 µL) IR-80 reactions were carried out in NaPi buffer (100 mM, pH 7.5, 20% v/v DMSO) and contained: IR-80 (10 mg mL^{-1}, lysate), isovanillin (5 mM), tyramine (25 mM), $NADP^+$ (0.5 mM), glucose (50 mM) and *Bs*GDH (1 mg mL^{-1}). The reaction was incubated at 30 °C overnight with 200 rpm shaking. Biotransformations were filtered using 10kDa MWCO Vivapsin column. The filtrate was removed and the residue suspended in H_2O and filtered again. The remaining residue was then suspended in MeOH/DMSO, filtered and then analysed by MALDI-ToF.

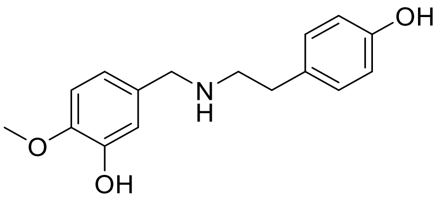

Exact Mass: 273.14

$[M+H^+]$ 274.14

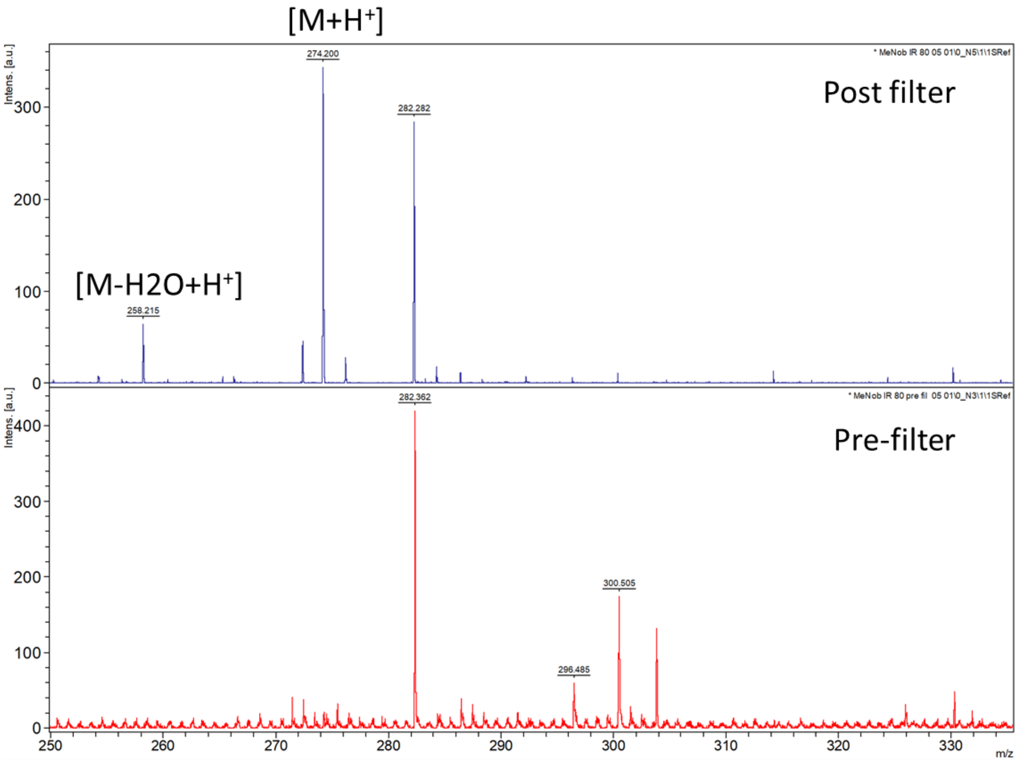

Figure S27: Top: MALDI-ToF analysis of IR-80 reductive amination of isovanillin with tyramine. Bottom: Filtered biotransformation with the residue re-suspended in DMSO

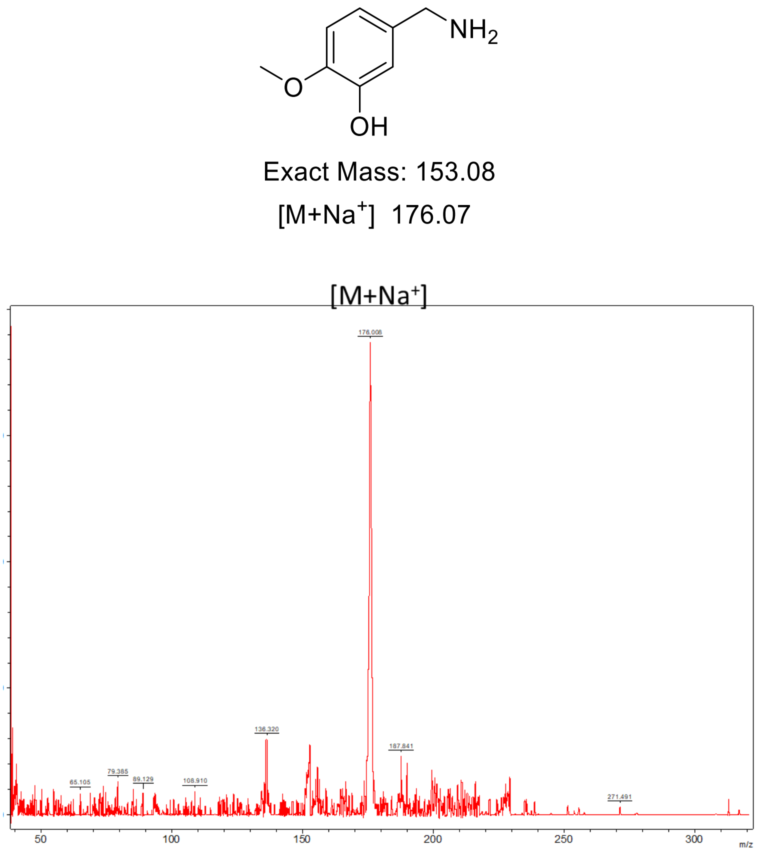

Figure S28: MS2 analysis of m/z 274.2 showing fragmentation of 4-OMethyl norbelladine

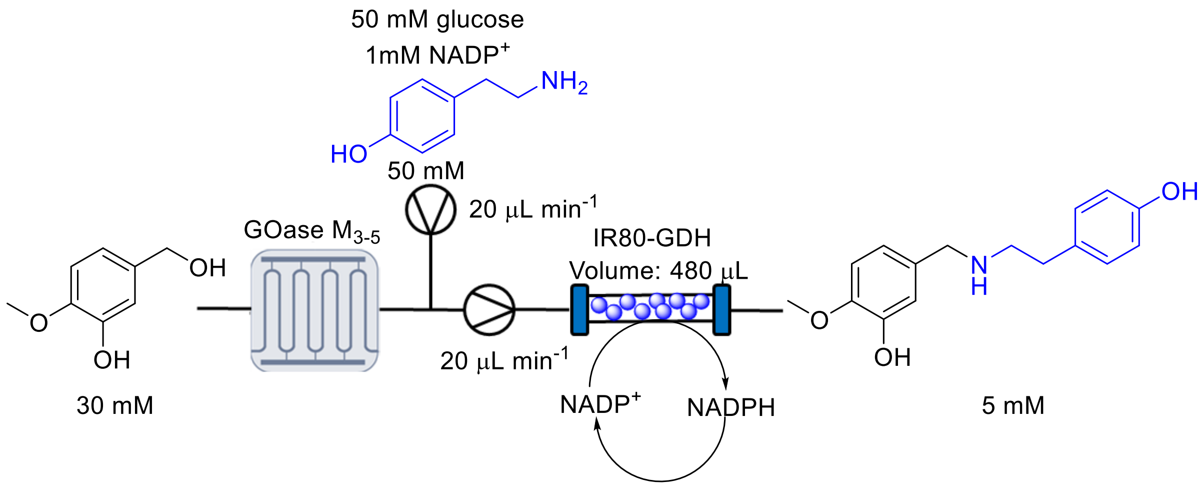

Figure S29: GOase-IR80 MPIR-packed bed system for the continuous production of 4-OMe norbelladine.

MPIR-Packed bed systems were set up as pictured above: The initial bio-oxidation of isovanillnyl alcohol (30 mM) in the MPIR (GOase M_{3-5} (5 mg mL^{-1}, lysate)) was run for 90 minutes to ensure the system had reached steady state, and the effluent discarded. The reaction mixture was collected until a reservoir of aldehyde solution was formed. A Syrris Asia flow chemistry syringe pump with two modules was then used to pump the flow through from the MPIR and a RedAm substrate reservoir (tyramine (50 mM), $NADP^+$ (1 mM), glucose (50 mM)). The effluent was filtered using a 10KDa MWCO Vivaspin column, the filtrate was removed and the residue re-suspended in H_2O and filtered again. The remaining residue was then re-suspended in MeOH/DMSO and filtered. An aliquot of the filtrate was taken for MS analysis with the remaining volume being dried under reduced pressure.

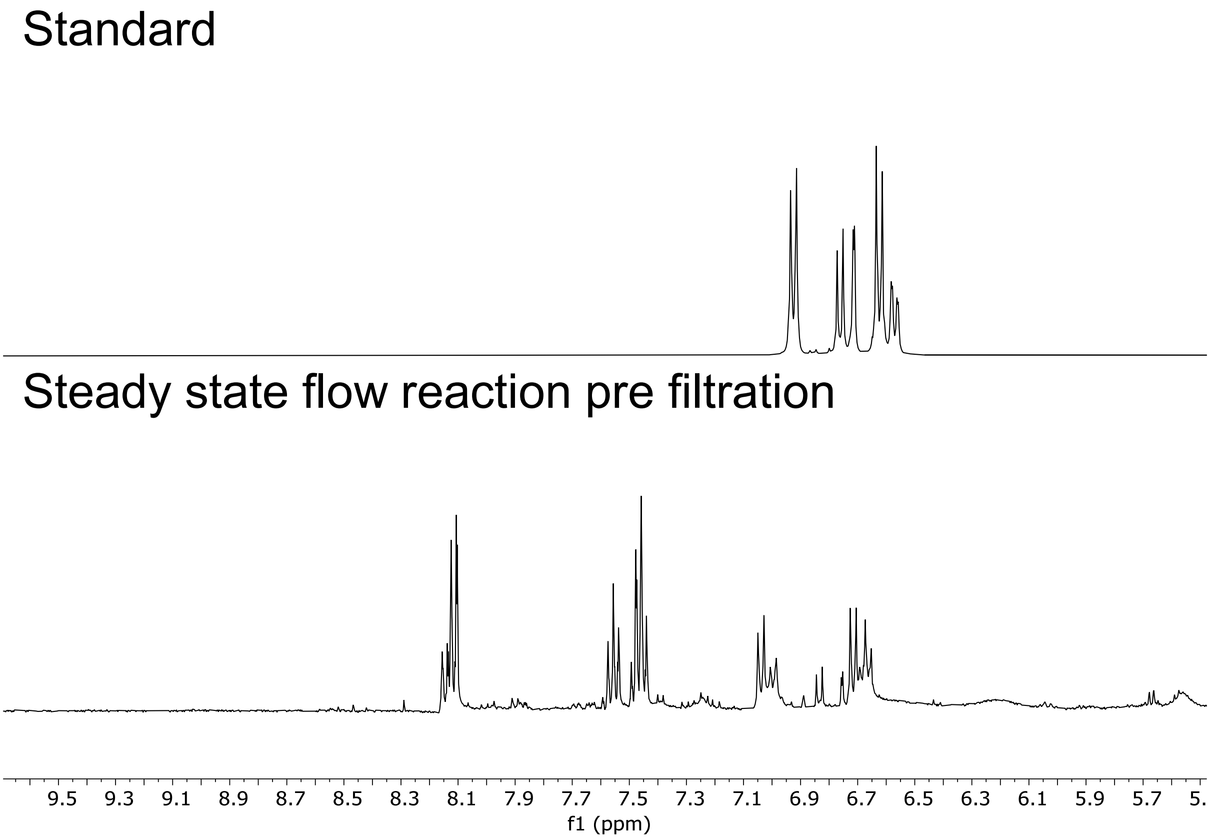
Standard
Steady state flow reaction pre filtration
9.5 9.3 9.1 8.9 8.7 8.5 8.3 8.1 7.9 7.7 7.5 7.3 7.1 6.9 6.7 6.5 6.3 6.1 5.9 5.7 5.
f1 (ppm)

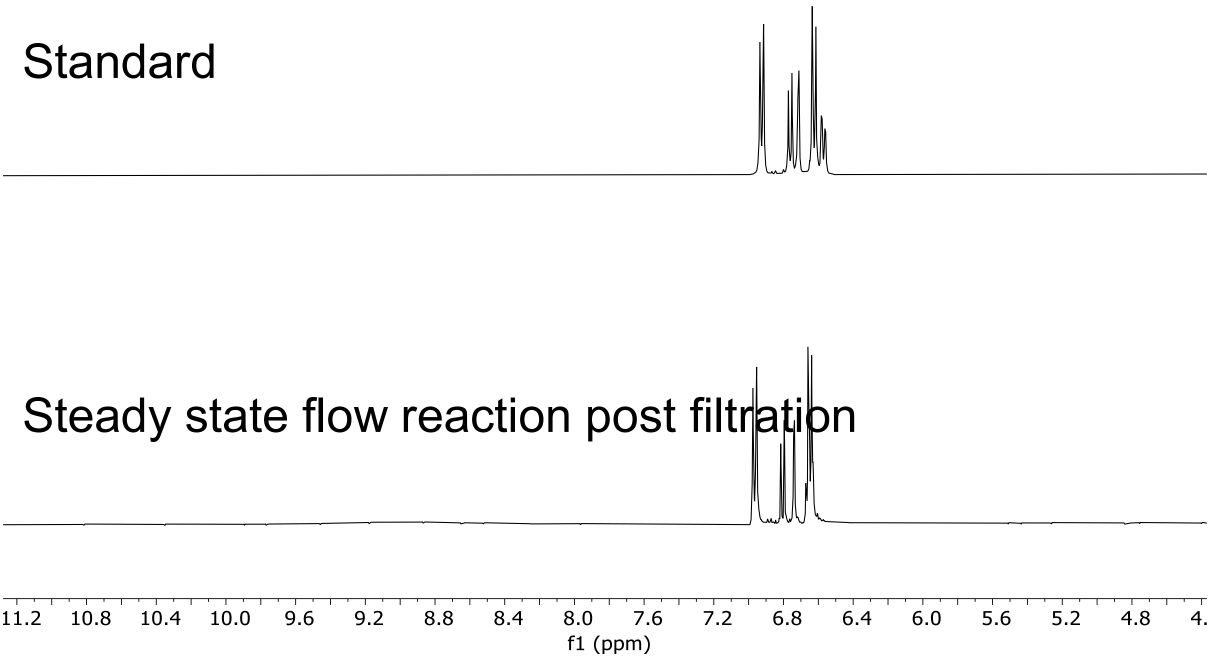
Standard
Steady state flow reaction post filtration
11.2 10.8 10.4 10.0 9.6 9.2 8.8 8.4 8.0 7.6 7.2 6.8 6.4 6.0 5.6 5.2 4.8 4.
f1 (ppm)

Figure S30: ^{1}H NMR spectra showing standard of 4-OMe norbelladine compared the flow reaction at steady state before and after filtration of the insoluble residue.

GC chromatograms

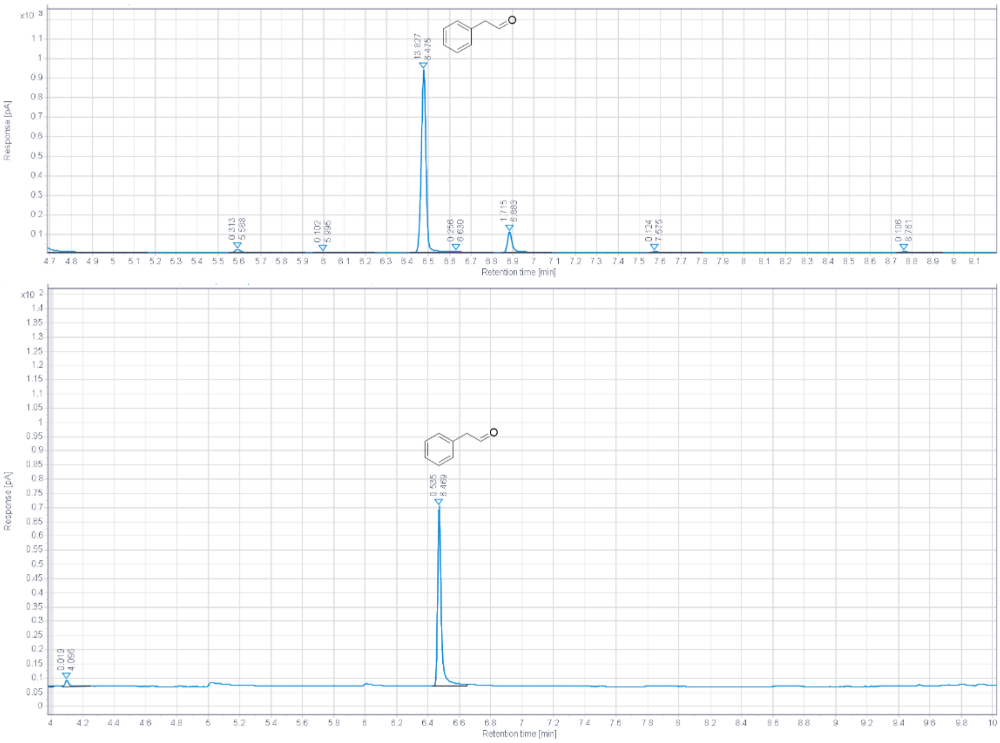

Figure S31: GC-FID spectra of: Top: phenylethanal standard; Bottom: steady state fraction from MPIR biooxdiation of phenylethanol with $AcCO_6$.

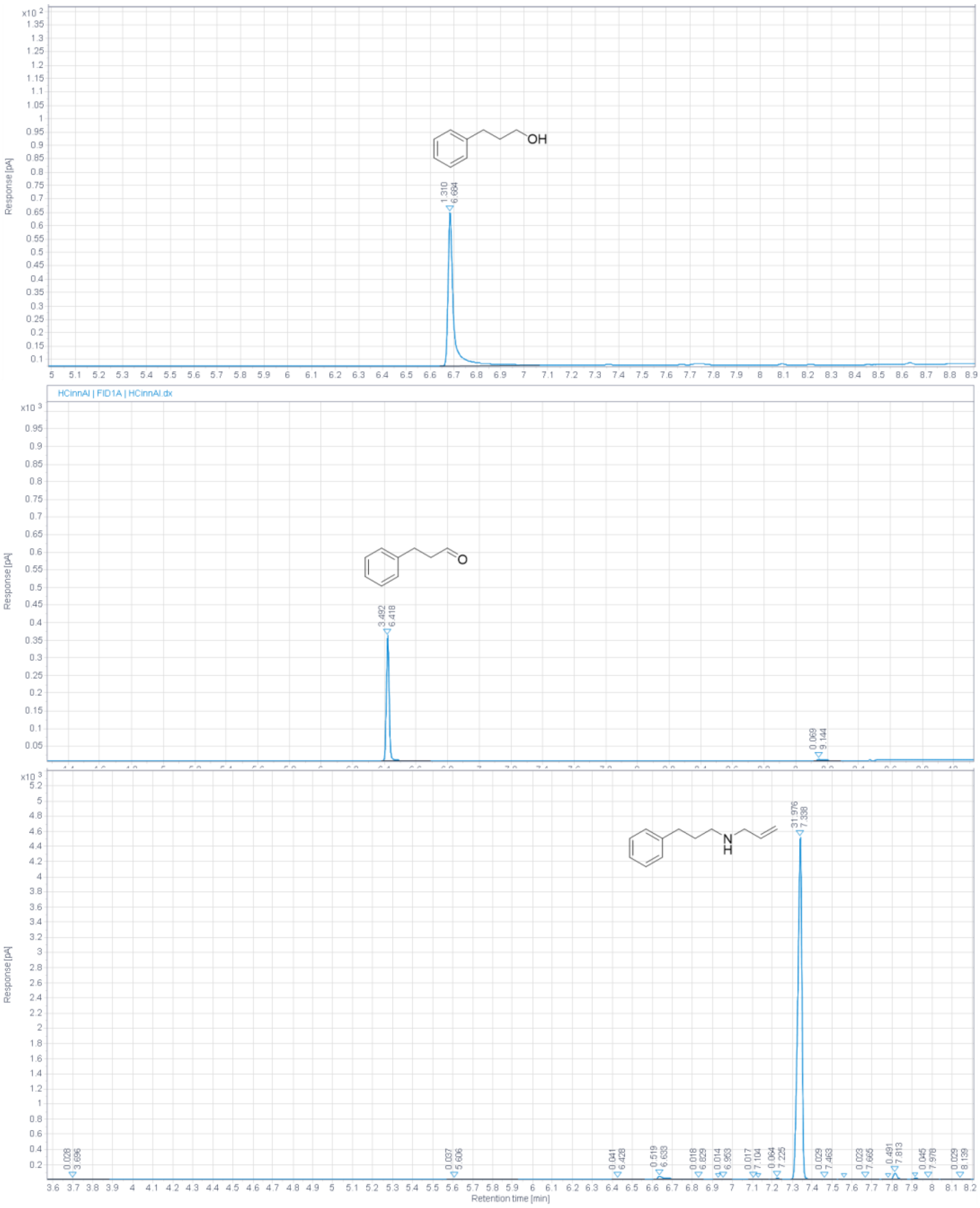

Figure S32: GC-FID spectra of analytical standards: Top: hyrdocinnamylalcohol; Middle: hydrocinnamaldehyde; Bottom: *N*-Hyrdocinnamylallyl amine.

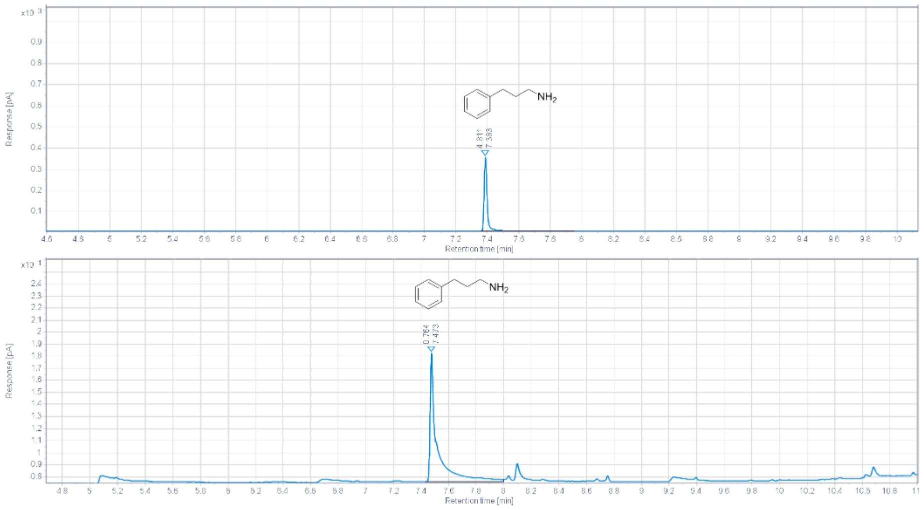

Figure S33: GC-FID spectra of: Top: hydrocinammylamine analytical standard; Bottom: steady state of AcCO6-*Bm*TA MPIR-packed bed flow reaction.

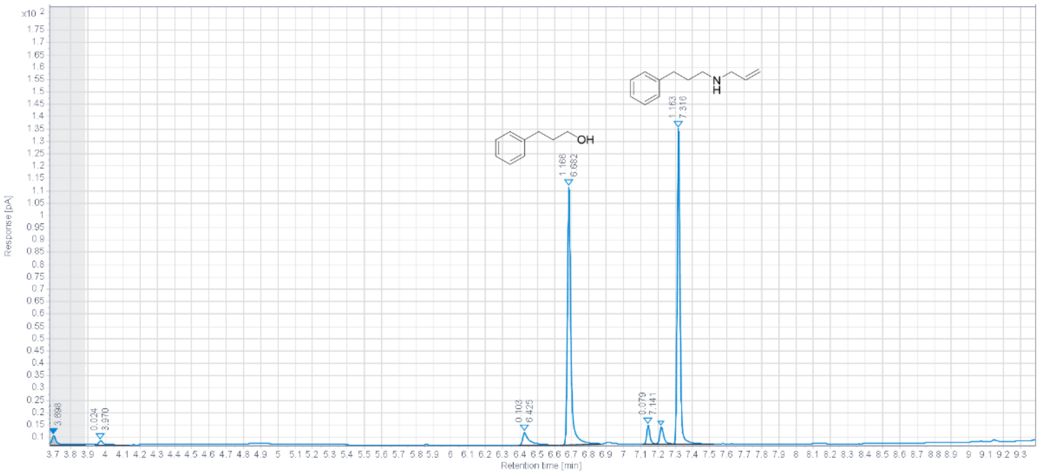

Figure S34: GC-FID spectra of AcCO6-*Ad*RedAm MPIR-packed bed flow reaction.

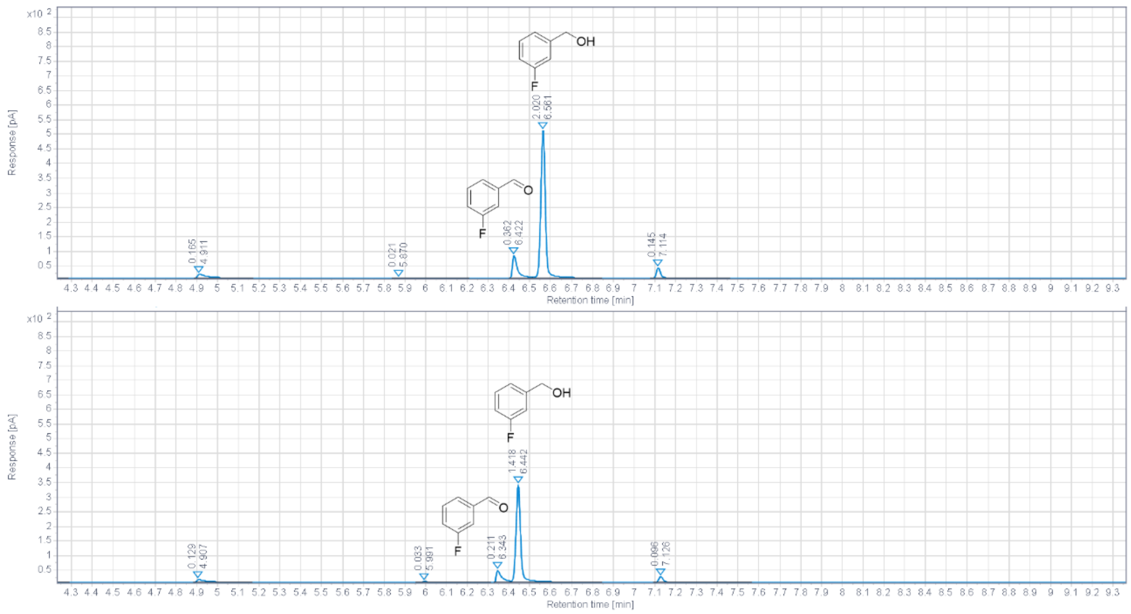

Figure S35: GC-FID spectra of: Top: batch one pot GOase-*Vf*TA with 3-fluoro benzyl alcohol; Bottom: GOase-*Ad*RedAm reaction with 3-fluoro benzyl alcohol.

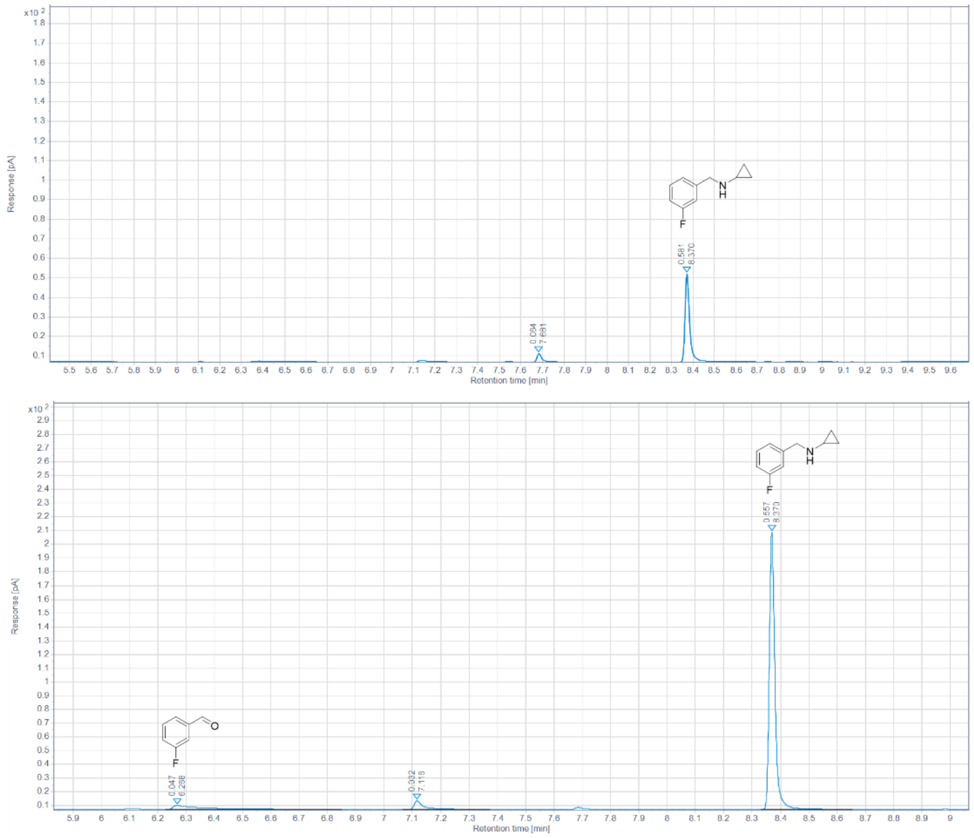

Figure S36: GC-FID spectra of: Top: analytical standard of *N*-(3-fluorobenzyl)cyclopropanamine; Bottom: steady state fractions from GOase-*Ad*RedAm MPIR-packed bed system.

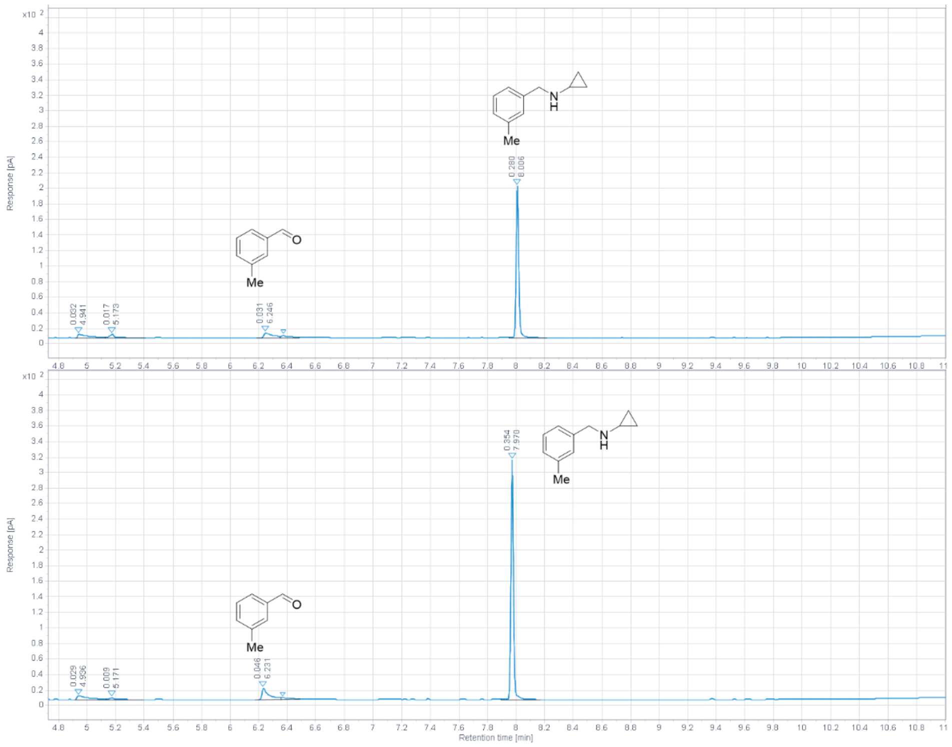

Figure S37: GC-FID spectra of: Top: analytical standard of *N*-(3-methylbenzyl)cyclopropanamine; Bottom: steady state fractions from GOase-*Ad*RedAm MPIR-packed bed system.

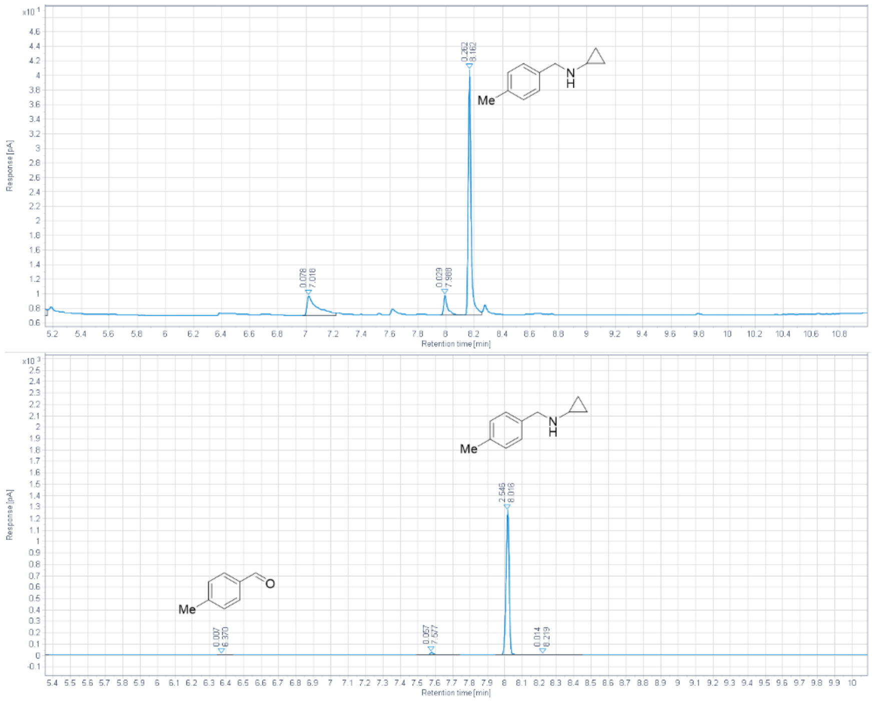

Figure S38: GC-FID spectra of: Top: analytical standard of *N*-(4-methylbenzyl)cyclopropanamine; Bottom: steady state fractions from GOase-*Ad*RedAm MPIR-packed bed system.

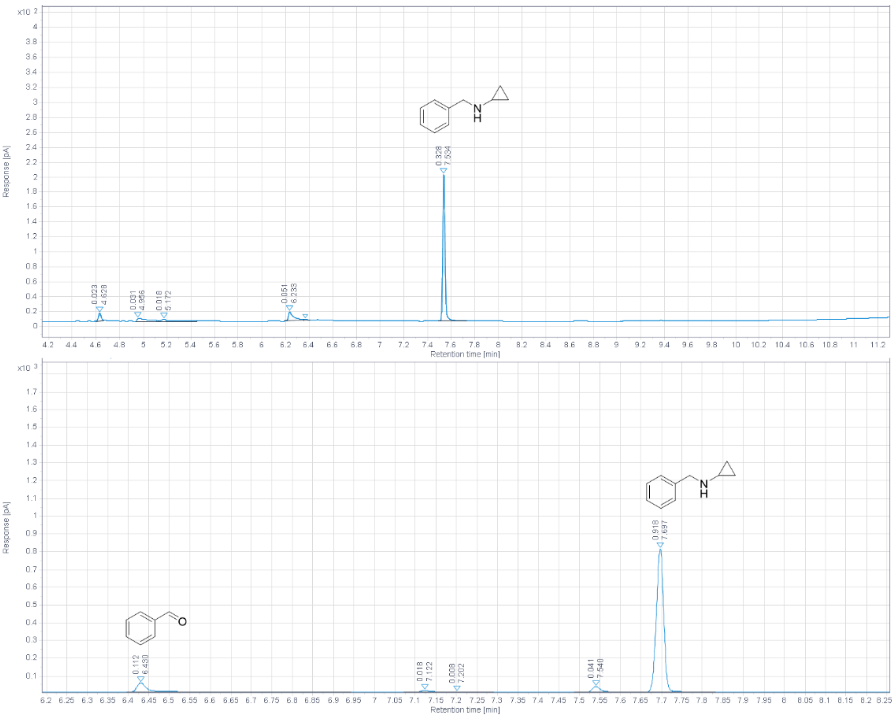

Figure S39: GC-FID spectra of: Top: analytical standard of *N*-benzylcyclopropanamine; Bottom: steady state fractions from GOase-*Ad*RedAm MPIR-packed bed system.

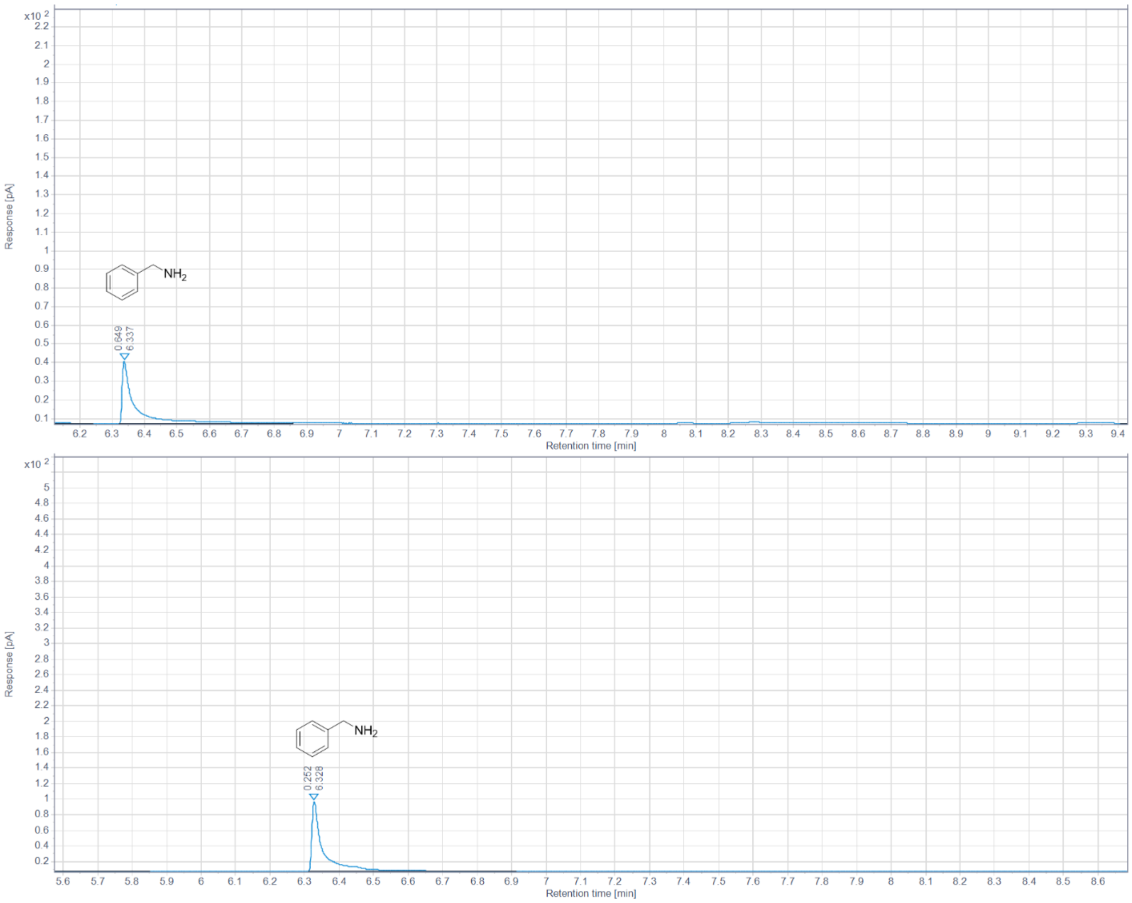

Figure S40: GC-FID spectra of: Top: analytical standard of benzylamine; Bottom: steady state of GOase-*Vf*TA MPIR-Packed bed system.

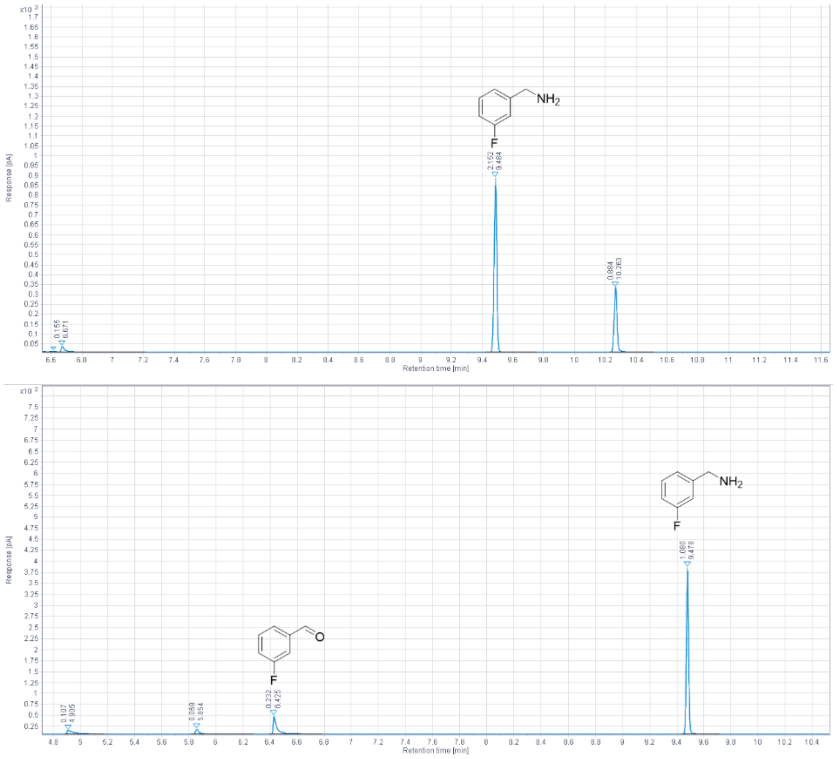

Figure S41: GC-FID spectra of: Top: analytical standard 3-Fluorobenzylamine; Bottom: steady state of GOase-*Vf*TA MPIR-Packed bed flow reaction.

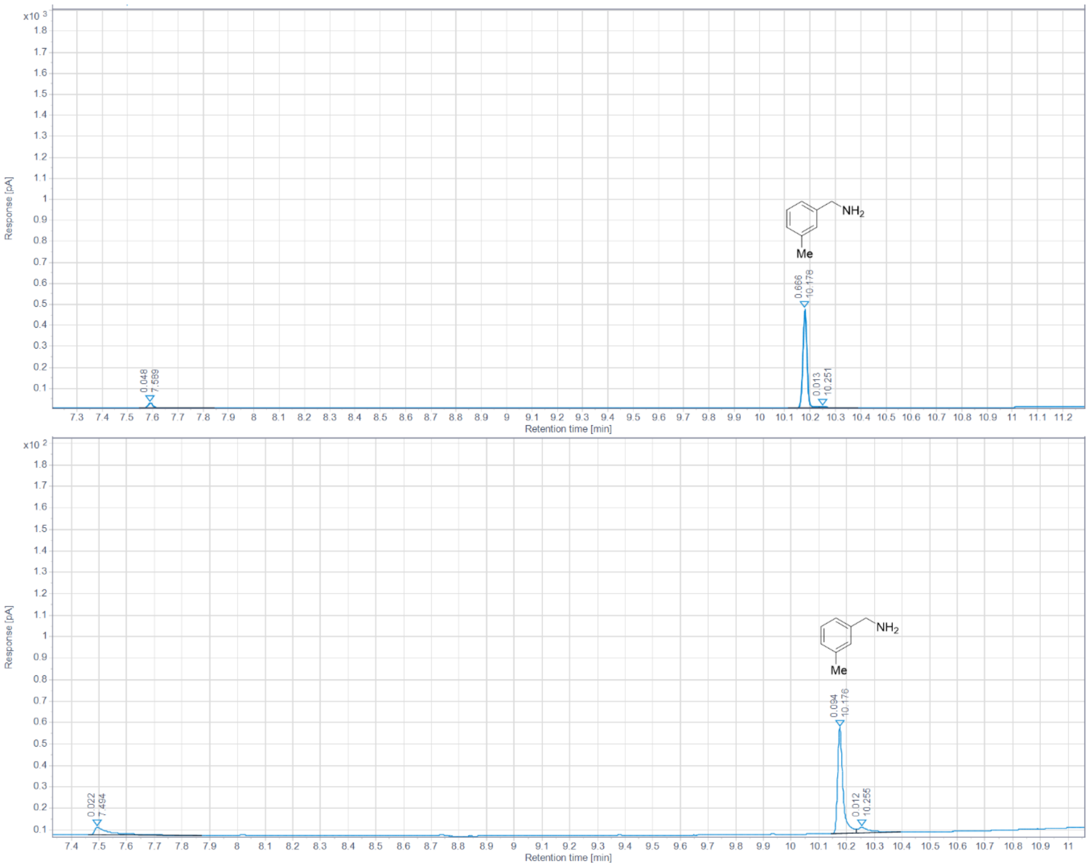

Figure S42: GC-FID spectra of: Top: analytical standard 3-Methylbenzylamine; Bottom: steady state of GOase-*Vf*TA MPIR-Packed bed flow reaction.

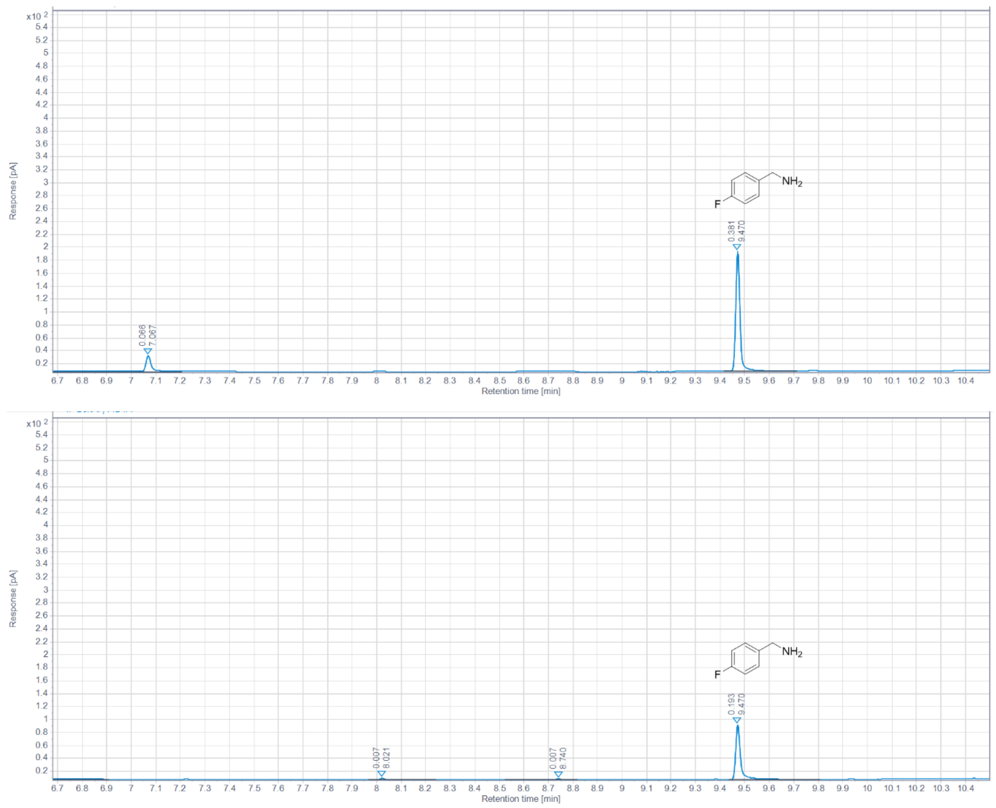

Figure S43: GC-FID spectra of: Top: analytical standard 4-fluorobenzylamine; Bottom: steady state of GOase-*Vf*TA MPIR-Packed bed flow reaction.

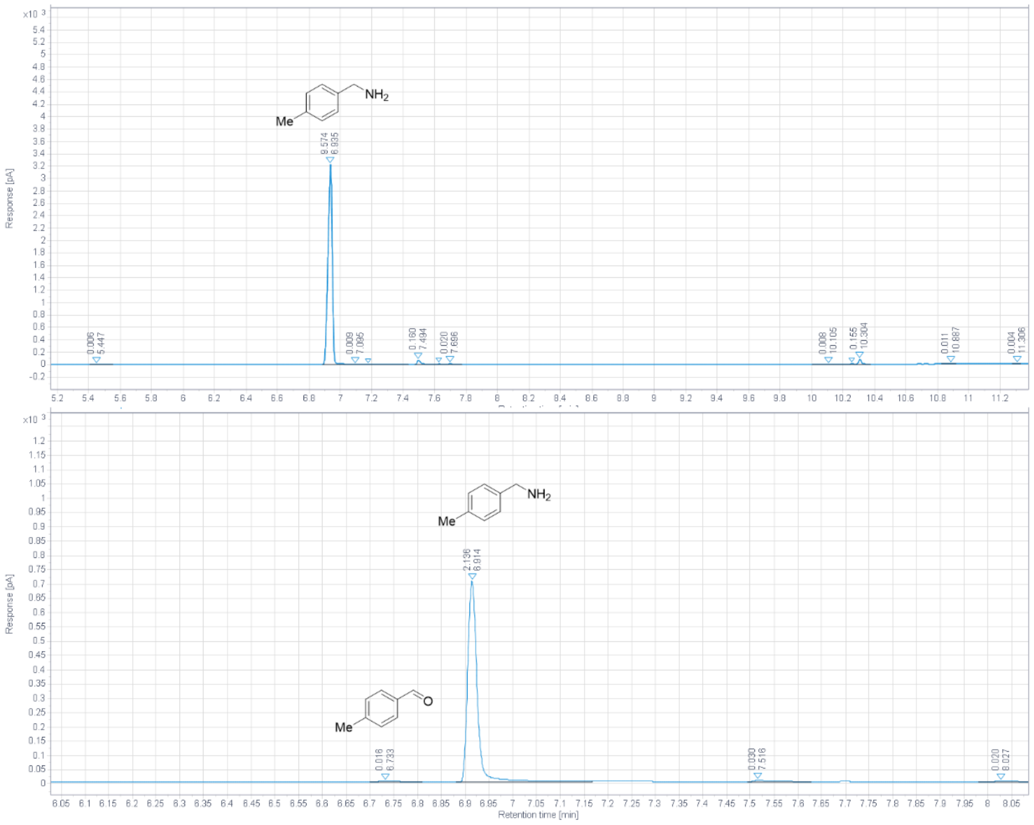

Figure S44: GC-FID spectra of: Top: analytical standard 4-Methylbenzylamine; Bottom: steady state of GOase-*Vf*TA MPIR-Packed bed flow reaction.

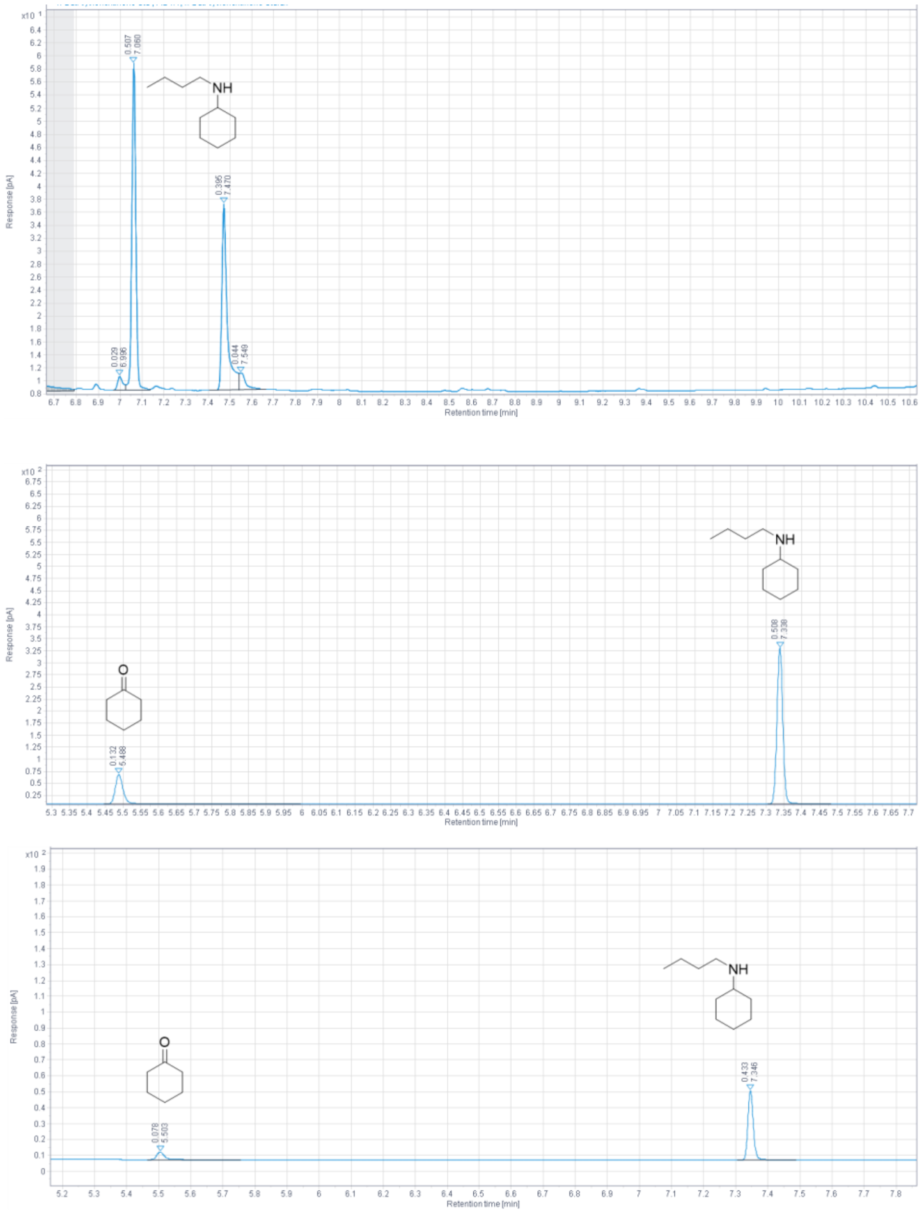

Figure S45: GC-FID spectra of: Top: analytical standard *N*-butylcyclohexanamine; Middle: steady state of IR-79 single packed bed flow reaction; Bottom: steady state of *Bm*TA-IR79 packed bed packed bed flow reaction.

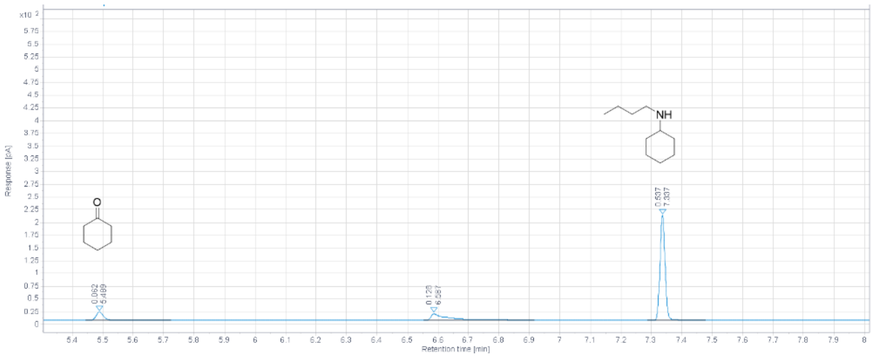

Figure S46: GC-FID spectra of steady state fractions from the $AcCO_6$-*Bm*TA-IR79 MPIR-packed bed-packed bed system.

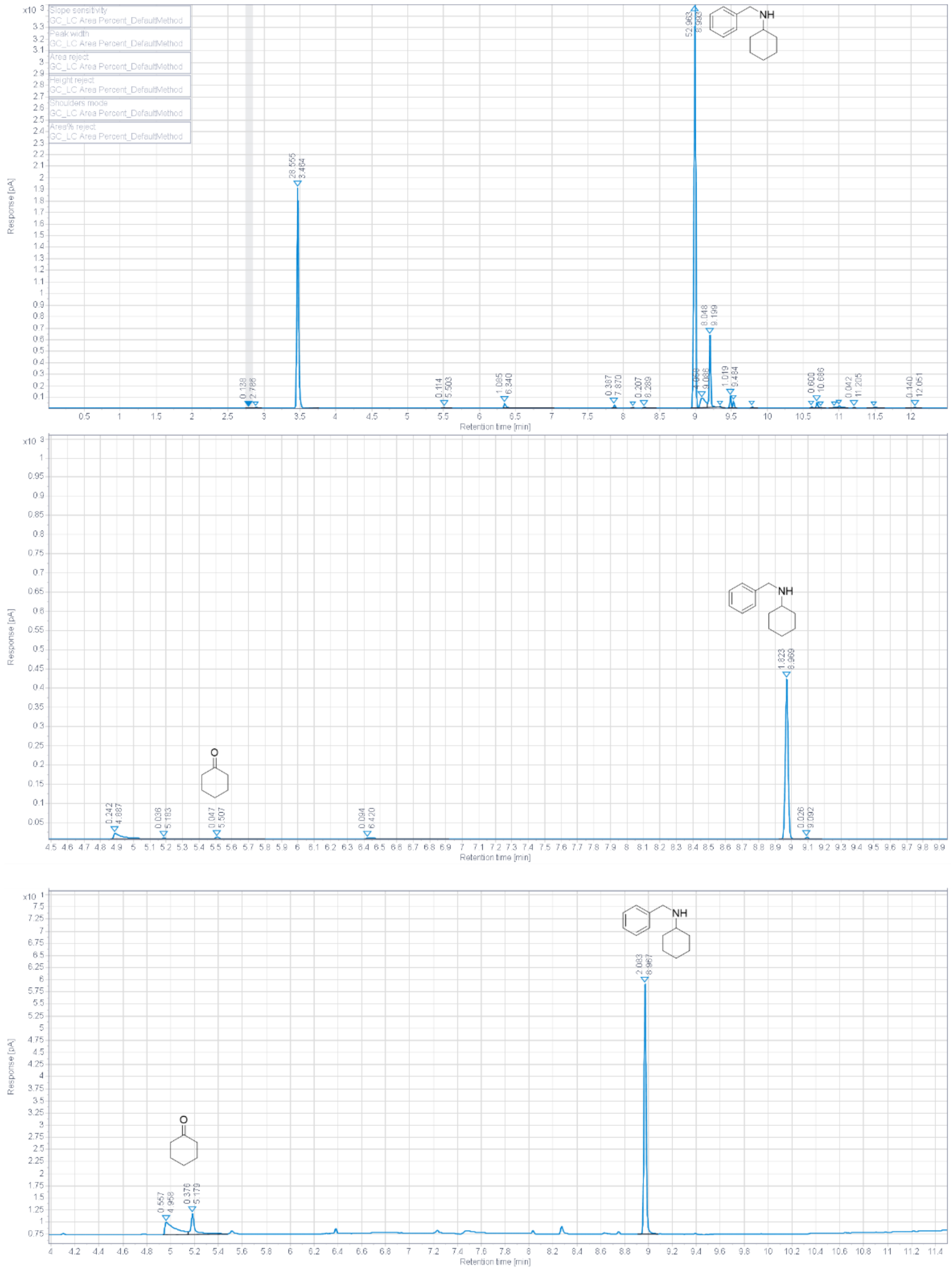

Figure S47: GC-FID spectra of: Top: analytical standard *N*-benzylcyclohexanamine; Middle: steady state of IR-79 single packed bed flow reaction; Bottom: *Pp*TA-IR79 packed-bed packed-bed flow reaction.

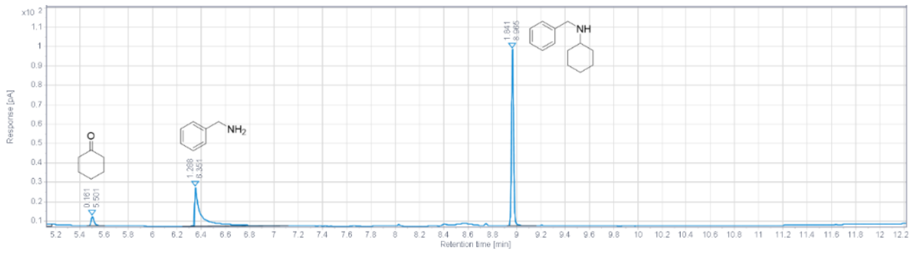

Figure S48: GC-FID spectra of steady state fractions from the GOase-*Vf*TA-IR79 MPIR-packed-bed packed-bed system.

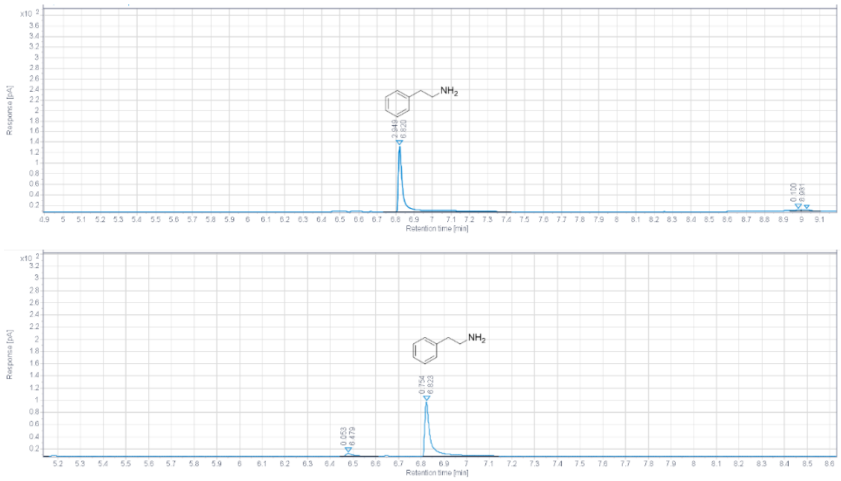

Figure S49: GC-FID spectra of: Top: analytical standard phenylethylamine; Bottom: steady state fractions of *Bm*TA flow amination of phenylethanal.

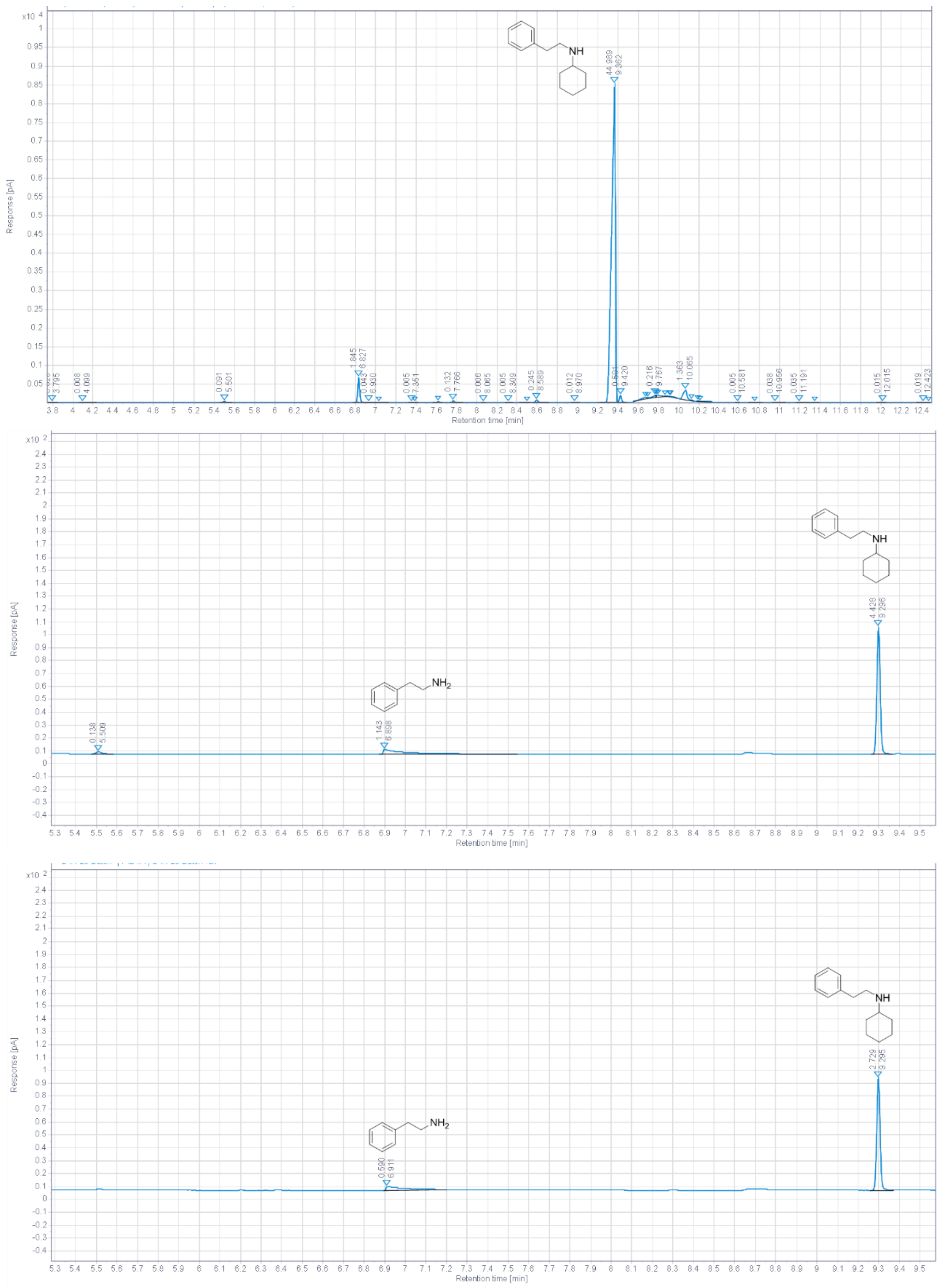

Figure S50: GC-FID spectra of: Top: analytical standard *N*-phenylethyl cyclohexanamine; Middle: steady state of IR-23 single packed bed flow reaction; Bottom: *Bm*TA-IR23 packed-bed packed-bed flow reaction.

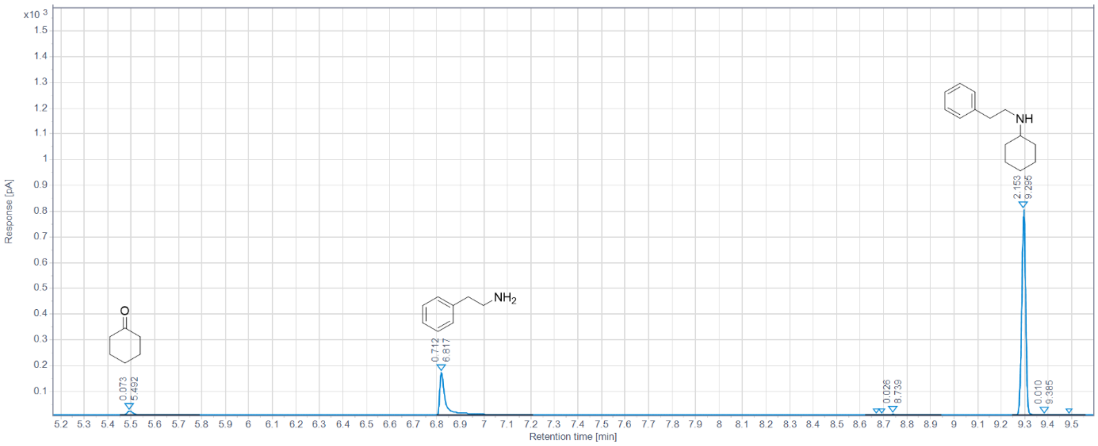

Figure S51: GC-FID spectra of steady state fractions from AcCO$_6$-*Bm*TA-IR23 MPIR-packed bed packed-bed flow reaction.

General procedure for synthesis of secondary amines

To a stirred solution of anhydrous THF (20 mL) at room temperature under an atmosphere of N_2, aldehyde (1 eq.) and amine (1.5 eq.) were added. Glacial acetic acid (1 eq.) and sodium triacetoxyborohydride (1.5 eq.) were added and the mixture was stirred for 24 hours. The reaction mixture was quenched by the addition of saturated $NaHCO_3$ solution (15 mL) and extracted into EtOAc (2 x 15 mL). The combined organic extracts were washed with aqueous HCl solution (1 M, 3 x 15 mL) and then the combined aqueous extracts were basified to pH = 12 with NaOH solution (5 M). The combined aqueous layers were then extracted with EtOAc (2 x 15 mL) and then dried over $MgSO_4$. The solvent was removed under reduced pressure to afford the final product.

***N*-benzylcyclopropanamine**

N-benzylcyclopropanamine was prepared as above and matched the literature values (*ACS Catal.*, 2019, **9**, 6738).

***N*-(4-methylbenzene)cyclopropylamine**

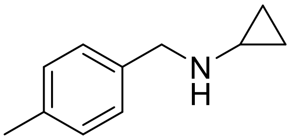

Yellow oil isolated, 59% yield (191 mg). **^{1}H NMR** δ_H (400 MHz, $CDCl_3$) 7.21 (d, J = 8.0 Hz, 2H), 7.14 (d, J = 7.8 Hz, 2H), 3.81 (s, 2H), 2.35 (s, 3H), 2.22 – 2.12 (m, 1H), 0.51 – 0.32 (m, 4H). **^{13}C NMR** δ_C (101 MHz, $CDCl_3$) 137.68 (C), 136.48 (C), 129.15 (2CH), 128.26 (2CH), 53.52 (CH_2), 30.09 (CH), 21.19 (CH_3), 6.54 ($2CH_2$); **GC-MS**: Expected mass [M-H] 160.1, observed mass 160.1.

***N*-(3-methylbenzene)cyclopropylamine**

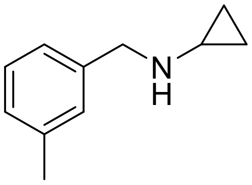

Yellow oil isolated, 68% yield (219 mg). **^{1}H NMR** δ_H (400 MHz, $CDCl_3$) 7.24 (t, J = 7.5 Hz, 1H), 7.17 – 7.11 (m, 2H), 7.08 (d, J = 7.5 Hz, 1H), 3.83 (s, 2H), 2.37 (s, 3H), 2.18 (ddd, J = 8.6, 6.6, 3.7 Hz, 1H), 0.51 – 0.39 (m, 4H). **^{13}C NMR** δ_C (101 MHz, $CDCl_3$) 140.60 (C), 138.03 (C), 129.03 (CH), 128.33 (CH), 127.66 (CH), 125.30 (CH), 53.80 (CH_2), 30.18 (CH), 21.47 (CH_3), 6.54 ($2CH_2$). **GC-MS**: Expected mass [M-H] 160.1, observed mass 160.1.

***N*-(4-fluorobenzene)cyclopropylamine**

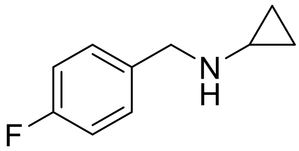

Yellow oil isolated, 55% yield (182 mg). **^{1}H NMR** δ_H (400 MHz, $CDCl_3$) 7.31 – 7.26 (m, 2H), 7.05 – 6.98 (m, 2H), 3.82 (s, 2H), 2.20 – 2.10 (m, 1H), 0.51 – 0.31 (m, 4H). **^{13}C NMR** δ_C (101 MHz, $CDCl_3$) 161.98 (C, d, J = 244.6 Hz), 136.46 (C, d, J = 3.0 Hz), 129.83 (2CH, d, J = 8.0 Hz), 115.19 (2CH, d, J = 21.2 Hz), 53.10 (CH_2), 30.13 (CH), 6.55 ($2CH_2$); **GC-MS**: Expected mass [M-H] 164.1, observed mass 164.1.

***N*-(3-fluorobenzene)cyclopropylamine**

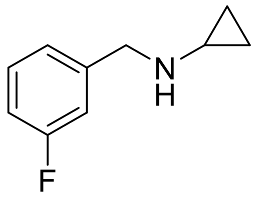

Yellow oil isolated, 57% (187 mg). **^{1}H NMR** δ_H (400 MHz, CDCl3) 7.27 (td, J = 7.8, 5.9 Hz, 1H), 7.08 (d, J = 7.6 Hz, 1H), 7.03 (dt, J = 9.8, 2.1 Hz, 1H), 6.93 (td, J = 8.3, 2.3 Hz, 1H), 3.83 (s, 2H), 2.18 – 2.11 (m, 1H), 0.48 – 0.35 (m, 4H). **^{13}C NMR** δ_C (101 MHz, CDCl3) 162.97 (C, d, J = 245.5 Hz), 143.38 (C, d, J = 6.8 Hz), 129.77 (CH, d, J = 8.3 Hz), 123.67 (CH, d, J = 2.8 Hz), 114.97 (CH, d, J = 21.0 Hz), 113.67 (CH, d, J = 21.2 Hz), 53.21 (CH_2, d, J = 1.8 Hz), 30.04 (CH), 6.50 ($2CH_2$); **GC-MS**: Expected mass [M-H] 164.1, observed mass 164.1.

NMR Spectra

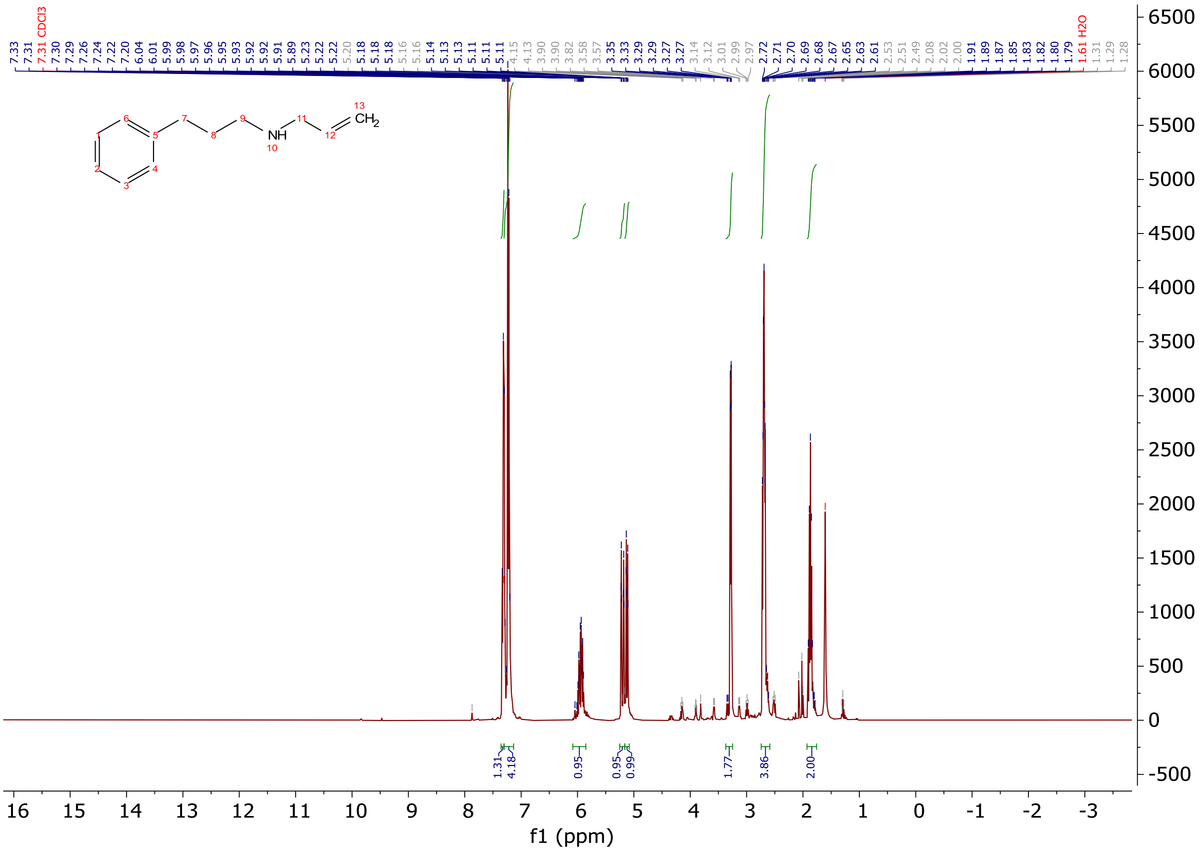

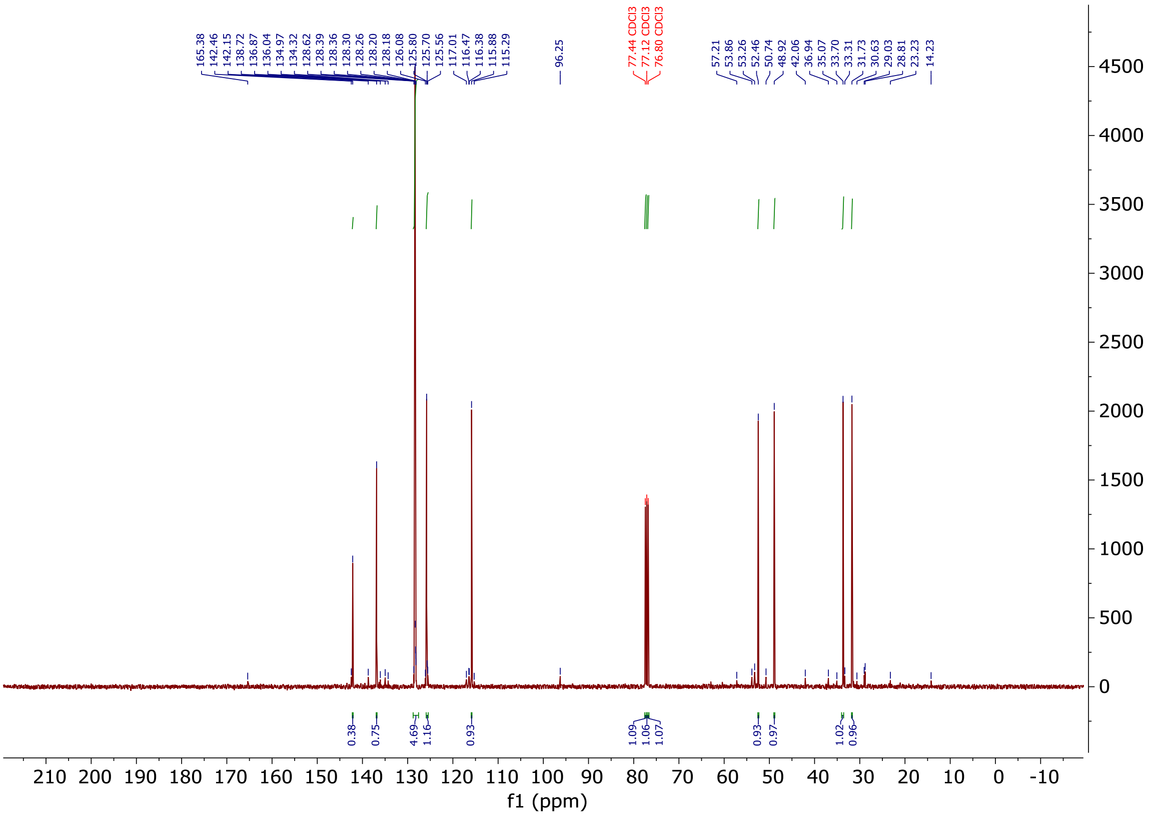

Figure S52: ^{1}H-NMR spectrum (top) and ^{13}C-NMR spectrum (bottom) of *N*-hydrocinnamlyallylamine.

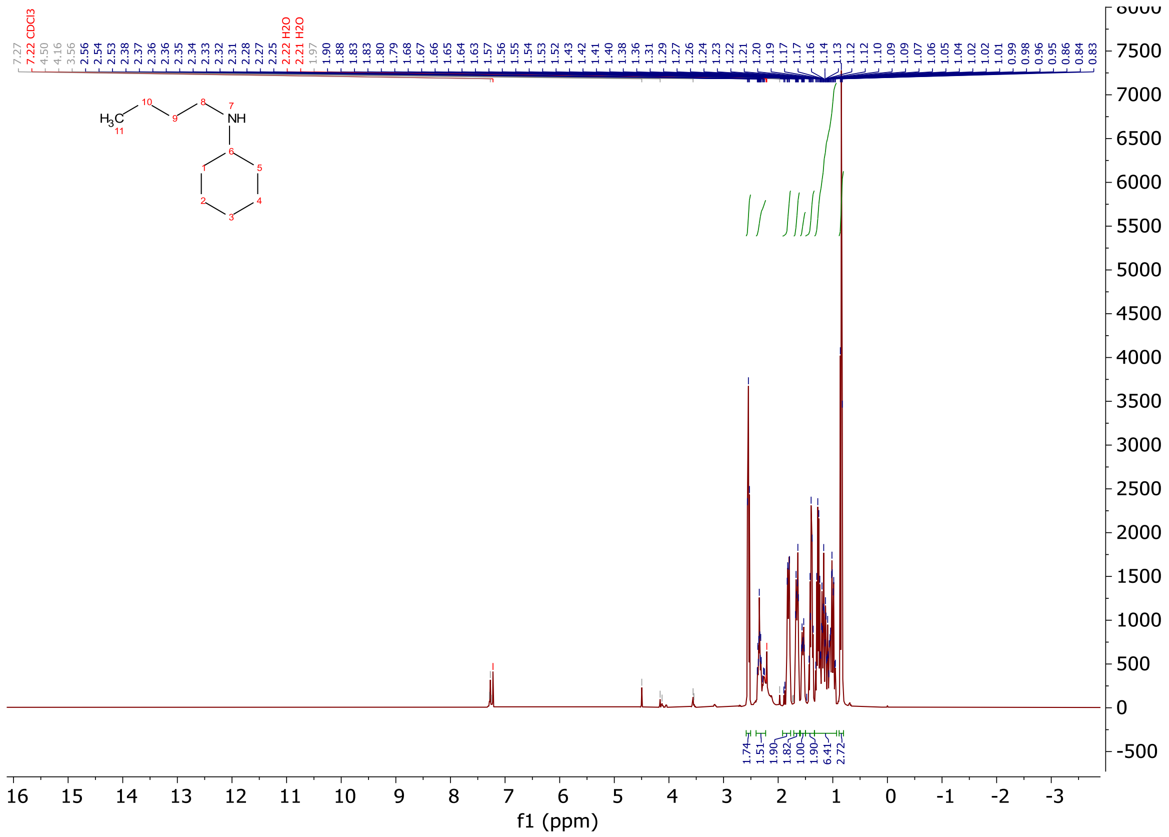

Figure S53: ^{1}H-NMR spectrum of *N*-butylcyclohexanamine.

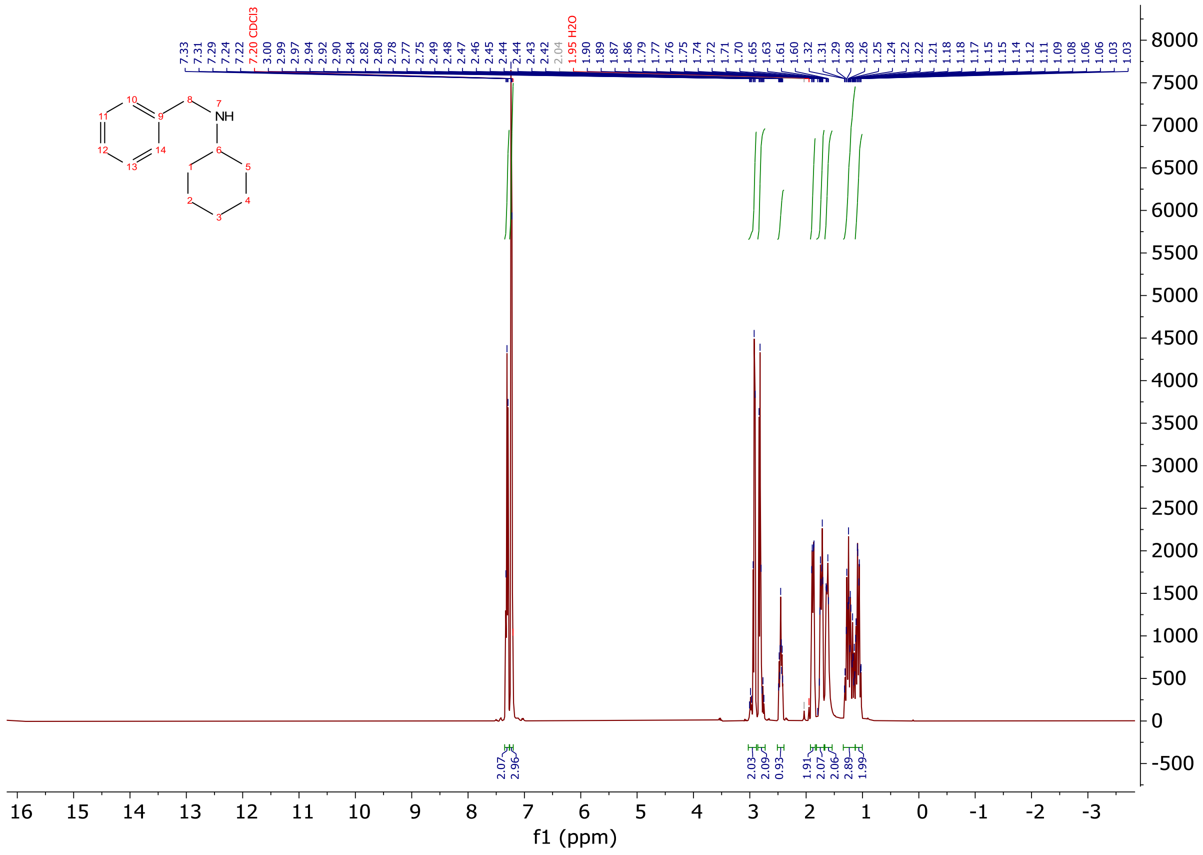

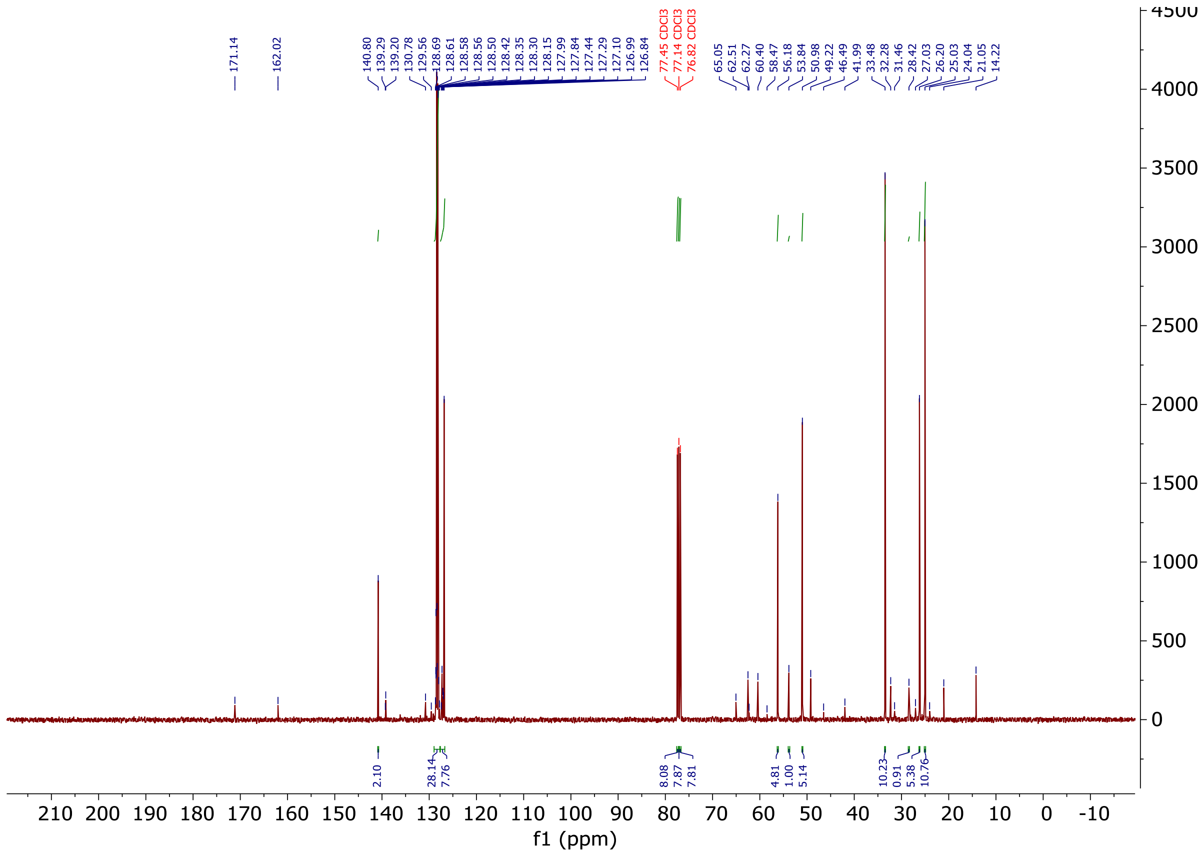

Figure S54: ^{1}H-NMR spectrum (top) and ^{13}C-NMR spectrum (bottom) of *N*-benzyl cyclohexanamine.

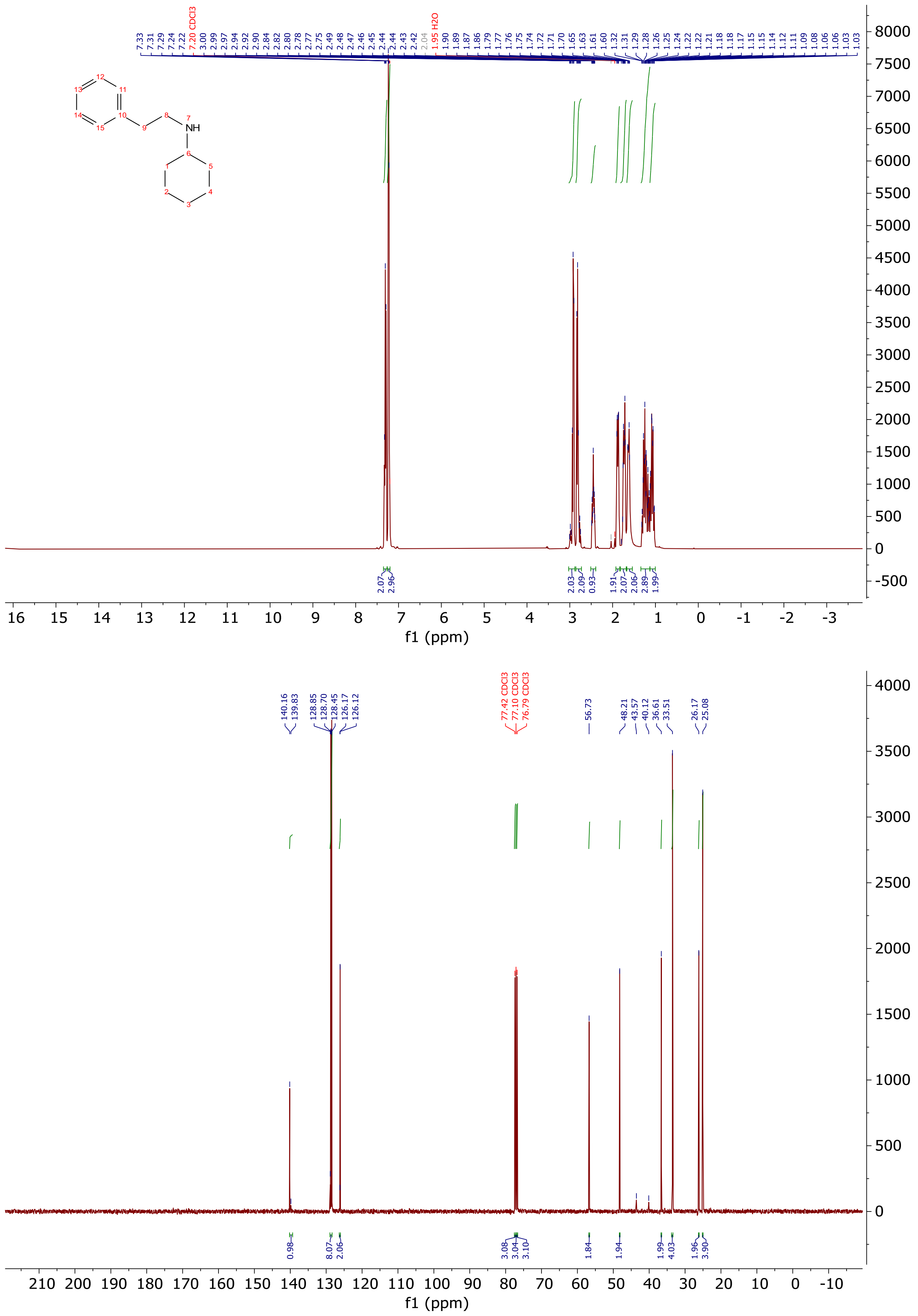

Figure S55: ^{1}H-NMR spectrum (top) and ^{13}C-NMR spectrum (bottom) of *N*-phenylethyl cyclohexanamine.

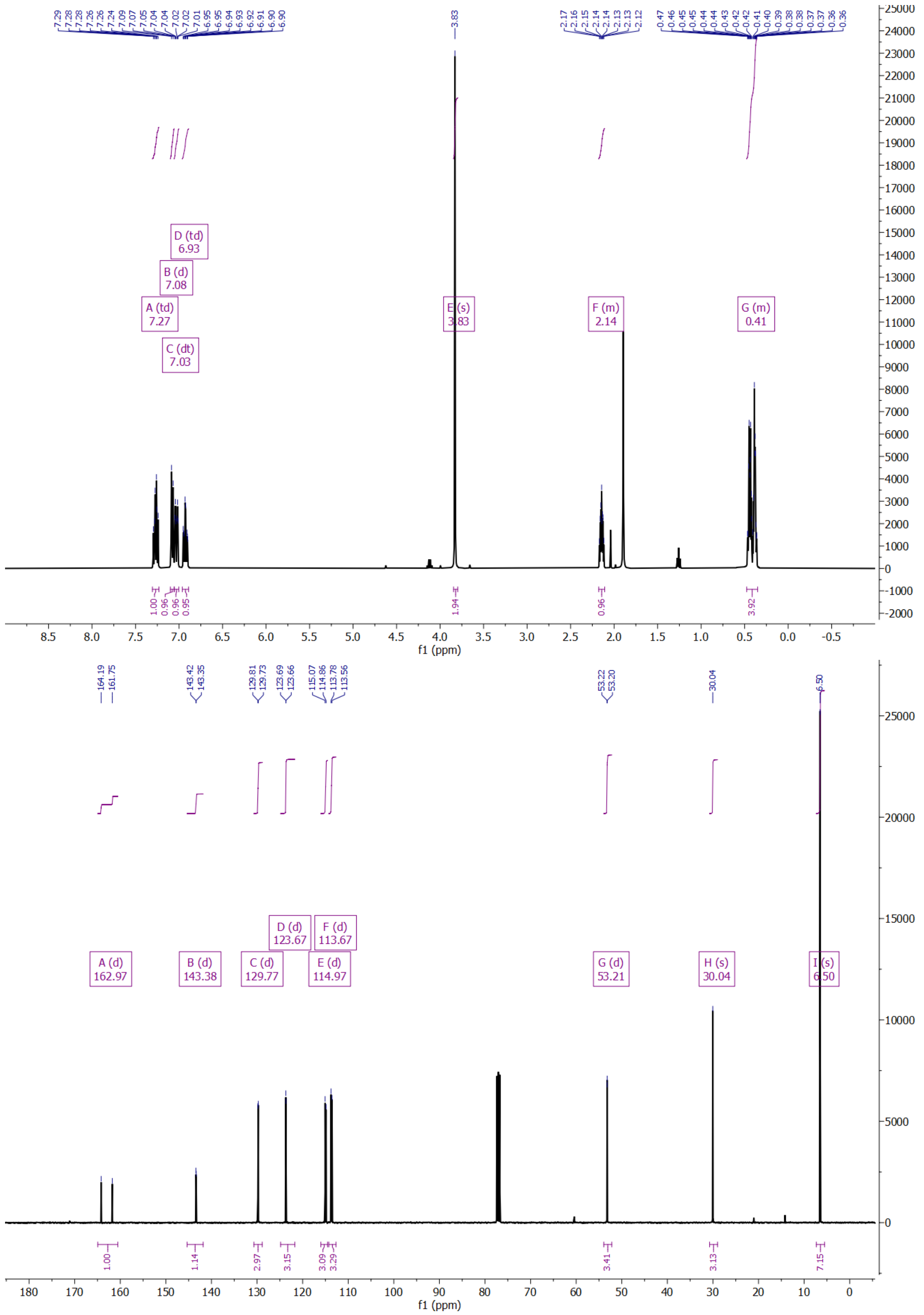

Figure S56: ^{1}H NMR spectrum (top) and ^{13}C NMR spectrum (bottom) of *N*-(3-fluorobenzene)cyclopropylamine.

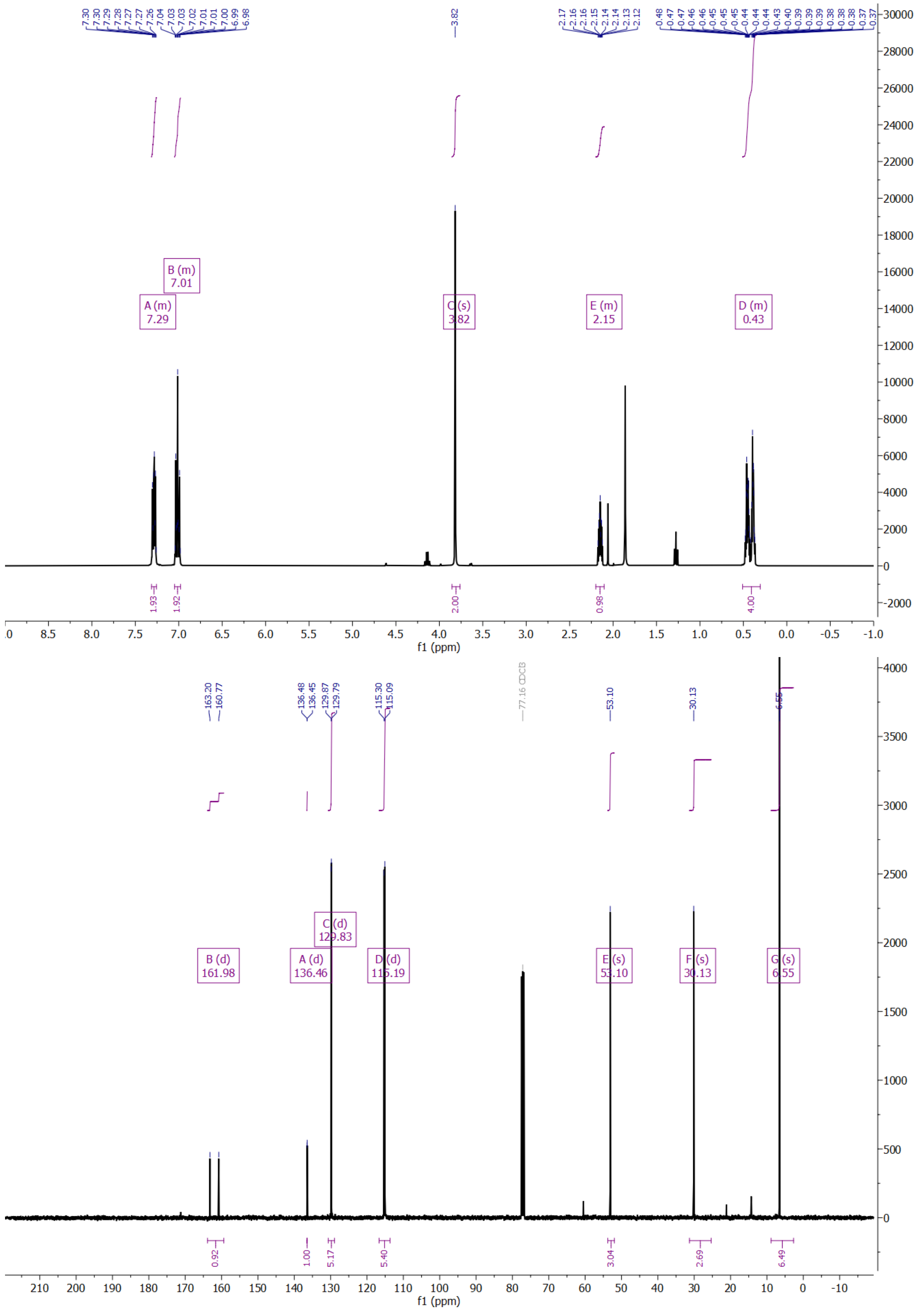

Figure S57: ^{1}H NMR spectrum (top) and ^{13}C NMR spectrum (bottom) of *N*-(4-fluorobenzene)cyclopropylamine.

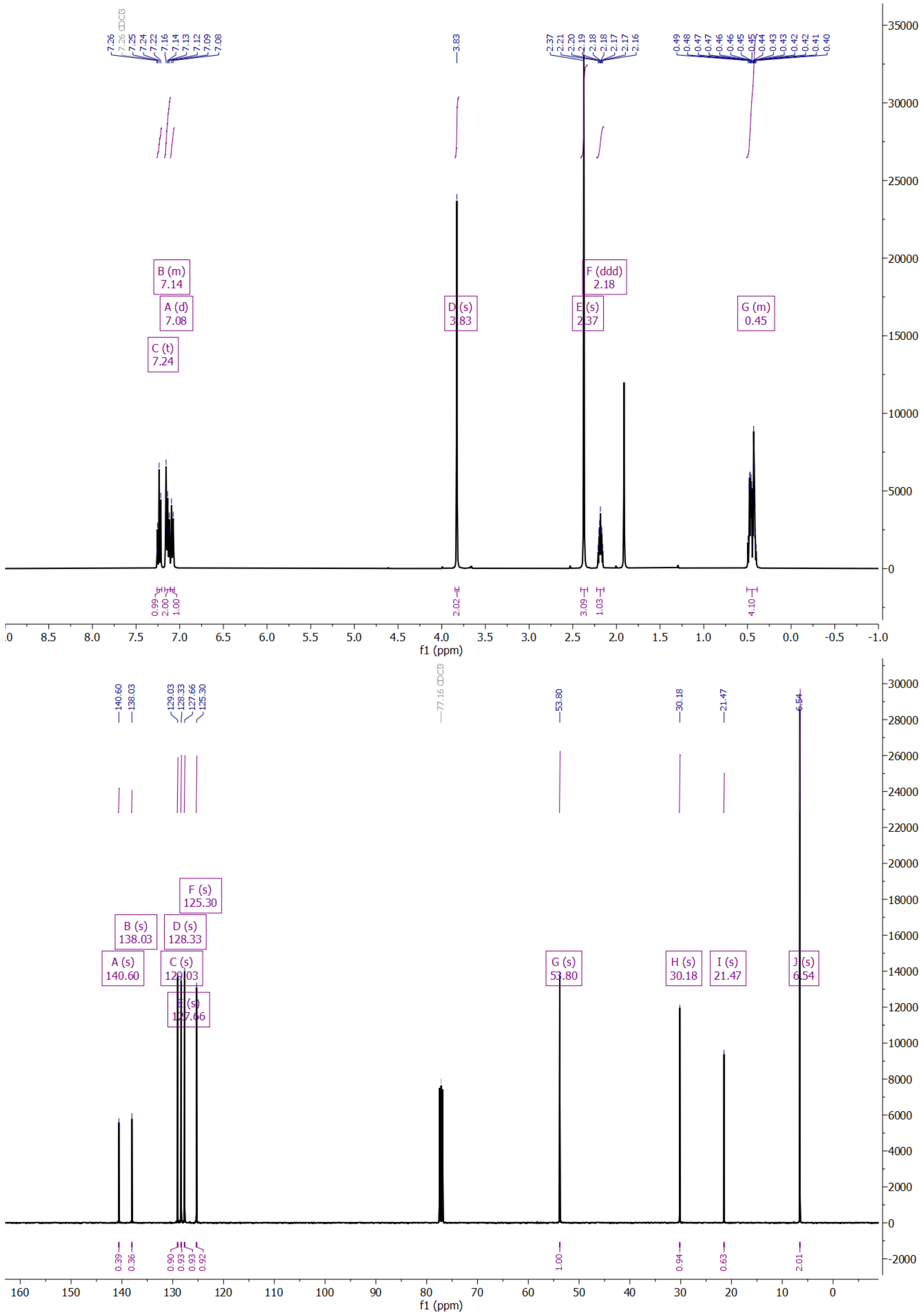

Figure S58: ^{1}H NMR spectrum (top) and ^{13}C NMR spectrum (bottom) of *N*-(3-methylbenzene)cyclopropylamine.

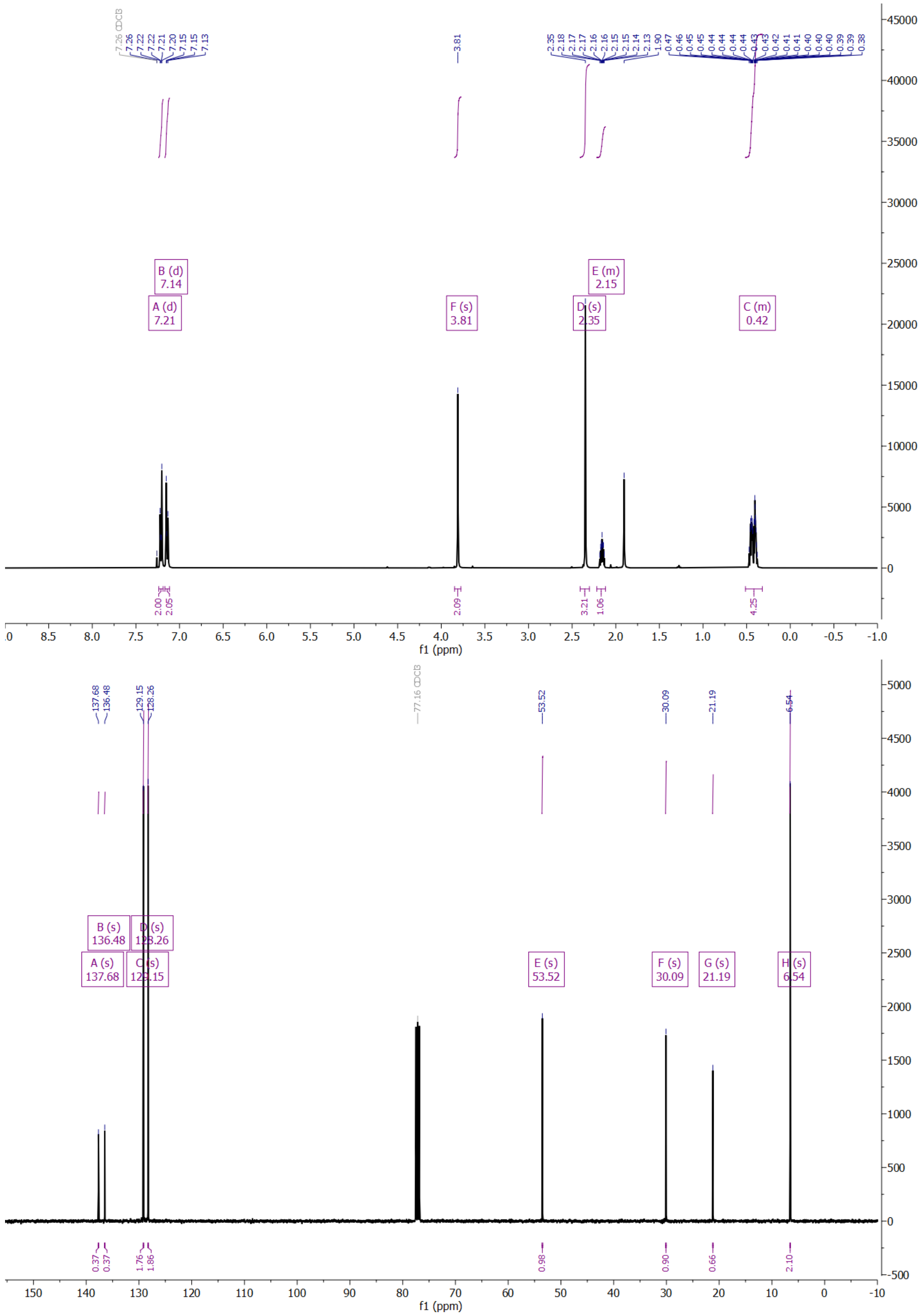

Figure S59: ^{1}H NMR spectrum (top) and ^{13}C NMR spectrum (bottom) of *N*-(4-fluorobenzene)cyclopropylamine.

Synthesis of 4-OMethylnorbelladine

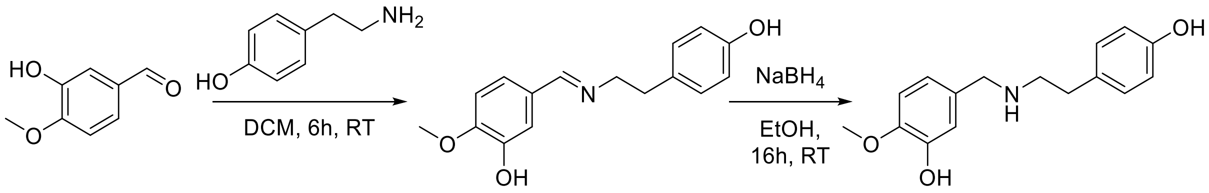

3-hydroxy-4-hydroxymethyl benzaldehyde (304 mg, 2.0 mmol) was dissolved in dichloromethane (8 mL) before addition of tyramine (274 mg, 2.0 mmol). The solution was stirred at ambient temperature for 6 hours before concentrating *in vacuo*. The resulting solid was dissolved in methanol (8 mL) and sodium borohydride (76 mg, 2.0 mmol) was added. The solution was subsequently stirred at ambient temperature for 16 hours. The reaction mixture was concentrated *in vacuo* and the crude material re-suspended in water (10 mL). The suspension was filtered and the solid dissolved in methanol before concentrating *in* vacuo to yield a brown powder (449 mg, 82%); data consistent with those reported in the literature; [4]

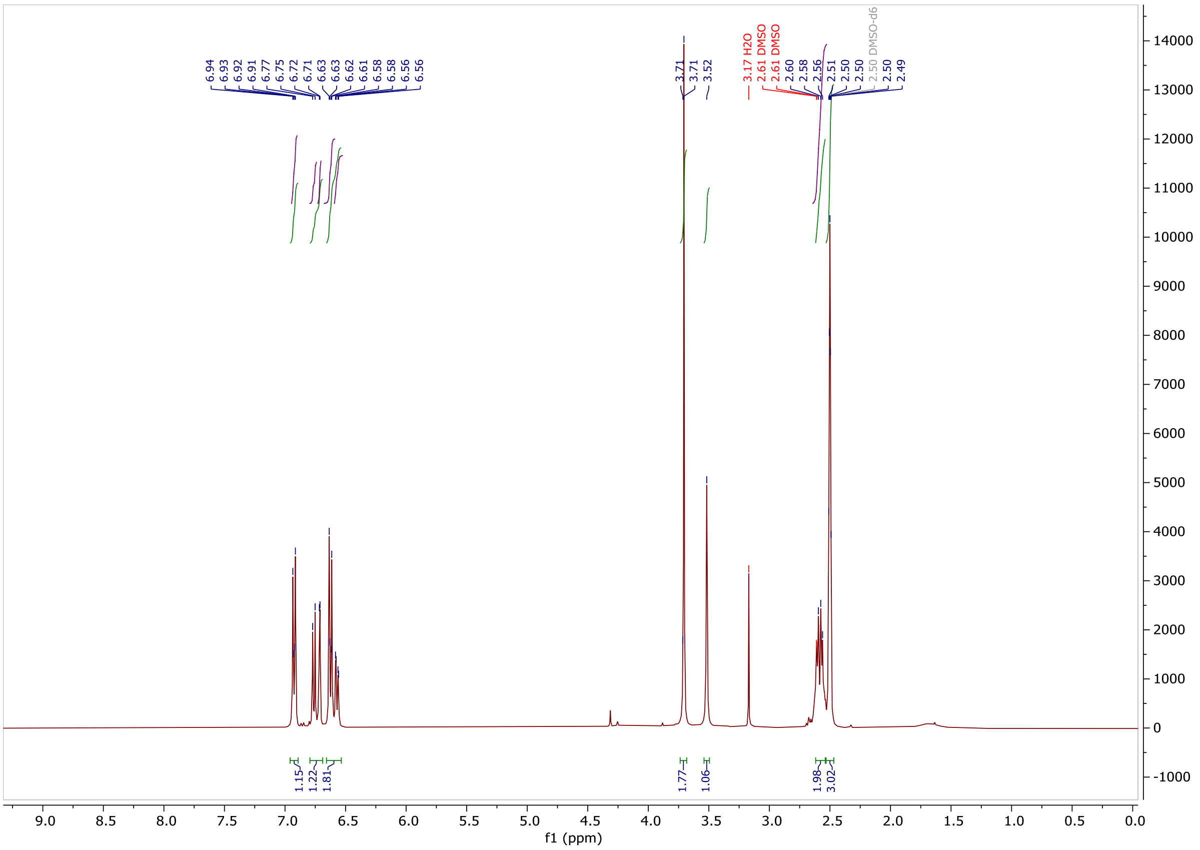

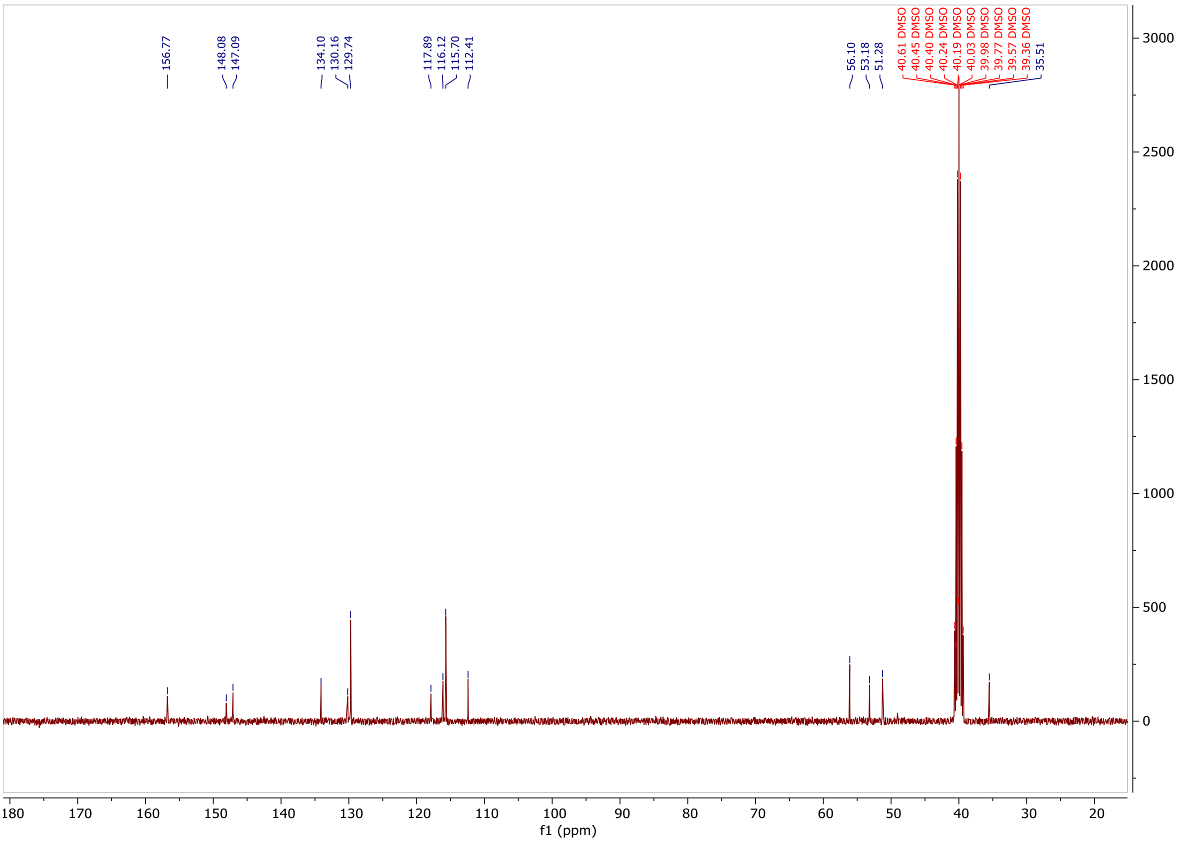

Figure S60: ^{1}H-NMR spectrum (top) and ^{13}C-NMR (bottom) spectrum of 4-OMethylmorbelladine.

References

1. Montgomery, S. L. *et al.* Characterization of imine reductases in reductive amination for the exploration of structure-activity relationships. *Sci. Adv.* **6**, eaay9320 (2020).

2. Chapman, M. R., Cosgrove, S. C., Turner, N. J., Kapur, N. & Blacker, A. J. Highly Productive Oxidative Biocatalysis in Continuous Flow by Enhancing the Aqueous Equilibrium Solubility of Oxygen. *Angew. Chem. Int. Ed.* **57**, 10535–10539 (2018).

3. Marshall, J. R. *et al.* Screening and characterization of a diverse panel of metagenomic imine reductases for biocatalytic reductive amination. *Nat. Chem.* (2020) doi:10.1038/s41557-020-00606-w.

4. Singh, A. *et al.* Cloning and characterization of norbelladine synthase catalyzing the first committed reaction in Amaryllidaceae alkaloid biosynthesis. *BMC Plant Biol.* **18**, (2018).
